# A symbolic Neanderthal accumulation of large herbivore crania

**DOI:** 10.1038/s41562-022-01503-7

**Published:** 2023-01-26

**Authors:** Enrique Baquedano, Juan L. Arsuaga, Alfredo Pérez-González, César Laplana, Belén Márquez, Rosa Huguet, Sandra Gómez-Soler, Lucía Villaescusa, M. Ángeles Galindo-Pellicena, Laura Rodríguez, Rebeca García-González, M.-Cruz Ortega, David M. Martín-Perea, Ana I. Ortega, Lucía Hernández-Vivanco, Gonzalo Ruiz-Liso, Juan Gómez-Hernanz, José I. Alonso-Martín, Ana Abrunhosa, Abel Moclán, Ana I. Casado, Marina Vegara-Riquelme, Ana Álvarez-Fernández, Ángel C. Domínguez-García, Diego J. Álvarez-Lao, Nuria García, Paloma Sevilla, Hugues-Alexandre Blain, Blanca Ruiz-Zapata, M. José Gil-García, Adrián Álvarez-Vena, Teresa Sanz, Rolf Quam, Tom Higham

**Affiliations:** 1 Museo Arqueológico y Paleontológico de la Comunidad de Madrid, Alcalá de Henares, Spain; 2 Institute of Evolution in Africa, Madrid, Spain; 3 Department of Geodynamics, Stratigraphy and Palaeontology, Faculty of Geology, Complutense University of Madrid, Madrid, Spain; 4 UCM-ISCIII Research Centre for Human Evolution and Behaviour, Madrid, Spain; 5 IPHES-CERCA, Institut Català de Paleoecologia Humana i Evolució Social, Tarragona, Spain; 6 Departament d’Història i Història de l’Art, University Rovira i Virgili, Tarragona, Spain; 7 Unit associated with CSIC, Departamento de Paleobiología, Museo Nacional de Ciencias Naturales, Madrid, Spain; 8 Department of Geology, Geography and Environment, University of Alcalá, Alcalá de Henares, Spain; 9 University of Alcalá General Foundation, Alcalá de Henares, Spain; 10 Department of Biodiversity and Environmental Management, University of León, León, Spain; 11 Laboratory of Human Evolution, Faculty of Humanities and Communication, University of Burgos, Burgos, Spain; 12 Palaeobiology Department, National Natural Sciences Museum—CSIC, Madrid, Spain; 13 National Research Centre for Human Evolution (FA-CENIEH), Burgos, Spain; 14 Fundación Atapuerca, Ibeas de Juarros, Burgos, Spain; 15 Department History and Philosophy, Area of Prehistory, University of Alcalá, Alcalá de Henares, Spain; 16 Interdisciplinary Centre for Archaeology and Evolution of Human Behaviour, University of Algarve, Faro, Portugal; 17 Escuela de Posgrado, Universidad de Burgos, Burgos, Spain; 18 Physical Chemistry Department, Faculty of Chemical Sciences, Complutense University of Madrid, Madrid, Spain; 19 Institute of Geosciences (IGEO, UCM-CSIC), Madrid, Spain; 20 Geology Department, Oviedo University, Oviedo, Spain; 21 Department of Anthropology, Binghamton University (SUNY), Binghamton, NY USA; 22 Division of Anthropology, American Museum of Natural History, New York, NY USA; 23 Cátedra de Otoacústica Evolutiva y Paleoantropología (HM Hospitales-Universidad de Alcalá), Departamento de Ciencias de la Vida, Universidad de Alcalá, Madrid, Spain; 24 Oxford Radiocarbon Accelerator Unit, Research Laboratory for Archaeology and the History of Art, University of Oxford, Oxford, UK; 25 Department of Evolutionary Anthropology, University of Vienna, Vienna, Austria; 26 Human Evolution and Archaeological Sciences Forschungsverbund, University of Vienna, Vienna, Austria

**Keywords:** Archaeology, Anthropology, Cultural evolution, Archaeology

## Abstract

This work examines the possible behaviour of Neanderthal groups at the Cueva Des-Cubierta (central Spain) via the analysis of the latter’s archaeological assemblage. Alongside evidence of Mousterian lithic industry, Level 3 of the cave infill was found to contain an assemblage of mammalian bone remains dominated by the crania of large ungulates, some associated with small hearths. The scarcity of post-cranial elements, teeth, mandibles and maxillae, along with evidence of anthropogenic modification of the crania (cut and percussion marks), indicates that the carcasses of the corresponding animals were initially processed outside the cave, and the crania were later brought inside. A second round of processing then took place, possibly related to the removal of the brain. The continued presence of crania throughout Level 3 indicates that this behaviour was recurrent during this level’s formation. This behaviour seems to have no subsistence-related purpose but to be more symbolic in its intent.

## Abstract (web summary)

During the Last Ice Age, Neanderthals used a small cave in the Iberian Peninsula to accumulate the crania of large ungulates (bison, aurochs, red deer and rhinoceroses), some associated with small hearths. This seems to have been a symbolic practice.

## Main

Evidence of the past presence of Neanderthals at archaeological sites is usually associated with subsistence activities, such as hunting, the processing and consumption of animal resources, the preparation of tools or the use of fire^1–6^. Less frequently, however, their presence can be associated with other functions, such as extractive activities (for example, flint quarrying^7^) and activities related to their symbolic world (for example, burials^8–11^ and the use of structures constructed for possible ceremonial use^12^). The present work examines an unusual archaeological assemblage, an accumulation of crania belonging to large mammals, apparently processed by Neanderthals, in Level 3 of the Cueva Des-Cubierta (in central Spain). Taxonomic and anatomical analyses of this assemblage, along with the taphonomic modifications to which its components were subjected, indicate that its origin lies in something other than practices associated with mere subsistence. Rather, it is probably associated with Neanderthal symbolism.

## Results

### Archaeological context

The Cueva Des-Cubierta (coordinates, 40°55′23″N 3°48′29″W, WGS84 datum; altitude, 1,112 m) forms part of a multi-level karstic system consisting of subhorizontal conduits in outcrops of Cretaceous marine carbonate running along the right bank of the upper River Lozoya Valley, in the north of the Madrid Region (Spain). It was discovered in 2009 during survey work performed within the framework of the archaeological activity undertaken in the area since 2002^13–15^. The cave, which runs zigzag for some 80 m and is 2–4 m wide, has lost its ceiling due to the erosive dismantling of its dolomite (Supplementary Results, ‘The site’).

**Extended Data Fig. 1:**
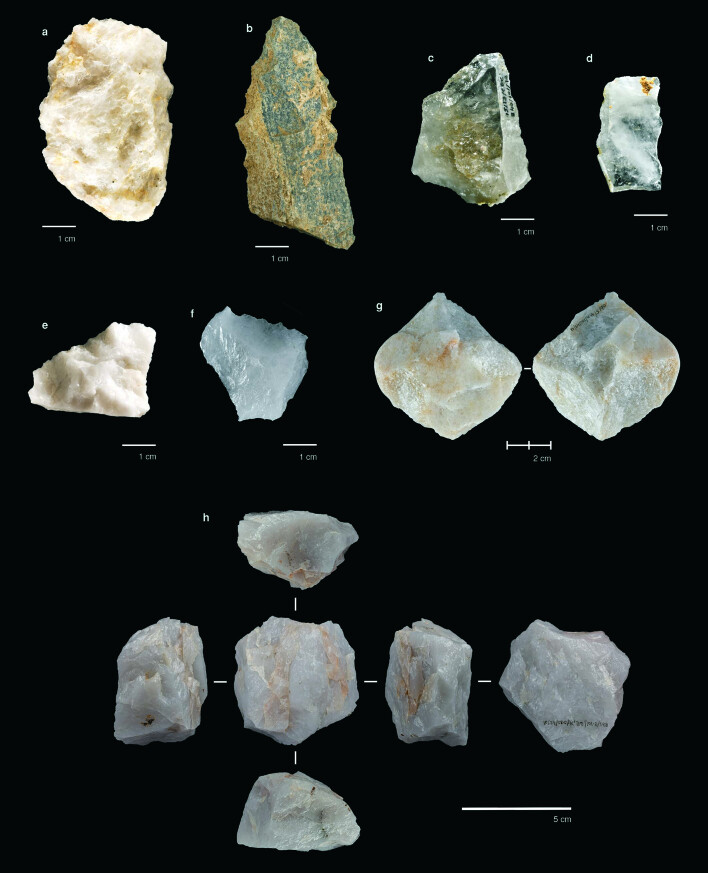
Mousterian lithic industry from Levels 2 and 3, Cueva Des-Cubierta. a) Quartz denticulate (10/19/CDC/H’49/2/125) (Level 3); b) Convergent denticulate in rhyolite (19/34/CDC/K’39/101/18) (Level 3); c) Denticulate point in quartz (15/13/CDC/I’40/101/12) (Level 3); d) Quartz scraper (15/13/CDC/I’42/101/131) (Level 3); e) Quartz denticulate (10/19/CDC/I’41/1/3) (Level 3); f) Quartz denticulate (19/34/CDC/G’46/5/36) (Level 2); g) Quartz bifacial core in a mid state of exploitation (19/34/CDC/G’47/5/70) (Level 2); h) Quartz trifacial core (18/29/CDC/K’38/101.2/193) (Level 3). Photo credit: Mario Torquemada/MAPCM (a, c, d, and e) and Alfonso Dávila (b, f, g and h).

In the main gallery, an immature human mandible and six deciduous teeth have been recovered from Level 2 (ref. ^16^) (see also Supplementary Results, ‘Human remains’). The developmental stage of these teeth and the developing permanent tooth germ within the body of the mandible suggest (by modern standards) that these items belonged to a single individual who died at the age of 3–5 years^17^ (see also Supplementary Results, ‘Human remains’). The lack of a bony chin, the degree of shovelling and the crown outline of the molars are typically Neanderthal^18,19^ (see also Supplementary Results, ‘Human remains’). In addition, the lithic industry represented in Level 2 (*n* = 734 elements), made mainly of quartz, is clearly Mousterian. Despite the peculiarities of quartz knapping, the use of discoid knapping for flake extraction, the presence of denticulates and notches among the retouched elements, and the absence of any large cutting tools or laminar elements confirm the Mousterian character of these elements (Extended Data Fig. 1 and Supplementary Results, ‘Lithics’).

The underlying Level 3 contains an accumulation of clast-supported subangular dolomite blocks and carbonate boulders in a sand–clay matrix. Covering some 27 m^2^ and reaching 2 m in depth, it houses the archaeological remains discussed in this work. Its biochronological context and available dating evidence place this level within MIS4 or the first half of MIS3 (Supplementary Results, ‘Faunal remains’, ‘Radiocarbon dating’ and ‘U/Th datings’). The pollen record suggests the climate to have been drier and colder than at present (Supplementary Results, ‘Palynology’). Plant diversity was limited, woodland development was patchy, and *Juniperus* and other steppe plants were in expansion at this time. These environmental conditions agree with those suggested by a study of the level’s association of micromammals, which is dominated by different species of vole (*Microtus arvalis*, *M.* gr. *agrestis* and *M.* gr. *lusitanicus-duodecimcostatus*) plus some remains of *Ochotona* cf. *pusilla*, all indicators of an open landscape with little forest (Supplementary Results, ‘Faunal remains’). The presence of type T-55 and 7A spores, which are associated with fire, agrees with the recognition of combustion areas in Level 3 (Supplementary Results, ‘Palynology’).

**Extended Data Fig. 2:**
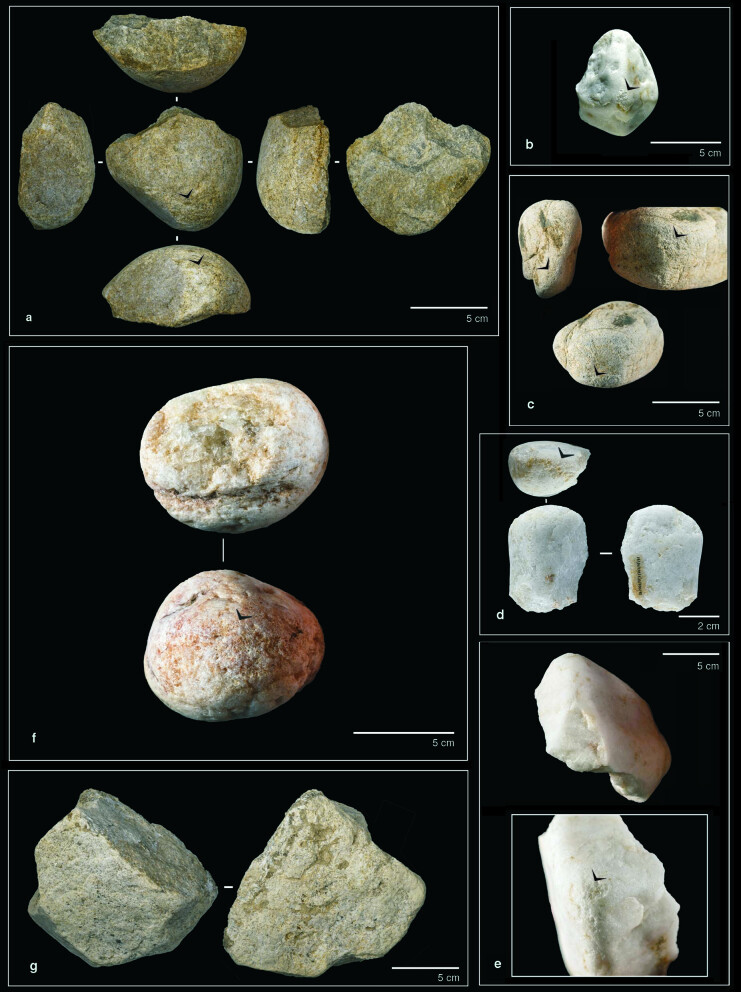
Cores, hammerstones and anvils. Levels 2 and 3, Cueva Des-Cubierta. Black arrows on each tool point to battering marks. a) Quartz hammerstone re-used as a core (17/26/CDC/101/K’37/29) (Level 3); b) Detail of battering on the back of a bifacial core of orthogonal bipolar direction (16/12/CDC/E’48/Brecha5/32) (Level 2); c) Granitic hammerstone with different battered surfaces, associated with a cranium (18/ 29/CDC/H’44/101/68) (Level 3); d) Quartz hammerstone re-used as a unifacial core (19/34/L’39/100/98) (Level 3); e) Hammerstone fragment (18/29/CDC/K’38/101/2). Battering marks (Level 3); f) Broken quartz hammerstone (18/29/CDC/H’44/101.2/121) (Level 3); g) Tabular quartz anvil (17/26/CDC/101/I’43/40) (Level 3). Photo credit: Alfonso Dávila (c, e and f) and Mario Torquemada/MAPCM (a, d).

Level 3 also contains ample evidence of lithic industry. In total, 1,421 anvils, hammerstones, cores, flakes and shaped tools have been recovered, all belonging to the Neanderthal Mousterian technocomplex (Extended Data Figs. 1 and 2 and Supplementary Results, ‘Lithics’). The most common raw materials are those available locally. Quartz stands out for its abundance, and gneiss for the size of the pieces made from it, particularly hammerstones and anvils. Broken hammerstones were frequently reused as cores. The most used knapping methods were expeditive, followed by centripetal and orthogonal methods used in both bifacial and unifacial manners. Among the shaped tools present, denticulates and notches are the most common, followed by sidescrapers and retouched flakes. The presence of debris and refittings confirm that some lithic tools were configured inside the cave during the formation of Level 3.

**Extended Data Fig. 3:**
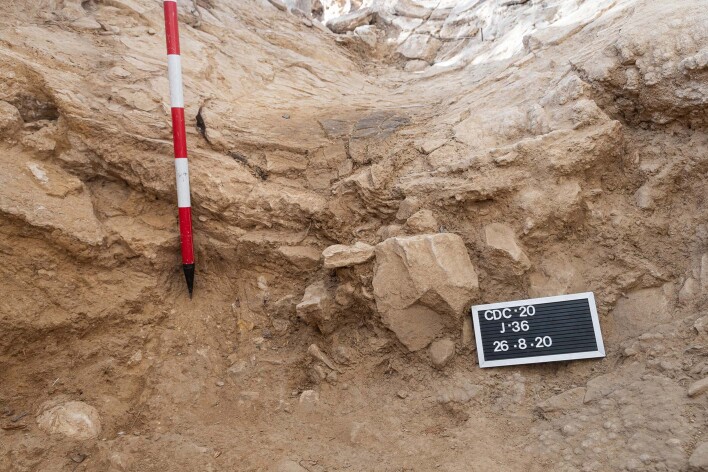
Top of the S1 speleothem with evidence of thermal alteration. The photograph shows square J'36 of the Cueva Des-Cubierta after having been excavated up to the top of speleothem S1. The dark spot indicates the thermal alteration experienced by the upper part of this speleothem. At this location, the base of Level 3 (completely excavated at the time of taking the photograph) rested directly on S1. The thermal alteration of the top of speleothem S1 is therefore evidence of the existence of hearths inside the cavity during the deposition of Level 3 (The Supplementary Figs. 31 to 39 include more information about the heat-altered elements in spatial association with this speleothem dark spot). Photo credit: Alfonso Dávila.

Signs of thermoalteration were noted on elements of lithic industry (1.1% of the lithic remains recovered), on dolomite clasts (13.0% of all such clasts documented) and occasionally on the remains of micromammals (Supplementary Results, ‘Evidences of fire’). Charcoals (*n* = 338) were also present in the assemblage. Overall, 34.0% of the archaeological record of Level 3 was affected by fire. Although it was hard to find conserved combustion-associated structures in the sedimentary matrix^20^, the documentation and spatial analysis of the thermoaltered remains allowed the identification of concentrations of fire-affected materials and specific points of combustion, including an area in the cave that conserved a directly burned speleothemic floor (the top of speleothem S1 in contact with Level 3) (Extended Data Fig. 3 and Supplementary Figs. 31–39). This shows that fires were made inside the cave.

### The large mammal assemblage

A total of 2,265 faunal remains over 2 cm in length have been recovered from Level 3, of which 1,616 have been identified taxonomically (Supplementary Results, ‘Faunal remains’). Ungulate remains dominate over those of carnivores (the carnivore-to-ungulate number of identified specimens (NISP) ratio is 1.6%^21^). The best represented of these ungulates are bovines (*Bison priscus* and *Bos primigenius*), followed at some distance by cervids (*Cervus elaphus* and *Capreolus capreolus*), steppe rhinoceroses (*Stephanorhinus hemitoechus*) and horses (*Equus ferus*) (Table 1). Analysis of the bone modification surfaces revealed these remains to show very few signs of predator activity (0.2% show furrowing); those that were detected were always found on post-cranial elements of the skeleton. The scarcity and poor definition of these modifications made it impossible to identify the predator involved (Supplementary Results, ‘Taphonomy’).

**Table 1:** Quantification of the abundance of large mammal species in Level 3

LEVEL 3	NISP	%NISP	MNE	%MNE	MNI	%MNI
*Felis silvestris*	4	0.25	3	2	1	2.33
*Panthera spelaea*	8	0.50	8	5.33	2	4.65
*Crocuta crocuta*	1	0.06	1	0.67	1	2.33
*Cuon alpinus*	3	0.19	2	1.33	1	2.33
*Ursus* cf. *arctos*	3	0.19	3	2	1	2.33
*Mustela* sp.	1	0.06	1	0.67	1	2.33
Carnivora indet.	6	0.37	1	0.67	1	2.33
*Equus ferus*	2	0.12	2	1.33	1	2.33
*Stephanorhinus hemitoechus*	59	3.66	8	5.33	2	4.65
*Capreolus capreolus*	6	0.37	5	3.33	2	2.33
*Cervus elaphus*	49	3.04	10	5.33	5	6.98
*Bos/Bison*	1,471	91.20	108	72	28	65.12
Total	1,613	100	150	100	43	100
Big mammals	431					
Medium-sized mammals	213					
Small mammals	5					
Indeterminable	1,314					

%NISP, relative frequency of the NISP; MNE, minimum number of elements; %MNE, relative frequency of the MNE; MNI, minimum number of individuals; %MNI, relative number of the MNI.

Some of the bone remains showed signs of thermoalteration (30.5%). Bones measuring 2–5 cm were the most affected, and carbonization was the most documented type of impact. Cranial and post-cranial remains were affected to much the same extent (around 20%) (Supplementary Results, ‘Evidences of fire’).

**Fig. 1:**
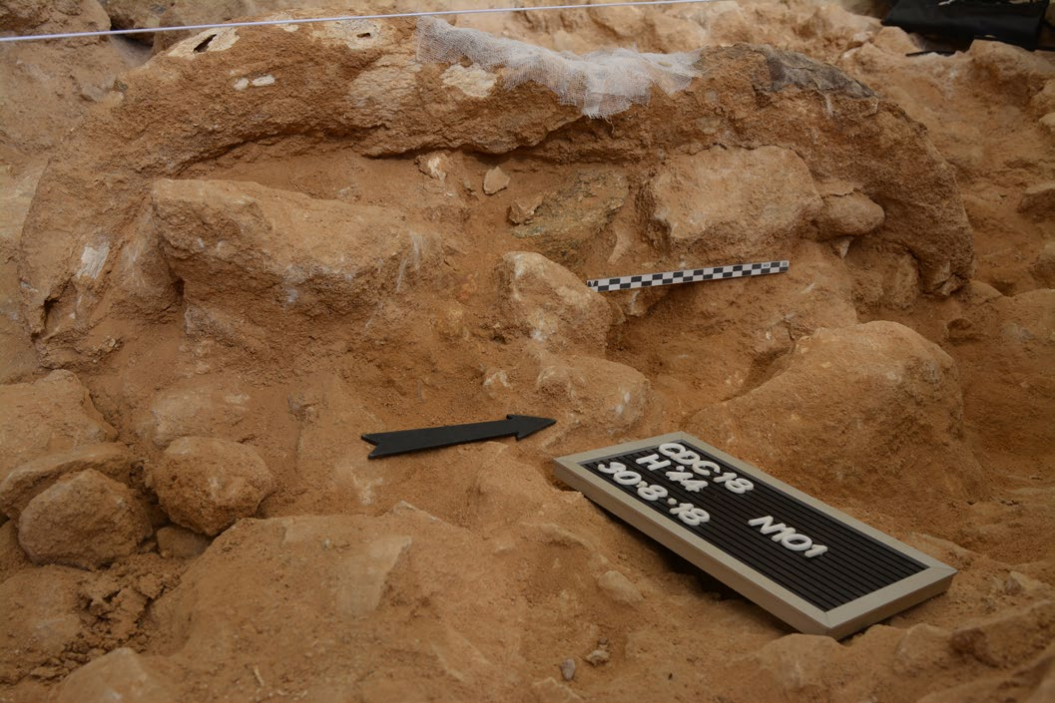
Gneiss anvil under an aurochs cranium. Detail of the excavation of Level 3 in square H′44, in which an aurochs (*Bos primigenius*) cranium (18/29/CDC/H′44/101/272) was identified. The black arrow points north. Under the cranium (above the scale bar), a tabular gneiss boulder was found. This is an allochthonous element for Level 3, which is composed of angular to subangular cobbles and boulders of limestone and dolostone, and a scanty carbonatic, silty matrix. It must have been brought into the cave by Neanderthals. Its poor preservation (its surface is altered, causing the disaggregation and loss of its mineral grains) allows no evidence of its use to be gleaned (see Supplementary Fig. 24 for a diagram highlighting the important elements in the picture).

**Fig. 2:**
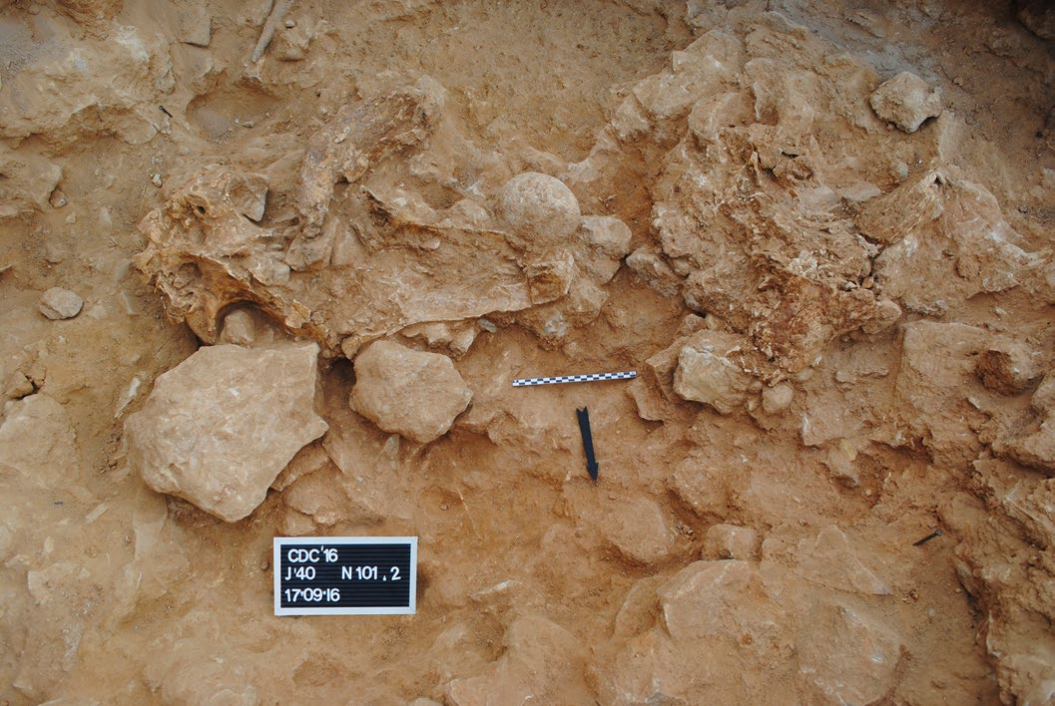
Steppe rhino cranium associated with a hammerstone. Detail of the process of excavation of Level 3 in square J′40, where a *Stephanorhinus hemitoechus* cranium (16/12/CDC/J′40/101.2/691) was found (face down). The photographed surface is approximately horizontal. The black arrow points north. The cranium lacks maxillae. Just above the cranium, there is a rounded granite boulder (16/12/CDC/J′40/101.2/600) with a battered area, indicating its use as a hammerstone. Note the bison horn core just above the rhino cranium and the remains of a large bovid cranium to the right (see Supplementary Fig. 25 for a diagram highlighting the important elements in the picture).

**Extended Data Fig. 4:**
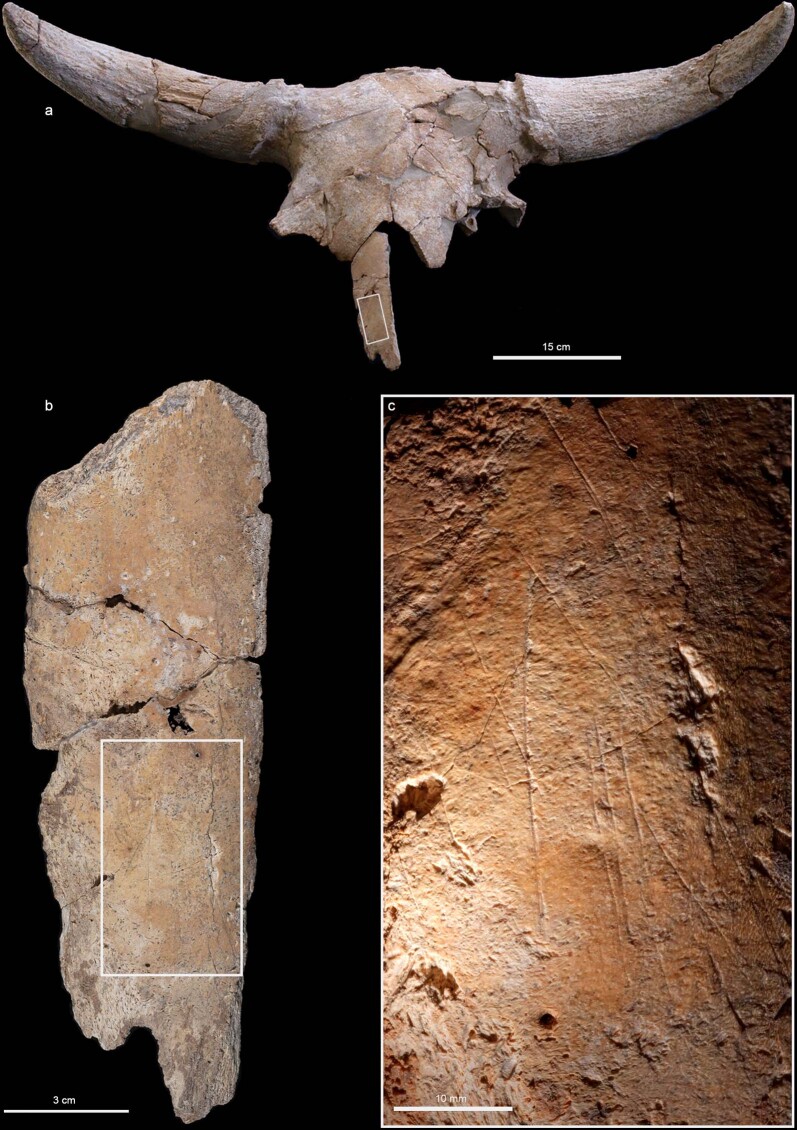
Cranium of *Bison priscus* (11/13/CDC/G’42/1/14) with cut marks on its nasal bone. a) General view of the cranium. The white rectangle indicates the area shown in Extended Data Fig. 4b. b) Detail of the left nasal bone. The white rectangle indicates the area shown in Extended Data Fig. 4c. c) Detail of the cut marks. Photo credit: Alfonso Dávila (b and c), Javier Trueba/MSF (a).

**Extended Data Fig. 5:**
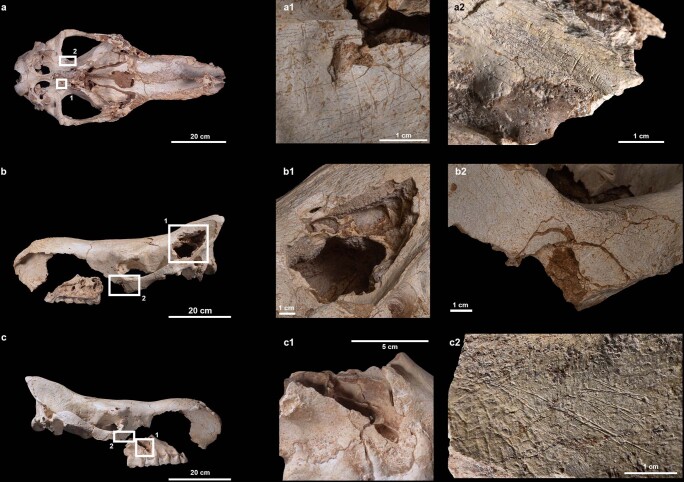
Cranium of *Stephanorhinus hemitoechus* (15/13/CDC/H’42/101/50) with anthropogenic modifications. a) Ventral view of the cranium showing anthropic modifications. a1) Detail of a percussion mark on the temporal bone. a2) Cut marks on the basisphenoid bone. b) Evidence of anthropic bone breakage on the left side of the cranium. b1) Detail of a broken parietal bone. b2) Detail of the broken zygomatic bone. c) Anthropic modifications on the right side of the cranium. c1) Detail of a fractured maxilla. c2) Cut marks on the zygomatic bone. Photo credit: Mario Torquemada/MAPCM (a, b, c and c2), Alfonso Dávila (a1, b1 and b2).

Overall, anthropic modification of the faunal remains was scarce (1.6%) and concentrated on post-cranial elements, especially the bones of the appendicular area (Supplementary Results, ‘Taphonomy’). The most common modification was fracturing (1.4%) by direct percussion to extract the bone marrow. Cut marks were found on just 0.3% of the studied bones. Four remains showed both types of modification. However, two crania, one belonging to *S. hemitoechus* and one to *B. priscus*, showed clear anthropic cut marks (Extended Data Figs. 4 and 5). Although post-depositional fragmentation made it difficult to study the evidence of anthropic fracturing, the former cranium showed signs of this as well as the noted cut marks. Indeed, some crania were spatially associated with anvils and hammers (Figs. 1 and 2).

**Fig. 3:**
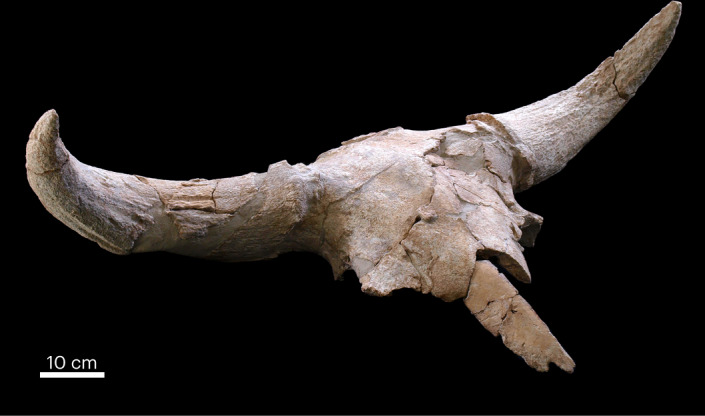
Steppe bison cranium from Level 3. This is one of the best-preserved *Bison priscus* crania from Level 3 (11/13/CDC/G′42/1/14). It shows the typical features of the set of the bison crania recovered at this level: the absence of zygomatic bones and maxillae, the preservation of the nasal and frontal bones, and horn cores. The nasal bone shows cut marks (Extended Data Fig. 4). Photo credit: Javier Trueba/MSF.

**Fig. 4:**
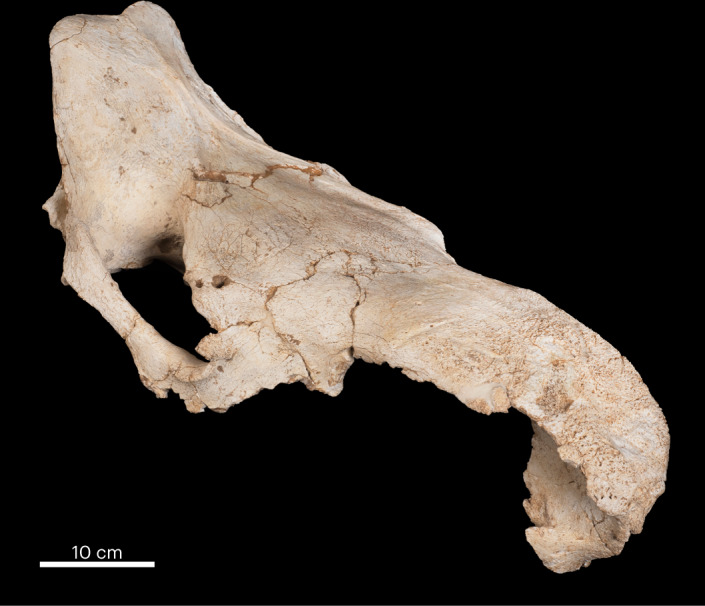
Steppe rhinoceros cranium from Level 3. Two *Stephanorhinus hemitoechus* crania were found in Level 3, both of similar integrity. Both lack maxillae (although the right maxilla of the cranium shown (15/13/CDC/H′42/101/50) was found underneath it). Numerous cut marks were identified on the zygomatic and basisphenoid bones of the pictured specimen, as well as evidence of anthropic fracturing (Extended Data Fig. 5). Photo credit: Mario Torquemada/MAPCM.

**Extended Data Fig. 6:**
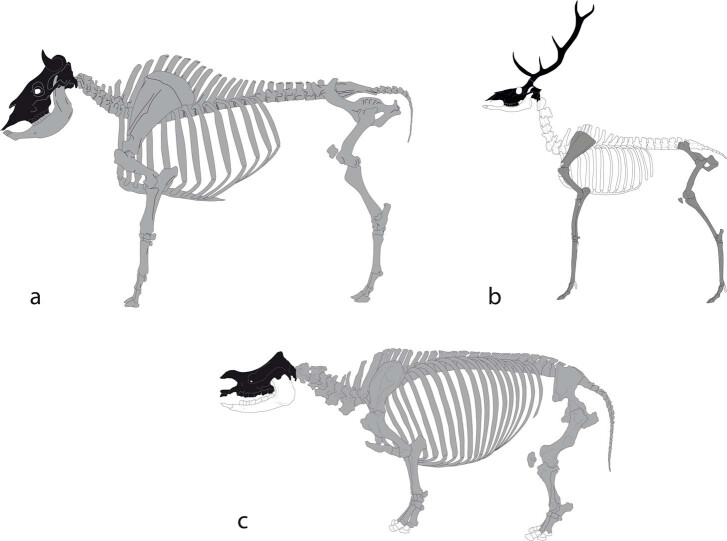
Skeletal representations of %MAU for the main taxa of Level 3. a) Large bovines (*Bison* and *Bos*); b) Red deer (*Cervus elaphus*); c) Steppe rhinoceroses (*Stephanorhinus hemitoechus*). Black indicates 100%; light grey < 5%; white 0%. The data from which these graphs were constructed can be found in Supplementary Tables 7, 8 and 9. Images adapted from ArcheoZoo.org. Creator credits: **a**, **c**: Michel Coutureau; **b**, Cédric Beauval and Michel Coutureau.

**Extended Data Fig. 7:**
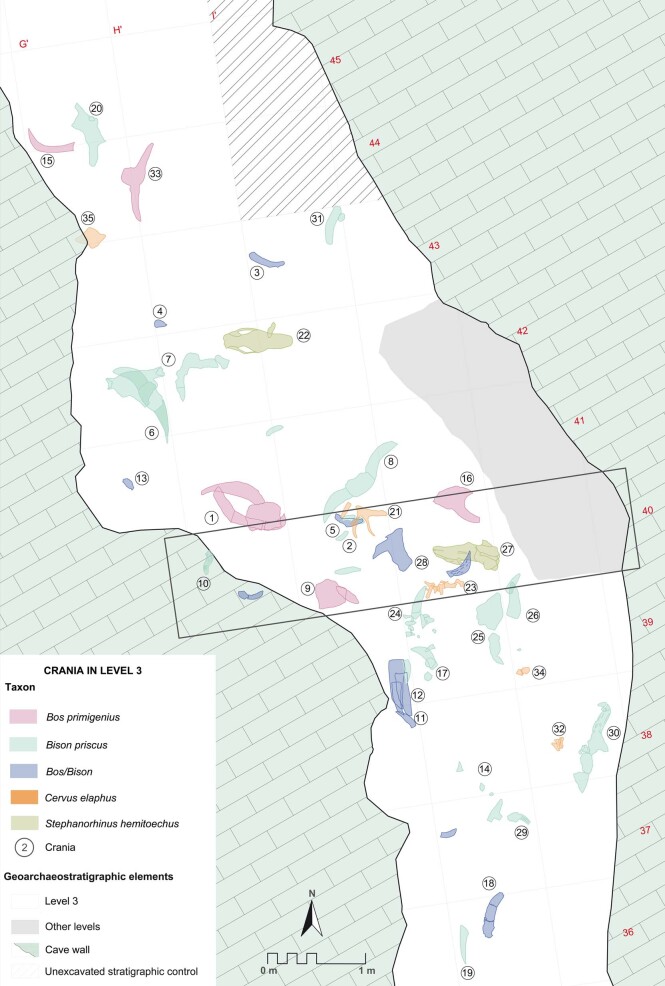
Plan of the “La Monumental” sector of the Cueva Des-Cubierta, showing the positions of the crania recovered in Level 3. Row 40 is highlighted with a rectangle since a cross-section is provided in Extended Data Fig. 9. The remains of 35 large mammals’ crania have been recovered from Level 3. The initials of the main fragments of each cranium are detailed in Supplementary Table 10. The circled numbers indicate the order of the crania in this table. This image, and Extended Data Fig. 9, compile the digitized field drawings for every square. The plan was generated with Autodesk AutoCAD 2021.

**Extended Data Fig. 8:**
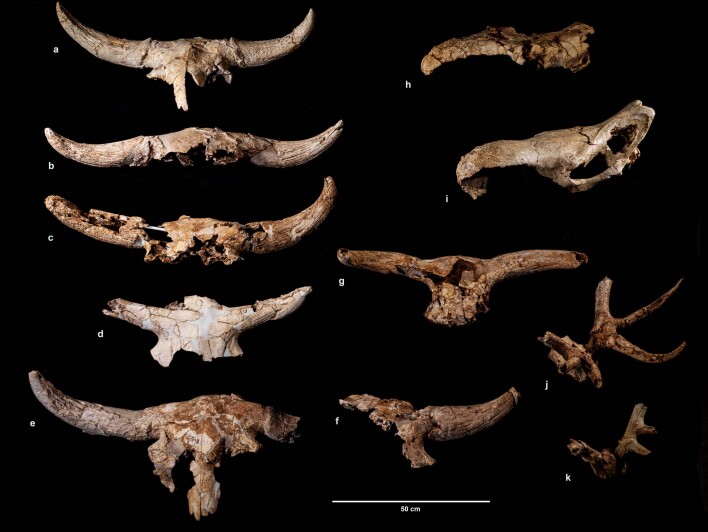
Some of the best preserved crania from the Cueva Des-Cubierta Level 3. a) A partial cranium of *Bison priscus* (6) with horn cores (plus a nasal bone); b) a partial cranium of *B. priscus* with horn cores (8); c) a partial cranium of *B. priscus* (25) with horn cores; d) a partial cranium of *B. priscus* (20) with horn cores; e) a partial cranium of *B. priscus* (7) with horn cores (including nasal bones); f) a partial cranium of *B. priscus* (24) with a horn core (24); g) a partial cranium of *Bos primigenius* (33) with horn cores; h) a partial cranium of *Stephanorhinus hemitoechus* (27); i) a partial cranium of *S. hemitoechus* (22); j) an antlered frontlet of *C. elaphus* (21); k) an antlered frontlet of *C. elaphus* (32). The numbers in parentheses indicate the order number of the crania in Supplementary Table 10. Photo credit: Javier Trueba/MSF.

The most notable feature of this large mammal association, however, is its anatomical composition (Extended Data Fig. 6 and Supplementary Fig. 15), which is clearly dominated by cranial remains, mostly crania with missing maxillae. Teeth are very scarce. All these crania belong to species with some form of appendage (bony horn cores, antlers or keratinous horns). *Equus ferus*, the only species with no cranial appendage belonging to the assemblage, is currently represented by only a tooth fragment and a metapodial. In total, the remains of 35 crania have been recovered, of which 28 belong to bovines (*B. priscus*, 14; *B. primigenius*, 3; *Bos/Bison*, 11), 5 to cervids (*C. elaphus*, 5; all males bearing their unshed antlers) and 2 to rhinoceroses (*S. hemitoechus*) (Extended Data Figs. 7 and 8). Many have suffered intense post-depositional fragmentation caused by the sediment that surrounded them. However, detailed analysis of the recovered fragments indicates that many of the crania (39.3%) initially conserved the frontal region, including any horn cores or antlers, as well as the occipital and nasal areas, but not the maxillae, the bony palate or the zygomatic bones (Figs. 3 and 4). Some crania were found lying over clusters of thermoaltered materials, including burned cranial fragments.

### Experimental butchering

The identification of anthropic marks on the crania and the under-representation of zygomatic bones, maxillae, mandibles and teeth suggest that the heads of these animals were first processed outside the cave. The scarcity of these elements has been interpreted in light of the results obtained in the experimental butchering of cow heads. Three cow heads were butchered, each by an experienced butcher, to identify which bones needed to be discarded or broken to extract the meat, brain and marrow of the heads (Supplementary Results, ‘Taphonomy’). When these cranial parts were removed through direct percussion, it was easy to extract the eyes (which are nutritive), but if these bones were not broken, the removal of these organs was extremely difficult. In addition, during the breakage of the maxillae, some upper teeth were accidentally extracted. If the first round of the butchering process had been performed inside the cave, fragments of mandibles, maxillae, zygomatic bones and/or upper and lower teeth would have been found. However, as indicated above, these elements are very scarce in Level 3. Thus, the initial butchering must have taken place outside the cave and was probably related to the consumption of the meat on the head, the tongue and eyes.

A second round of processing took place within the cave, perhaps related to accessing the brain and/or working the crania into the configuration in which they were found in Level 3. Although it is difficult to speak of a clear configuration for these crania, it is evident that a final round of their modification occurred in the cave. This is supported by the large number of percussion-associated tools present (~14% of all the lithic tools), by the percussion marks associated with the removal of the maxillae and the extraction of the brain (at least in the case of a rhinoceros cranium) (Extended Data Fig. 5), and by the presence of some isolated cranial fragments.

During the experimental butchering (Supplementary Results, ‘Taphonomy’), different options for accessing the brain were tested. The easiest method was to break the occipital bones. At the site, these bones are generally not complete and are sometimes isolated, suggesting that the Neanderthal occupants probably worked on the crania inside the cave to extract the brain.

## Discussion

The anatomical, taxonomic and taphonomic features of this association do not match what would be expected for a water- or gravity-driven accumulation^22,23^, a natural trap^24^ or a carnivore den^25–27^. Nor are they consistent with those resulting from the practice of subsistence activities by Neanderthals, such as hunting or the processing and consumption of their prey^5,24,28,29^. Although the high relative frequency of minimal animal units (%MAU) of crania might suggest the existence of a carnivore den, the anatomical and taxonomical features of the bone assemblage are not similar to those produced by carnivores, and certainly not by hyaenas^25–27^. Given the number of crania, another interpretation of the site might be that Level 3 was a kill-site. However, this can be ruled out given the absence of low-nutritional elements typically found at kill/butchering sites, such as flat bones^30–32^. Neither are there any parallels with the contexts interpreted for other, nearby sites in Pinilla del Valle. Certainly, for Level F of the Navalmaíllo Rock-shelter, where a Neanderthal hunting camp has been detected^5^, the skeletal profile matches those identified for other anthropogenic faunal accumulations of the Middle Palaeolithic. Other sites at Pinilla del Valle have been identified as hyaena dens (for example, at the Camino cave and Buena Pinta cave), for which the skeletal profiles are typical^33,34^. The faunal assemblage of the Cueva Des-Cubierta, however, is quite different and is not found elsewhere in the local archaeological record.

Studies involving modern hunter-gatherer groups have shown that the heads of large animals are usually discarded and not taken back to camp, since they are heavy and of lower use as food^35–40^. The introduction of the crania, and not of other parts of the carcasses of greater nutritional interest, into the Cueva Des-Cubierta thus seems to have been deliberate and not related to subsistence. Rather, it seems more related to their symbolic use.

To date, no site exclusively related to symbolic activity has been identified in the Neanderthal archaeological record. This is a limitation when trying to interpret the type of activity that the Neanderthals involved might have undertaken there: there is simply no comparative framework to help in this regard. Parallels with ethnographic examples might be useful in addressing this question.

Today, the accumulation and display of large mammal skulls in the form of hunting trophies is linked to sport hunting. Similar practices for varying purposes have, however, also been documented for the most recent hunter-gatherer societies^41^. Indeed, cultures worldwide have invested animal skulls with a strong symbolic content and have protected or displayed them with due attention^42^. The skulls of hunted animals were stored as souvenirs or hunting trophies among the Achuar people of South America^43^ and the Wola of New Guinea^44^. In other cultures, skulls (or other bones with symbolic meaning) were grouped together to form caches associated with hunting rituals. Different authors have understood these caches to be hunting shrines^41,45,46^. The display or accumulation of trophy skulls has also been linked to the construction of masculine identity (for example, among New Guinea lowland groups^47^) or the performance of specific ceremonies (for example, in the Ainu culture of northern Japan^48^). Other skull accumulations have been related to burial rituals (for example, among the Uilta people of Shakalin Island^49^).

In the present case, the fact that the crania all belong to species with cranial appendages (unshed antlers in the case of the deer) suggests that they may represent trophies. Their concentration in a small space also suggests that the accumulation might be considered a hunting shrine. However, other interpretations cannot be ruled out, such as a link with ritual and fire (given the proximity of the evidence of the latter’s use), some expression of the symbolic relationship between Neanderthals and the natural world, or some kind of initiatory rite or propitiatory magic.

**Extended Data Fig. 9:**
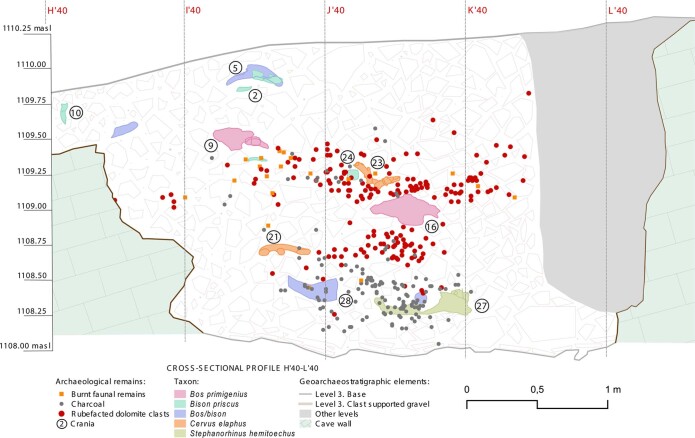
Cross-sectional profile H’40-L’40. The rectangle on the site plan in Extended Data Fig. 7 shows the location of the cross-sectional profile. Burnt faunal remains (orange squares), rubefacted dolomite clasts (red dots), charcoal (grey dots), and the recovered crania, can be seen throughout the depth of Level 3 (shown in meters above sea level). The presence of crania and concentrations of thermoaltered elements at different depths within Level 3 reflect a temporal order of events; the crania located at greater depths were introduced into the cave before those located at shallower depths, and the deeper clusters of thermoaltered elements are prior to shallower clusters. This is interpreted as evidence of the recurrent use of the cave by Neanderthals. The circled numbers indicate the order of the crania in the Supplementary Table 10. The profile was generated with QGIS 3.2 software.

The characteristics of the Cueva Des-Cubierta archaeological assemblage remain the same over the ~2 m thickness of Level 3 (Extended Data Fig. 9). The finding of crania, thermoaltered materials and lithic elements throughout, along with the continued presence of the tools necessary for that exploitation over the entirety of Level 3 (sometimes superimposed but separated from one another by packages of sediment), indicates that the site’s Neanderthal occupants repeated the same type of behaviour over a long period (years, decades, centuries or even millennia). The intentional deposition of large mammal crania over the time that Level 3 formed suggests the transmission of this behaviour between generations, which would be consistent with its interpretation as a cultural phenomenon.

Very few other accumulations of crania in Mousterian contexts are known. De Villeneuve^50^ described a Neanderthal accumulation of crania belonging to *Capra ibex*, *Bos primigenius* and *Cervus elaphus* in Level Foyer B of the Grotte du Prince (France), which was interpreted as a collection of hunting trophies. However, the absence of modern taphonomic studies on this assemblage requires that caution be used before this interpretation is fully accepted. Neanderthal burial sites have also been associated with deposits of large animal crania. Bonifay and Vandermeersch^51^ refer to a cranium and other bones of a cave bear in Le Regourdou Cave (France) as possibly reflecting a Neanderthal funerary offering, although this has been questioned by some authors^52–55^. Other possible offerings of crania at Neanderthal burial sites have also been reported (for example, in Teshik-Tash in Uzbekistan^56^). It is not until the arrival of anatomically modern humans, however, that the probable use of crania in ritual or symbolic contexts becomes more evident. For example, modern humans have been associated with the steppe bison cranium (interpreted as a possible hunting trophy with symbolic meaning^57^) found at the Régismont-le-Haut (France) site, with the cave bear cranium placed on a rock in the Grotte de Chauvet (France)^58,59^ and with the ochred steppe bison crania and jaws found at Anesovka II^60^. Large mammal crania have also appeared associated with modern human graves and have been interpreted as offerings—for example, the rhinoceros cranium at Brno 2 (Czech Republic)^61^ or the mammoth cranium associated with the ‘Red Lady’ grave in Paviland (Wales)^62^ (both in a Gravettian context). The accumulation of crania in the Cueva Des-Cubierta reported here provides further evidence of Neanderthal symbolism associated with the animals these humans hunted.

## Methods

Permission to conduct the excavations was granted by the Dirección General de Patrimonio Cultural of the Comunidad de Madrid.

### Field methods

When the excavation of the site began in 2009, a grid of 1 m^2^ squares was established. Each square was identified by a letter and a number. The letters grow towards the east, and the numbers grow towards the north.

The excavations were concentrated in the sector of the cave called La Monumental. The filling of the cave is well marked in terms of width by the walls of the cave. Given the need to know the cave’s complete stratigraphy, a trench with an approximate east–west direction began to be excavated in this sector in 2015. This trench occupies line 42 in the plan of the site (Extended Data Fig. 7). Excavations are still underway; the base of the fill has not yet been reached.

Excavation was carried out by archaeological level and, within each level, by artificial intervals (10 cm thick). The locations of the archaeological and palaeontological material and blocks of extracted rock were recorded with respect to an absolute coordinate system (datum ED50) using a motorized Leica total station (model TCRP 1205 R400). All data were exported to Excel 17.0 and then to a FileMaker Pro 7.0v3 database.

Stratigraphic levels were described, measured, logged using scaled drawings and photographed. Level limits were measured and recorded using a total station. Representative samples of each level from freshly scraped exposures were collected for granulometric analysis. Sieves (−1 to 4 *φ*) were used for measuring grain size distribution; sediment types were classified on the basis of this distribution^63^.

All the lithic industry detected was collected and drawn, regardless of size, as well as bone fragments over 2 cm (on the longest axis). All blocks of carbonatic rocks larger than 20 cm were also documented. All field drawings were made at a scale of 1:10 and digitized using a Wacom Intuos 4 professional pen tablet.

The coordinates of archaeological items were analysed spatially using AutoCAD 2021 and QGIS v.3.22. Different plans and sections were made from the spatial data to facilitate its visualization. These products were used to determine whether the distribution of the archaeological materials was homogeneous over the site’s extension or whether they appeared concentrated in specific sectors. The transversal and longitudinal sections allowed the vertical succession of the materials to be visualized and, taking into account the data derived from the geological and taphonomic studies, allowed temporal relationships between them to be established. The recovered objects were represented by their coordinates on the *x*, *y* and *z* axes in all plans and sections. The outlines of the large mammal crania were drawn in these plans and sections using information from photographs, field drawings and the spatial data.

Each recovered element was stored separately in a sealed plastic bag with its corresponding label containing information on the level, sublevel, square, order of recovery in that square, type of material, *z* coordinate (depth) and date. Larger fossils, also labelled, were stored in a container suitable for their characteristics. Only unidentifiable bones smaller than 2 cm from within the same square were kept together in a ‘level bag’; their coordinates were not recorded.

All excavated sediment was stored in bags labelled according to its origin (square stratigraphic level and depth range) for later pressurized water washing over a set of superimposed sieves.

### Human remains

The available mandible and isolated deciduous teeth were μCT-scanned to provide a virtual reconstruction of the mandible and to assess the developmental stages of the permanent dentition inside the mandibular corpus. All scanning was performed at the maximum resolution obtainable using a Phoenix v|tome|x s scanner (GE Measurement & Control) at the Centro Nacional de Investigación sobre la Evolución Humana (CENIEH) in Burgos, Spain. The isolated teeth were aligned along their long axis, with the crown placed upwards. Slices were then obtained in a 527 × 734 matrix in Dicom format, with an isometric voxel size of 0.019 mm (scanner energy, 110 kV; field of view, 0.9 cm). For the mandible, slices were obtained in a 1,880 × 1,176 matrix in Dicom format, with an isometric voxel size of 0.025 mm (scanner energy, 110 kV; field of view, 4.6 cm).

Virtual reconstruction was performed using Mimics v.18 software (Materialise), relying on semiautomatic segmentation to define Hounsfield values for dentine, enamel, bone and air. The virtual reconstruction of the mandible was performed taking into account the sagittal plane (symphysis) and mirror-imaging the preserved portions.

### Faunal remains

Reference collections and bibliographic sources were used for the identification of microvertebrate remains (rodents^64^, lagomorphs^65^, insectivores^66,67^, chiropterans^68–71^, amphibians^72–76^ and reptiles^77–79^). The systematics used in this work are those previously proposed for rodents^80,81^, insectivores^82^, chiropterans^83^, lagomorphs^80^, and amphibians and reptiles^84,85^.

Atlases of animal anatomy^86–89^ and additional comparative data from bibliographic sources^22,90–95^ were used for the anatomical and taxonomic identification of large mammal remains. For bovine remains, identification also involved the use of the criteria of the Natural History Museum of Rotterdam (the Netherlands) and the Department of Geology, University of Oviedo (Spain), as well as skulls of current *Bison bonasus* provided by the Palaeolithic Vivo Park (Salgüero de Juarros, Burgos, Spain). For the rhinoceros remains, other sources were consulted^96,97^. In-house data for modern *Panthera leo* collections and *Panthera spelaea* specimens (from the Museum of Vertebrate Zoology—UC Berkeley, the Smithsonian Institution, the British Museum, the Sociedad Aranzadi and the Institue für Quartarpalaontolgie Weimar) were used in comparative analyses.

Measurements were taken using a Mitutoyo digital caliper, recording to the nearest 0.05 mm. The osteological and dental terminology used, and the measurements made of teeth and bones, were those previously proposed^98^.

The data collected for each bone remain were anatomical element, taxon, size, position, age, portion and side^99^. Six measurements of abundance were recorded: the number of specimens (that is, the total number of faunal remains independent of their level of identification)^100^, the NISP, the MNE, the MAU, the standardized %MAU and the MNI^101,102^.

Non-identified specimens were included in three bone categories: long bones, flat bones and articular bones. Bones that showed structural features of ribs and vertebrae were classified as indeterminate flat bone/vertebra/rib. The non-identified specimens were classified into three size categories depending on the modified criteria of Rodríguez-Hidalgo^103^, who in turn modified the categories proposed by Brain^104^ and Bunn^105^ in their studies on extant African fauna and those of Díez Fernández Lomana^106^ (Supplementary Table 5).

To express the relative abundance between carnivores and ungulates (C/U ratio), the following ratio proposed by Thackeray^21^ was used:

$${{{\mathrm{C/U}}}}\;{{{\mathrm{ratio = }}}}\left( {{{{\mathrm{MNI}}}}\;{{{\mathrm{of}}}}\;{{{\mathrm{carnivores/ MNI}}}}\;{{{\mathrm{of}}}}\;{{{\mathrm{ungulates}}}}} \right) \times 100$$

In the present work, NISP values were used instead of the MNI values to calculate this index.

### Palynology

For the extraction of pollen grains from each of the studied samples, the sediments were chemically attacked using acids and alkalis according to standard protocols^107^. The residue was then subjected to enrichment by flotation in a heavy liquid of density ≥2. The residues were mounted on slides and examined under a light microscope. For each slide, pollen grains were counted along 42 rows covering the entire surface of the slide. Some of the samples from Levels 5 and 3 contained <100 grains; a pollen presence histogram was used to represent the results. Taxa were recorded as arboreal, shrubby and herbaceous types. The statistical treatment of the data, as well as their graphical representation, was undertaken using TILIA software^108,109^.

### Evidence of thermal alteration and hearths

A collection of different archaeological materials from the cave was examined macroscopically to search their surfaces for any signs of alteration by fire and to characterize these signs. A spatial distribution analysis of the studied materials was then performed using AutoCAD and QGIS software. The location and arrangement of remains affected by fire within the deposit facilitate and complement the identification of areas where combustion occurred^110,111^.

The dolomite clasts forming part of the sediment matrix of Levels 2 and 3 sometimes showed signs of having been affected thermally. These were identified and classified as previously reported^112^ for carbonated sedimentary rocks, taking into account changes in coloration, cracks and alterations caused by high temperatures.

Some lithic industry artefacts and remains showed signs of exposure to heat and fire, as determined by the presence of potlidding, cracking and flaking, loss of shininess (for quartz), colour changes, thermal lustre, and fractures (in flint). Several studies have reported common alterations to different raw materials caused by fire, including quartz^113^, while others have recorded the differences between naturally and intentionally heat-treated flint^114–116^.

Some of the bone remains also showed signs of thermal alteration. The main feature of burned bones is the change in colour related to the intensity of the fire and the time of exposure. The burned bones from this assemblage were clustered into five colour groups^117,118^: Grade 1, brown points dispersed across the bone surface; Grade 2, brown stains more or less homogeneous across the bone surface; Grade 3, black stains where the bone was charred; Grade 4, grey and white stains, although occasionally with bluish veins; and Grade 5, calcined and completely white.

Charcoal fragments were identified taking into account the specialized literature on their recognition via the internal structure of the fragments and the taphonomic processes undergone^119,120^.

### Taphonomy

The surfaces of all remains recovered were examined macroscopically and microscopically using an Olympus SZ1144TR (×15–×40) binocular microscope and a DigiMicro 2.0 Scale (×20–×200).

Cut marks were identified on the basis of previously proposed criteria^121–124^. The distribution, incidence and morphology of cut marks allows for the identification of different butchering activities^121^. Percussion pits, conchoidal scars, flakes and peeling caused by the anthropic breakage of the bones were also sought^125–127^. The presence of damage and its location were recorded for each of the remains analysed.

Carnivore modifications (tooth marks) were sought in the form of pits, scores and perforations^121,128^. The length and the width of these tooth marks and their location were recorded^129–132^. The presence of pitting and furrowing was also noted^121,133^.

Post-depositional modifications were noted in terms of presence/absence. Most of these modifications (black manganese oxide stains, dissolution, rounding and polishing by water abrasion, and concretions) are associated with the karstic depositional environment in which the materials were found^134–136^.

All faunal remains were subjected to classic taphonomic analysis. In addition, experimental butchering was performed to compare the results of the breakages made with the collected morphotypes. Three cow (*Bos taurus*) heads were butchered (one each by three experienced butchers) following different strategies to extract the brain. The butchers tried to identify the easiest way to extract the edible resources (eyes, brain and meat), always from a qualitative point of view (no quantitative data were collectable). In all cases, the butchers used stone tools (simple flakes) made from the raw materials (quartz, quartzite and porphyry) present in the lithic record for the site. All the raw materials were collected in the surroundings of Pinilla del Valle.

All experimental processes were undertaken at the Valle de los Neandertales Archaeological Park enclosure under natural conditions to reduce any possible bias produced by laboratory conditions (under which butchering is ergonomically easier).

The first stage involved the skinning of the heads. Most of the muscles (for example, the tongue) were then removed and the mandibles extracted (in agreement with the absence of these elements in the site) using gneiss, quartz and porphyry hammerstones. These raw materials were selected for use as percussion tools since most of the hammers found at the Pinilla del Valle sites (including the Cueva Des-Cubierta) are made from these kinds of rock. When the crania and mandibles were completely separated, the maxillae were removed. The zygomatic arches were then removed using hammerstones to extract the eyes.

Finally, the brain was extracted by (1) breaking the ventral part of the cranium, hitting it near the basilar part of the occipital bone; the brain was then extracted from within this area; (2) hitting the squamous part of the occipital bone (on both sides of the sagittal crest) with a hammerstone and then extracting the brain from within this area; or (3) hitting the frontal bone with a hammerstone and then extracting the brain from within this area. These three options were followed to check whether any provided an easier way to extract this organ.

### Radiocarbon dating

Several samples (charcoal and charred bones) from Levels 2 and 3 were sent to the Oxford Radiocarbon Accelerator Unit for radiocarbon dating by accelerator mass spectrometry using the ultrafiltration method. The chemical pre-treatment, target preparation and accelerator mass spectrometry measurement were as previously described^137,138^. Only one of the samples (OxA-31346, a charcoal fragment from Level 2) could be dated. The result was calibrated using OxCal v.4.4 software^139^ and the IntCal20 calibration curve^140^. The calibrated age was expressed in anni cal BP (95.4% confidence or 2*σ*). The dating results are available in the Supplementary Results under ‘Radiocarbon dating’.

### Uranium/thorium dating

The underlying speleothems S1 and S2 were sampled (using a hammer and chisel) for dating by the U/Th method to provide an estimate of the maximum age of Level 3. The S1 sample was divided into two. One part was sent to the Uranium Series Laboratory of the CENIEH, and the other to the Geochronology Laboratory of the Jaume Almera Institute of Earth Sciences (CSIC). S2 was dated at the CENIEH Uranium Series Laboratory.

The methodology and dating protocols used at the first of these laboratories have been previously described^141^. This facility works with small samples (around 50 mg); different subsamples taken from across the thickness of the speleothem could therefore be dated. The individualized datings obtained cover the interval of formation of the speleothem. The methodology and dating protocols used at the second laboratory are described elsewhere^142^. This laboratory works with bulk samples and therefore analysed the speleothem as a whole, obtaining an average age for the time interval during which it formed.

### Reporting summary

Further information on research design is available in the Nature Portfolio Reporting Summary linked to this article.

## Supplementary information

Supplementary Information Supplementary Figs. 1–46, Tables 1–31, Results and References.

Reporting Summary

## Data Availability

The archaeological and palaeontological remains reported here are deposited in the Museo Arqueológico y Paleontológico de la Comunidad de Madrid, Alcalá de Henares, Spain. The accession codes for the large mammal crania that are the main subject of this publication can be found in Supplementary Table 10. The data supporting the findings of this study can be found in the accompanying Supplementary Information.

## References

[1] Claud, E. et al. The practices used by the Neanderthals in the acquisition and exploitation of plant and animal resources and the function of the sites studied: summary and discussion. *Palethnologie* **10**, 350–467 (2019).

[2] Gabucio J, Fernández-Laso MC, Rosell J (2018). Turning a rock shelter into a home: Neanderthal use of space in Abric Romaní levels M and O. Hist. Biol..

[3] Marín J (2020). Neanderthal faunal exploitation and settlement dynamics at the Abri du Maras, level 5 (south-eastern France). Quat. Sci. Rev..

[4] Marín J (2019). Neanderthal logistic mobility during MIS3: zooarchaeological perspective of Abric Romaní level P (Spain). Quat. Sci. Rev..

[5] Moclán A (2021). A Neanderthal hunting camp in the central system of the Iberian Peninsula: a zooarchaeological and taphonomic analysis of the Navalmaíllo Rock Shelter (Pinilla del Valle, Spain). Quat. Sci. Rev..

[6] Romero AJ, Díez JC, Arceredillo D, García-Solano J, Jordá Pardo JF (2019). Neanderthal communities in the heart of the Iberian Peninsula: taphonomic and zooarchaeological study of the Mousterian site of Jarama VI (Guadalajara, Spain). Archaeol. Anthropol. Sci..

[7] Ortiz Nieto-Márquez I, Baena Preysler J (2017). Did stones speak about people? Flint catchment and Neanderthal behavior from Area 3 (Cañaveral, Madrid-Spain). Quat. Int..

[8] Balzeau A (2020). Pluridisciplinary evidence for burial for the La Ferrasie 8 Neandertal child. Sci. Rep..

[9] Pettitt, P. *The Palaeolithic Origins of Human Burial* (Routledge, 2011).

[10] Rendu W (2014). Evidence supporting an intentional Neandertal burial at La Chapelle-aux-Saints. Proc. Natl Acad. Sci. USA.

[11] Zilhão, J. in *Death Rituals, Social Order and the Archaeology of Immortality in the Ancient World: ‘**Death Shall Have No Dominion’* (eds Renfrew, C. et al.) 27–44 (Cambridge Univ. Press, 2015).

[12] Jaubert J (2016). Early Neanderthal constructions deep in Bruniquel Cave in southwestern France. Nature.

[13] Baquedano E (2012). Neandertales en el Valle del Lozoya: los yacimientos paleolíticos del Calvero de la Higuera (Pinilla del Valle, Madrid). Mainake.

[14] Baquedano, E., Márquez, B., Laplana, C., Arsuaga, J. L. & Pérez-González, A. in *Pleistocene and Holocene Hunter-Gatherers in Iberia and the Gibraltar Strait: The Current Archaeological Record* (ed. Sala Ramos, R.) 577–584 (Univ. de Burgos-Fundación Atapuerca, 2014).

[15] Baquedano, E. et al. The Des-Cubierta Cave (Pinilla del Valle, Comunidad de Madrid, Spain): a Neanderthal site with a likely funerary/ritualistic connection. In *Abstract Book 6th**Annual Meeting ESHE* 41 (European Society for the Study of Human Evolution, 2016).

[16] Rodríguez, L. et al. Neandertal remains from Pinilla del Valle (Madrid, Spain). In *Abstract Book 6th**Annual Meeting ESHE* 206 (European Society for the Study of Human Evolution, 2016).

[17] Liversidge HM, Molleson T (2004). Variation in crown and root formation and eruption of human deciduous teeth. Am. J. Phys. Anthropol..

[18] Bailey SE (2006). The evolution of non-metric dental variation in Europe. Mitt. Gesell. Urges..

[19] Martinón-Torres M, Bermúdez de Castro JM, Gómez-Robles A, Prado-Simón L, Arsuaga JL (2012). Morphological description and comparison of the dental remains from Atapuerca-Sima de los Huesos site (Spain). J. Hum. Evol..

[20] Alperson-Afil N, Richter D, Goren-Inbar N (2007). Phantom hearths and the use of fire at Gesher Benot Ya’aqov. Isr. PaleoAnthropol..

[21] Thackeray JF (1990). Carnivore activity at Klasies River Mouth: a response to Binford. Palaeont. Afr..

[22] Prat, F., Delpech, F., Cancel, N., Guadelli, J.-L. & Slott-Moller, R. Le bison des steppes, *Bison priscus* Bojanus, 1827, de la grotte d'Habarra à Arudy (Pyrénées-Atlantiques). *Paleo* **15**, 1–102 (2003).

[23] Castaños J, Castaños P, Murelaga X, Alonso-Olazabal A (2012). Kiputz IX: un conjunto singular de bisonte estepario (*Bison priscus* Bojanus, 1827) del Pleistoceno Superior de la Península Ibérica. Ameghiniana.

[24] Brugal, J.-P., Díez-Lomana, C., Huguet Pamiès, R., Michel, P. & Rosell Ardèvol, J. in *Paleolithic Zooarchaeology in Practice* (eds Haws, J. A. et al.) 1–12 (Archaeopress, 2006).

[25] Guadelli, J.-L. Étude taphonomique du repaire d'hyènes de Camiac (Gironde, France). *Bull. Assoc. Fr. Quat.* **26**, 91–100 (1989).

[26] Cruz-Uribe K (1991). Distinguishing hyena from hominid bone accumulations. J. Field Archaeol..

[27] Pickering T (2002). Reconsideration of criteria for differentiating faunal assemblages accumulated by hyenas and hominids. Int. J. Osteoarchaeol..

[28] Farizy, C., David, F. & Jaubert, J. Hommes et bisons du paléolithique moyen à Mauran (Haute-Garonne). *Gall. Préhist. Suppl.* **30**, 1–269 (1994).

[29] Jaubert, J. et al. Les chasseurs d'Aurochs de La Borde: un site du Paléolithique moyen (Livernon, Lot). *Doc. Archéol. Fr.* **27**, 1–157 (1990).

[30] Domínguez-Rodrigo M (2015). Another window to the subsistence of Middle Pleistocene hominins in Europe: a taphonomic study of Cuesta de la Bajada (Teruel, Spain). Quat. Sci. Rev..

[31] O’Connell JF, Hawkes K, Blurton-Jones N (1992). Patterns in the distribution, site structure and assemblage composition of Hadza kill-butchering sites. J. Archaeol. Sci..

[32] Voormolen, B. *Ancient Hunters, Modern Butchers: Schöningen 13II–4, Kill-Butchery Site Dating from the Northwest European Lower Palaeolithic* (Leiden Univ., 2008).

[33] Arsuaga JL (2012). Understanding the ancient habitats of the last-interglacial (late MIS 5) Neanderthals of central Iberia: palaeoenvironmental and taphonomic evidence from the Cueva del Camino (Spain) site. Quat. Int..

[34] Huguet R (2010). Homínidos y hienas en el Calvero de la Higuera (Pinilla del Valle, Madrid) durante el Pleistoceno Superior: resultados preliminares. Zona Arqueol..

[35] Binford, L. R. *Nunamiut Ethnoarchaeology* (Academic Press, 1978).

[36] Bunn HT, Bartram LE, Kroll EM (1988). Variability in the bone assemblage formation from Hadza hunting, scavenging, and carcass processing. J. Anthropol. Archaeol..

[37] O’Connell JF, Hawkes K, Blurton Jones N (1988). Hadza scavenging: implications for Plio/Pleistocene hominid subsistence. Curr. Anthropol..

[38] O’Connell JF, Hawkes K, Blurton Jones N (1990). Reanalysis of large mammal body part transport among the Hadza. J. Archaeol. Sci..

[39] Schoville BJ, Otárola-Castillo E (2014). A model of hunter-gatherer skeletal element transport: the effect of prey body size, carriers, and distance. J. Hum. Evol..

[40] Haynes G, Klimowicz J (2015). Recent elephant-carcass utilization as a basis for interpreting mammoth exploitation. Quat. Int..

[41] Russell, N. *Social Zooarchaeology: Humans and Animals in Prehistory* (Cambridge Univ. Press, 2012).

[42] Thildervisk, J. *Ritual Bones or Common Waste: A Study of Early Medieval Bone Deposits in Northern Europe* (Barkhuis & Univ. of Groningen Library, 2013).

[43] Descola, P. *In the Society of Nature: A Native Ecology in Amazonia* (Cambridge Univ. Press, 1994).

[44] Sillitoe P (2001). Hunting for conservation in the Papua New Guinea highlands. Ethnos.

[45] Frison, G. G. *Survival by Hunting: Prehistoric Human Predators and Animal Prey* (Univ. of California Press, 2004).

[46] O’Neill, C. C. *Bighorn Sheep Ritual in Northeast California: An Examination of the Loyalton Rockshelter Caches*. Master’s thesis, California State Univ. (2015).

[47] Rosman, A. & Rubel, P. G. *Feasting with Mine Enemy: Rank and Exchange among Northwest Coast Societies* (Columbia Univ. Press, 1971).

[48] Masuda R, Tamura T, Takahashi O (2006). Ancient DNA analysis of brown bear skulls from a ritual rock shelter of the Ainu culture at Bihue, central Hokkaido, Japan. Anthropol. Sci..

[49] Kirilova IV (2022). At the junction of ethnography, zoology and physics: new data on the bear cult on Sakhalin Island (Russian Far East). Sakhalin Mus. Newsl..

[50] De Villeneuve, L. in *Les Grottes de Grimaldi (Baoussé-Roussé)* (eds Boule, M. et al.) 1–156 (Imprimière de Monaco, 1906).

[51] Bonifay E, Vandermeersch B (1962). Dépôts rituels d’ossements d’ours dans le gisement moustérien du Regourdou (Montignac, Dordogne). C. R. Hebd. Acad. Sci..

[52] Gargett RH (1989). Grave shortcomings. Curr. Anthropol..

[53] Fosse, P., Morel, P. & Brugal, J.-P. in *L’**Ours et l’**Homme* (eds Tillet, T. & Binford, L. R.) 79–100 (ERAUL, 2003).

[54] Cavanhié, N. L’ours qui a vu l’homme? Étude archéozoologique et taphonomique du site paléolitique moyen de Regourdou (Montignac, Dordogne, France). *Paleo* **21**, 39–64 (2011).

[55] Pelletier M (2017). Rabbits in the grave! Consequences of bioturbation on the Neandertal “burial” at Regourdou (Montignac-sur-Vézère, Dordogne). J. Hum. Evol..

[56] Okladnikov, A. P. in *Teshik-Tash: Palaeolithic Man* (ed. Okladnikov, A. P.) 7–85 (Trudy Nauchno-Issledovatelskogo Instituta Antropologii, 1949).

[57] Brugal, J. P. Paléohistoire d’un crâne de bison aurignacien à Régismont-le-Haut (Hérault, France): de sa nature à sa valeur. *Paleo* **27**, 65–82 (2016).

[58] Clottes J (1995). L’originalité de la grotte Chauvet-Pont-d’Arc, à Vallon-Pont-d’Arc (Ardèche). C. R. Séances Acad. Inscr. B.

[59] Philippe, M. & Fosse, P. La faune de la grotte Chauvet (Vallon-Pont-d’Arc, Ardèche): présentation préliminaire paléontologique et taphonomique. *Paleo* **15**, 123–140 (2003).

[60] Stanko, V. N. in *Le Bison: Gibier et Moyen de Subsistance des Hommes du Paléolithique aux Paléoindiens des Grandes Plaines* (eds Brugal, J. P. et al.) 343–359 (APDCA, 1999).

[61] Oliva, M. *Palaeolithic and Mesolithic of the Czech Lands (Moravia and Bohemia) in the European Context* (Moravske Zemske Muzeum, 2017).

[62] Aldhouse-Green, S. H. R. *Paviland Cave and the ‘Red Lady’: A Definitive Report* (Western Academic and Specialist Press, 2000).

[63] Blott SJ, Pye K (2012). Particle size scales and classification of sediment types based on particle size distributions: review and recommended procedures. Sedimentology.

[64] Chaline, J. *Les rongeurs du Pléistocène Moyen et Supérieur de France*. (Centre National de la Recherche Scientifique, 1972).

[65] Chaline, J. in *Faunes et Flores Préhistoriques* (ed. Lavocat, R.) 397–442 (Boubée & Cie, 1966).

[66] Jammot, D. *Les Musaraignes (Soricidae, Insectivora) du Plio-Pléistocène d’**Europe: Considérations Générales sur les Soricidae, Évolution, Phylogénie, Classification* (Univ. de Dijon, 1997).

[67] Furió, M. *Los Insectívoros (Soricomorpha, Erinaceomorpha, Mammalia) del Neógeno Superior del Levante Ibérico* (Univ. Autònoma de Barcelona, 2007).

[68] Dupuis, I. *Les Chiroptères du Quaternaire en France* (Univ. de Paris I, 1986).

[69] Felten H, Helfricht A, Storch G (1973). Die Bestimmung der europäischen Fledermäuse nach der distalen Epiphyse des Humerus. Senckenb. Biol..

[70] Menu, H. & Popelard, J.-B. Utilisation des caractères dentaires pour la détermination des Vespertilionines de l’Ouest européen. *Le**Rhinolophe* **4**, 1–88 (1987).

[71] Sevilla P (1988). Estudio paleontológico de los Quirópteros del Cuaternario español. Paleontol. Evol..

[72] Sanchiz, F. B. in *Historia Biológica del Ferreret (*Baleaphrine muletensis*)* (eds Hemmer, H. & Alcover, J. A.) 61–108 (Editorial Moll, 1984).

[73] Bailon, S. *Amphibiens et Reptiles du Pliocène et du Quaternaire de France et d’Espagne: Mise en Place et Évolution des Faunes* (Univ. de Paris VII, 1991).

[74] Bailon, S. in *Fiches d’Ostéologie Animale pour l’Archéologie*, Série C (Varia), 1 (eds Desse, J. & Desse-Berset, N.) (Centre de Recherches Archéologiques du CNRS, 1999).

[75] Blain, H.-A. *Contribution de la Paléoherpétofaune (Amphibia & Squamata) à la Connaissance de l’Évolution du Climat et du Paysage du Pliocène Supérieur au Pléistocène Moyen d’Espagne* (Muséum National d’Histoire Naturelle de Paris, 2005).

[76] Blain H-A (2009). Contribution de la paléoherpétofaune (Amphibia & Squamata) à la connaissance de l’évolution du climat et du paysage du Pliocène supérieur au Pléistocène moyen d’Espagne. Treb. Mus. Geol. Barc..

[77] Barahona Quintana, F. F. *Osteología Craneal de Lacértidos de la Península Ibérica e Islas Canarias: Análisis Sistemático Filogenético* (Univ. Autónoma de Madrid, 1996).

[78] Barahona F, Barbadillo LJ (1997). Identification of some Iberian lacertids using skull characters. Rev. Esp. Herpetol..

[79] Szyndlar, Z. Fossil snakes from Poland. *Acta Zool. Cracov.* **28**, 3–156 (1984).

[80] Wilson, D. E., Lacher, T. E. Jr. & Mittermeier, R. A. (eds) *Handbook of the Mammals of the World: Lagomorphs and Rodents I* Vol. 6 (Lynx, 2016).

[81] Wilson, D. E., Lacher, T. E. Jr. & Mittermeier, R. A. (eds) *Handbook of the Mammals of the World: Rodents II* Vol. 7 (Lynx, 2017).

[82] Wilson, D. E. & Mittermeier, R. A. (eds) *Handbook of the Mammals of the World: Insectivores, Sloths and Colugos* Vol. 8 (Lynx, 2018).

[83] Wilson, D. E. & Mittermeier, R. A. (eds) *Handbook of the Mammals of the World: Bats* Vol. 9 (Lynx, 2019).

[84] Speybroeck J (2020). Species list of the European herpetofauna—2020 update by the Taxonomic Committee of the Societas Europaea Herpetologica. Amphib. Reptil..

[85] Sillero, N. et al. Updated distribution and biogeography of amphibians and reptiles of Europe. *Amphib. Reptil.* **35**, 1–31 (2014).

[86] Pales, L. & Lambert, C. *Atlas Ostéologique des Mammiféres* Vol. 1 (Centre National de la Recherche Scientifique, 1971).

[87] Schmid, E. *Atlas of Animal Bones: For Prehistorians, Archaeologists and Quaternary Geologists* (Elsevier Science, 1972).

[88] Pales, L. & Garcia, M. A. *Atlas Ostéologique pour Server à l’Identification des Mammiféres du Quaternaire* Vol. 2 (Centre National de la Recherche Scientifique, 1981).

[89] Barone, R. *Anatomie Comparée des Mammiféres Domestiques* Vols 1–2 (Vigot Fréges, 1999).

[90] Schertz E (1936). Zur unterscheidung von *Bison priscus* Boj. und *Bos primigenius* Boj. and metapodien un astragalus. Senckenbergiana.

[91] Bibikova VI (1958). Some distinguishing features in the bones of the genera *Bison* and *Bos*. Bull. Mosk. Obschtschestwa Isp Privoda NS Otdel Biol.

[92] Stampfli HR (1963). Wisent, *Bison bonasus* (Linné, 1758), Ur, *Bos primigenius* Bojanus, 1827, und Hausrind, *Bos taurus* (Linné, 1758). Acta Bern..

[93] Brugal JP (1985). Le *Bos primigenius* Bojanus, 1827 du Pléistocène moyen des grottes de Lunel-Viel (Hérault). Bull. Mus. Anthropol. Préhist. Monaco.

[94] Gee H (1993). The distinction between postcranial bones of *Bos primigenius* Bojanus, 1827 and *Bison priscus* Bojanus, 1827 from the British Pleistocene and the taxonomic status of *Bos* and *Bison*. J. Quat. Sci..

[95] Guadelli, J.-L. in *Le Bison: Gibier et Moyen de Subsistance des Hommes du Paléolithique aux Paléoindiens des Grandes Plaines* (eds Brugal, J.-P. et al.) 51–62 (APDCA, 1999).

[96] Guérin CLes (1980). Rhinóceros (Mammalia, Perissodactyla) du Miocéne terminal au Pléistocène Supérieur en Europe occidentale: comparaison avec les espèces actuelles. Doc. Lab. Géol. Fac. Sci. Lyon.

[97] Lacombat F (2005). Les rhinocéros fossiles des sites préhistoriques de l’Europe méediterranéenne et du Massif central: paléontologie et implications biochronologiques. BAR Int. Ser..

[98] von den Driesch, A. *A Guide to the Measurement of Animal Bones from Archaeological Sites*. (Peabody Museum Harvard Univ., 1976).

[99] Rodríguez-Hidalgo, A. et al. Human predatory behavior and the social implications of communal hunting based on evidence from TD10.2 bison bone bed at Gran Dolina (Atapuerca, Spain). *J. Hum. Evol.* **105**, 89–122 (2017). 10.1016/j.jhevol.2017.01.007 28366202

[100] Grayson, D. K. *Quantitative Zooarchaeology: Topics in the Analysis of Archaeological Fauna* (Academic Press, 1984).

[101] Binford, L. R. *Faunal Remains from Klasies River Mouth* (Academic Press, 1984).

[102] Lyman, R. L. *Vertebrate Taphonomy* (Cambridge Univ. Press, 1994).

[103] Rodríguez-Hidalgo, A. *Dinámicas Subsistenciales Durante el Pleistoceno Medio en la Sierra de Atapuerca: Los Conjuntos Arqueológicos de TD 10.1 y TD 10.2* (Univ. Rovira i Virgili, 2015).

[104] Brain, C. K. *The Hunters or the Hunted: An Introduction to African Cave Taphonomy* (Univ. of Chicago Press, 1981).

[105] Bunn, H. T. *Meat Eating and Human Evolution: Studies on the Diet and Subsistence Patterns of Plio-Pleistocene Hominids in East Africa* (Univ. of California, 1982).

[106] Díez Fernández Lomana, J. C. *Zooarqueología de Atapuerca (Burgos) e Implicaciones Tafonómicas del Estudio de Yacimientos del Pleistoceno Medio* (Univ. Complutense de Madrid, 1993).

[107] Coûteaux M (1977). À propos de l’interpretation des analyses polliniques de sédiments minéraux, principalement archéologiques. Suppl. Bull. Assoc. Fr. Quat..

[108] Grimm EC (1987). CONISS: a FORTRAN 77 program for stratigraphically constrained cluster analysis by the method of incremental sum of squares. Comput. Geosci..

[109] Grimm, E. C. *TGView* (Illinois State Museum, 2004).

[110] Sañudo Die P (2008). Spatial analysis of Bolomor Cave level IV (Tavernes de la Valldigna, Valencia). Ann. Univ. Stud. Ferrara Museol. Sci. Nat..

[111] Alperson-Afil N (2017). Spatial analysis of fire: archaeological approach to recognizing early fire. Curr. Anthropol..

[112] Soler, B. *Estudio de las Estructuras de Combustión Prehistóricas: Una Propuesta Experimental—Cova Negra (Xàtiva, Valencia), Ratlla del Bubo (Crevillent, Alicante) y Marolles-sur-Seine (Bassin Parisien, Francia)* (Univ. de Valencia, 2016).

[113] Driscoll K, Menuge J (2011). Recognising burnt vein quartz artefacts in archaeological assemblages. J. Archaeol. Sci..

[114] Clemente Conte I (1995). Sílex y lustre térmico en el paleolítico medio: ¿Alteración o técnica de talla? El ejemplo de Mediona I (Alt Penedès, Barcelona). Trab. Antropol. Etnol..

[115] Gibaja JF, Clemente Conte I (1997). El tratamiento térmico del sílex y sus repercusiones en la determinación de los rastros de uso: algunos ejemplos del neolítico en Cataluña. Rev. Arqueol. Pon..

[116] Agam, A. et al. Estimating temperatures of heated Lower Palaeolithic flint artefacts. *Nat. Hum. Behav.*10.1038/s41562-020-00955-z (2020). 10.1038/s41562-020-00955-z 33020589

[117] Stiner MC, Kuhn SL, Weiner S, Bar-Yosef O (1995). Differential burning, recrystallization, and fragmentation of archaeological bone. J. Archaeol. Sci..

[118] Cáceres, I. *Tafonomía de Yacimientos Antrópicos en Karst: Complejo Galería (Sierra de Atapuerca, Burgos), Vanguard Cave (Gibraltar) y Abric Romaní* (*Capellades, Barcelona)* (Univ. Rovira i Virgili, 2002).

[119] Allué E, Euba I, Solé A (2009). Charcoal taphonomy: the study of the cell structure and surface deformations of *Pinus sylvestris* type for the understanding of formation processes of archaeological charcoal assemblages. J. Taphon..

[120] Théry-Parisot I, Chabal L, Chrzavzez J (2010). Anthracology and taphonomy, from wood gathering to charcoal analysis: a review of the taphonomic processes modifying charcoal assemblages, in archaeological contexts. Palaeogeogr. Palaeoclimatol. Palaeoecol..

[121] Binford, L. R. *Bones: Ancient Men and Modern Myths* (Academic Press, 1981).

[122] Potts R, Shipman P (1981). Cutmarks made by stone tools on bones from Olduvai Gorge, Tanzania. Nature.

[123] Shipman P, Rose J (1983). Early hominid hunting, butchering and carcass-processing behaviors: approches to the fossil record. J. Anthropol. Archaeol..

[124] Domínguez-Rodrigo M, de Juana S, Galán AB, Rodríguez M (2009). A new protocol in differentiate trampling marks from butchery marks. J. Archaeol. Sci..

[125] Blumenschine RJ, Selvaggio MM (1988). Percussion marks on bone surfaces as a new diagnostic of hominid behaviour. Nature.

[126] Capaldo SD, Blumenschine RJ (1994). A quantitative diagnosis of notches made by hammerstone percussion and carnivore gnawing in bovid long bones. Am. Antiq..

[127] Pickering TR, Egeland CP (2006). Experimental patterns of hammerstone percussion damage on bones: implications for inferences of carcass processing by humans. J. Archaeol. Sci..

[128] Maguire JM, Pemberton D, Collett MH (1980). The Makapansgat limeworks grey breccia: hominids, hyaenas, hystricids or hillwash. Paleont. Afr..

[129] Selvaggio MM (1994). Carnivore tooth marks and stone tool butchery marks on scavenged bones: archaeological implications. J. Hum. Evol..

[130] Selvaggio MM, Wilder J (2001). Identifying the involvement of multiple carnivore taxa with archaeological bone assemblages. J. Archaeol. Sci..

[131] Domínguez-Rodrigo M, Piqueras A (2003). The use of tooth pits to identify carnivore taxa in toothmarked archaeofaunas and their relevance to reconstruct hominid carcass processing behaviours. J. Archaeol. Sci..

[132] Pickering TR, Domínguez-Rodrigo M, Egeland CP, Brain CK (2004). Beyond leopards: tooth marks and the contribution of multiple carnivore taxa to the accumulation of the Swartkrans Member 3 fossil assemblage. J. Hum. Evol..

[133] Haynes G (1983). A guide for differentiating mammalian carnivore taxa responsible for gnaw damage to herbivore limb bones. Paleobiology.

[134] Behrensmeyer, A. K. in *Palaeobiology: A Synthesis* (eds Briggs, D. E. G. & Crowther, P. R.) 232–235 (Blackwell Scientific, 1990).

[135] Coard R (1999). One bone, two bones, wet bones, dry bones: transport potentials under experimental conditions. J. Archaeol. Sci..

[136] Courty, M. A., Goldberg, P. & MacPhail, R. *Soils and Micromorphology in Archaeology* (Cambridge Univ. Press, 1989).

[137] Bronk-Ramsey C, Higham T, Leach P (2004). Towards high-precision AMS: progress and limitations. Radiocarbon.

[138] Bronk-Ramsey, C., Higham, T. F. G., Owen, D. C., Pike, A. W. G. & Hedges, R. E. M. Radiocarbon dates from the Oxford AMS system: archaeometry datelist 31. *Archaeometry*10.1111/j.1475-4754.2002.tb01101.x (2002).

[139] Bronk-Ramsey C (2009). Bayesian analysis of radiocarbon dates. Radiocarbon.

[140] Reimer PJ (2020). The IntCal20 Northern Hemisphere radiocarbon calibration curve (0–55 kcal BP). Radiocarbon.

[141] Alcaraz-Castaño M (2017). A context for the last Neandertals of Interior Iberia: Los Casares cave revisited. PLoS ONE.

[142] Ballesteros D (2017). New evidence of sea-level lowstands and paleoenvironment during MIS6 and 4 in the Cantabrian coastal karst: the Cobiheru cave (North Iberia). Earth Surf. Process. Landf..

