# Author Correction: A symbolic Neanderthal accumulation of large herbivore crania

**DOI:** 10.1038/s41562-023-01650-5

**Published:** 2023-06-19

**Authors:** Enrique Baquedano, Juan L. Arsuaga, Alfredo Pérez-González, César Laplana, Belén Márquez, Rosa Huguet, Sandra Gómez-Soler, Lucía Villaescusa, M. Ángeles Galindo-Pellicena, Laura Rodríguez, Rebeca García-González, M.-Cruz Ortega, David M. Martín-Perea, Ana I. Ortega, Lucía Hernández-Vivanco, Gonzalo Ruiz-Liso, Juan Gómez-Hernanz, José I. Alonso-Martín, Ana Abrunhosa, Abel Moclán, Ana I. Casado, Marina Vegara-Riquelme, Ana Álvarez-Fernández, Ángel C. Domínguez-García, Diego J. Álvarez-Lao, Nuria García, Paloma Sevilla, Hugues-Alexandre Blain, Blanca Ruiz-Zapata, M. José Gil-García, Adrián Álvarez-Vena, Teresa Sanz, Rolf Quam, Tom Higham

**Affiliations:** 1 Museo Arqueológico y Paleontológico de la Comunidad de Madrid, Alcalá de Henares, Spain; 2 Institute of Evolution in Africa, Madrid, Spain; 3 Department of Geodynamics, Stratigraphy and Palaeontology, Faculty of Geology, Complutense University of Madrid, Madrid, Spain; 4 UCM-ISCIII Research Centre for Human Evolution and Behaviour, Madrid, Spain; 5 IPHES-CERCA, Institut Català de Paleoecologia Humana i Evolució Social, Tarragona, Spain; 6 Departament d’Història i Història de l’Art, University Rovira i Virgili, Tarragona, Spain; 7 Unit associated with CSIC, Departamento de Paleobiología, Museo Nacional de Ciencias Naturales, Madrid, Spain; 8 Department of Geology, Geography and Environment, University of Alcalá, Alcalá de Henares, Spain; 9 University of Alcalá General Foundation, Alcalá de Henares, Spain; 10 Department of Biodiversity and Environmental Management, University of León, León, Spain; 11 Laboratory of Human Evolution, Faculty of Humanities and Communication, University of Burgos, Burgos, Spain; 12 Palaeobiology Department, National Natural Sciences Museum—CSIC, Madrid, Spain; 13 National Research Centre for Human Evolution (FA-CENIEH), Burgos, Spain; 14 Fundación Atapuerca, Ibeas de Juarros, Burgos, Spain; 15 Department History and Philosophy, Area of Prehistory, University of Alcalá, Alcalá de Henares, Spain; 16 Interdisciplinary Centre for Archaeology and Evolution of Human Behaviour, University of Algarve, Faro, Portugal; 17 Escuela de Posgrado, Universidad de Burgos, Burgos, Spain; 18 Physical Chemistry Department, Faculty of Chemical Sciences, Complutense University of Madrid, Madrid, Spain; 19 Institute of Geosciences (IGEO, UCM-CSIC), Madrid, Spain; 20 Geology Department, Oviedo University, Oviedo, Spain; 21 Department of Anthropology, Binghamton University (SUNY), Binghamton, NY USA; 22 Division of Anthropology, American Museum of Natural History, New York, NY USA; 23 Cátedra de Otoacústica Evolutiva y Paleoantropología (HM Hospitales-Universidad de Alcalá), Departamento de Ciencias de la Vida, Universidad de Alcalá, Madrid, Spain; 24 Oxford Radiocarbon Accelerator Unit, Research Laboratory for Archaeology and the History of Art, University of Oxford, Oxford, UK; 25 Department of Evolutionary Anthropology, University of Vienna, Vienna, Austria; 26 Human Evolution and Archaeological Sciences Forschungsverbund, University of Vienna, Vienna, Austria

**Keywords:** Archaeology, Anthropology, Cultural evolution, Archaeology

Correction to: *Nature Human Behaviour* 10.1038/s41562-022-01503-7. Published online 26 January 2023.

In the version of this article initially published, the first affiliation shown for Laura Rodríguez was incorrect and should have been listed as Department of Biodiversity and Environmental Management, University of León, León, Spain. The error has been corrected in the HTML and PDF versions of the article.

