# Supplementary material for: A symbolic Neanderthal accumulation of large herbivore crania
Source: Nat Hum Behav. 2023 Jan 26;7(3):342–52. doi: 10.1038/s41562-022-01503-7 (PMC10038806; doi:10.1038/s41562-022-01503-7)
Supplement: Supplementary file 1 — Supplementary Figs. 1–46, Tables 1–31, Results and References. [file 41562_2022_1503_MOESM1_ESM.pdf]

nature human behaviour

Article https://doi.org/10.1038/s41562-022-01503-7

A symbolic Neanderthal accumulation of large herbivore crania

In the format provided by the authors and unedited

Supplementary Information

Supplementary Results……………………………………………………………….…..2

The site……………….…………….………………………………..…………..….….2

Human remains……………..…………….……………………………..…...…15

Faunal remains…………………………………..……….……..…....26

Palynology……………………………………………………………….….....44

Lithics………………………………….…………………………………50

Evidences of fire…………………………………………………..………57

Taphonomy………………………..…………71

Radiocarbon dating……………………………………………..……………79

U/Th datings…………………………………………………..……….........81

Supplementary References………………………….……………………………..86

Supplementary Results

The site

Location and geographical framework

The Cueva Des-Cubierta cave (40º55'23"N 3º48'29"W, WGS84 datum; altitude 1112 m) is located in the upper valley of the Lozoya River (or Paular Valley), 70 km north of the city Madrid. This valley is a depression of about 255 km^2 limited by two NE-SW mountain alignments: the Montes Carpetanos mountains towards the northwest (with altitudes of over 2000 m, including the 'Pico de Peñalara' which reaches 2430 m, and the Altos del Hontanar mountains to the southeast (reaching some 1700 m). Both form part of the larger Sierra de Guadarrama range within the Spanish Central System, which runs through the centre of the Iberian Peninsula with an approximate W-E direction. The Lozoya River runs along the longitudinal axis of the valley from the southwest to the northeast. Its waters are today dammed by the Pinilla Dam, which holds back the water of a reservoir that supplies Madrid (Supplementary Figure 1). The area was designated a National Park in 2013.

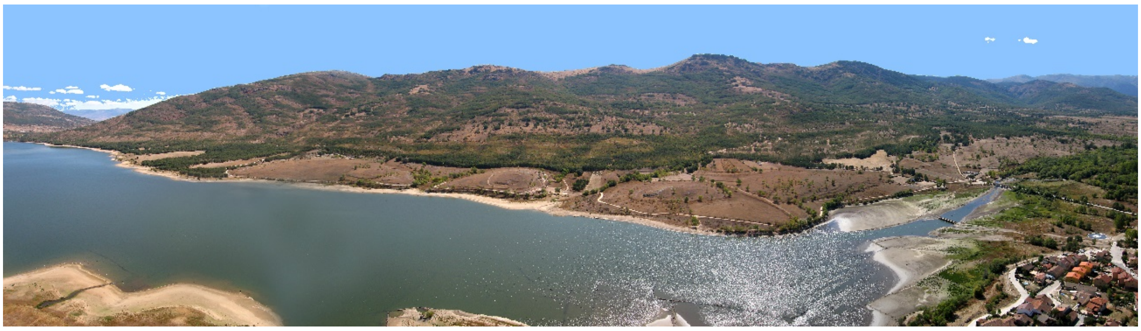

Supplementary Figure 1. Aerial view of the right bank of the Pinilla Reservoir, showing the Montes Carpetanos Range in the background, and the *calveros* of interest close to the edge of the reservoir. Pinilla del Valle village is in the right corner of the picture (modified from Baquedano *et al.*[143]; original version can be found at https://revistas.uned.es/index.php/ETFI/article/view/15604/13857).

The steep gradient of the territory - a 1350 m difference between the bottom of the valley and the surrounding peaks - produces a wide diversity of climatic and vegetational settings. At the bottom of the valley, the climate is of the continental Mediterranean type. In the nearby town of Lozoya (alt. 1093 m), the mean minimum temperature for the coldest month (December) is -3.1°C, while the mean maximum temperature for the

warmest month (July) is 28.3°C, and the average annual mean temperature is 10°C[144]. The mean annual rainfall is 703 mm, distributed throughout the year with two months of drought (July and August)[144]. In the highest areas, the climate acquires montane characteristics. In the nearby station of Puerto de Navacerrada (alt. 1890 m), the mean minimum temperature of the coldest month (February) is -3.9°C, the mean maximum temperature of the warmest month (July) is 20.9°C, and the average annual mean temperature is 6°C. The mean annual rainfall is 1423 mm, again distributed throughout the year with same two months of drought[144]. The bottom of the valley its home to a willow (*Salix* spp.) forest that follows the river bed, surrounded by ash trees (*Fraxinus angustifolia*) and Pyrenean oaks (*Quercus pyrenaica*) in areas with siliceous soil. Pyrenean oaks are replaced by gall oaks (*Quercus faginea*) in areas with calcareous soil. Pyrenean oak woods become dominant with rising altitude, until giving way to pine forests (*Pinus sylvestris* var. *iberica*), which in turn give way to high mountain scrubland (locally known as *piornales*) and juniper landscape. Silicophilous and psychroxerophilic grasslands are found in the areas with the highest peaks[144].

Background

The existence of cavities in the upper valley of the Lozoya River has been known of since at least the mid-19th century[145]. However, only in the last quarter of the 20th century was their archaeological and paleontological potential not realized, with the fortuitous discovery of the Cueva del Camino cave, then called the Pinilla del Valle site[146]. This site was excavated during the 1980s by researchers from Madrid's Complutense University, led by Prof. Francisco Alférez. The cave had been completely filled with sediments, but at the time of its discovery some of this had disappeared, a consequence of the cave roof falling in due to erosion, and to disturbance caused by construction of the country path that gives the cave its name (*camino* means path or track in Spanish). The first excavations provided one of the richest collections of vertebrate remains ever recovered in the Iberian Pleistocene, including two human teeth[146-155]. It was initially interpreted that the site formed during a warm period in the late Middle Pleistocene, and that the human teeth were identified as anteneanderthal. The recovery of stone tools and thermally altered bone remains led to the suggestion that the faunal remains had accumulated during the occupation of the cavity by an anteneanderthal group.

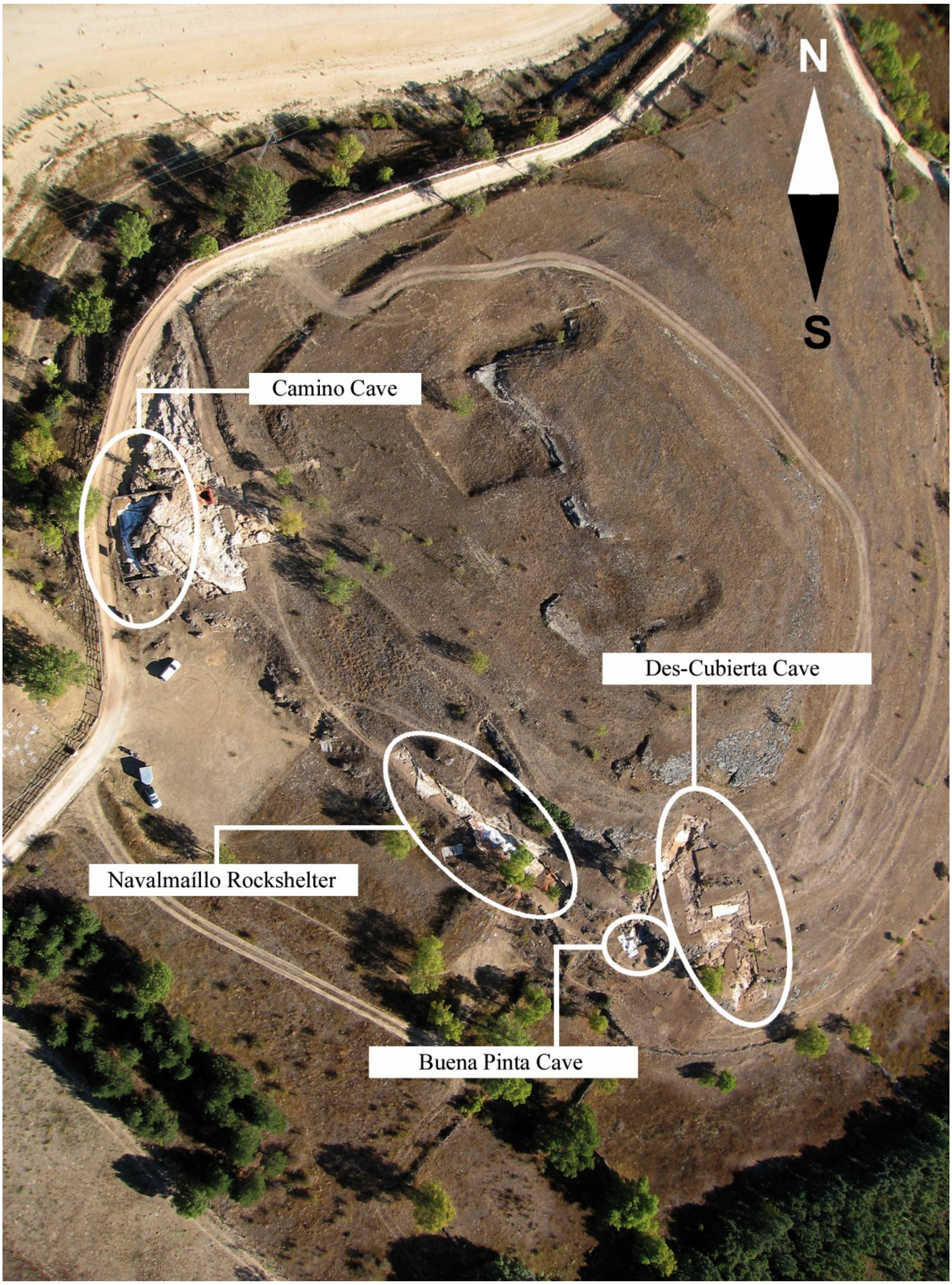

Supplementary Figure 2. Aerial view of the Calvero de la Higuera showing the location of the different archaeological sites (from Baquedano *et al.*[156]; original version can be found at https://arqueologiaprehistorica.es/arpi/#pdf-arpi-05/3/).

After a decade-long halt to excavation, a new team coordinated by the Archaeological and Paleontological Museum of the Community of Madrid returned in 2002. This team is currently led by Alfredo Pérez González, Juan Luis Arsuaga and Enrique Baquedano. Initially, this new work sought to clarify the taphonomic, chronological and environmental context of the cave, and to reappraise the palaeoanthropological remains.

New luminescence datings showed that the different fillings of the site formed from the end of MIS5 (*circa* 90 ka) until the MIS5/MIS4 transition (*circa* 74 ka)[33]. The vertebrate and pollen assemblages reflected the transition from a warm period (represented in the northern sector of the site) to a colder one (represented in its southern sector)[33,157,158]. The detailed study of the human molars revealed them to be Neanderthal[33], while the taphonomic analysis of the large vertebrate remains revealed a clear pattern of alterations produced by large carnivores, probably spotted hyenas (*Crocuta crocuta*). It was thus proposed that cavity was once a den used by these predators[33]; this agreed with an earlier interpretation made by other authors[159].

At the same time as the excavations of the Cueva del Camino cave were resumed, a survey was undertaken to look for new sites in the surrounding area. This led to the discoveries of the Abrigo de Navalmaíllo in 2002 and Cueva de la Buena Pinta cave in 2003[13] (Supplementary Figure 2). The first is a large rock shelter (over 300 m^2 in area) located about 100 m south of the Cueva del Camino cave, and has been shown to have hosted several Neanderthal occupations via the recovery of processed animal remains[34,160,161], concentrations of lithic tools[162-164] and the presence of hearths. Preliminary chronologies for these occupations range between 70-40 ka[165].

The Cueva de la Buena Pinta cave is located about 10 m south of the Abrigo de Navalmaíllo. At the time of its discovery in 2003 it was completely filled with sediment. In its outermost part, its roof had become dismantled by erosion, revealing its sedimentary infill. The site consists of a Holocene upper unit (level 1), under which lies a series (levels 2 to 5) of clayey and silty levels with a large number of fauna remains. The available dates place these levels in the middle of the Late Pleistocene (*circa* 60 ka)[165,166]. The concentration of large mammal remains was again interpreted as the work of spotted hyenas that used the cavity as a den[34]. Among these remains, two Neanderthal teeth were again found[16], to which a third was added in more recent campaigns must be added. The small mammal remains (including those of rodents and lagomorphs such as the root vole *Alexandromys oeconomus*, the narrow-headed vole *Lasiopodomys* cf. *gregalis*, and the

the steppe pika, *Ochotona* cf. *pusilla* [the only record of this species in the Iberian Peninsula[166]]) and the pollen record of the site indicate that its sedimentary series was deposited during a particularly cold period[166,168].

Discovery of the Cueva Des-Cubierta Cave

During the excavation of Cueva de la Buena Pinta cave, varying percentages of reworked Middle Pleistocene remains were identified among the assemblages of microvertebrates recovered from the Late Pleistocene levels[166,169]. The most evident corresponded to *Microtus brecciensis*, ancestor of *Microtus cabrerae*, and *Microtus vaufreyi*, two taxa typical of the Middle Pleistocene[170,171] - species that from an biochronological standpoint are incompatible with the main assemblage of small mammals for these levels. Indeed, the latter is dominated by *Microtus arvalis*, but includes other species characteristic of the Late Pleistocene, such as *Microtus cabrerae* and *Arvicola amphibius*. Further, the state of preservation of the remains of the chronologically discordant species was different to that of the other taxa. Specifically, the teeth of these species showed extensive manganese oxide impregnations that gave them a blackish colouration, contrasting with the paler tones of the remaining species[169].

The proportion of reworked remains in the Late Pleistocene levels of the Cueva de la Buena Pinta cave is, however, not constant, but varies over the stratigraphic series, with these elements becoming fewer towards the top, and increasing in number from the south towards the north[166]. This S-N gradient suggested that the sediments from which the reworked elements came might be found north of the excavated surface of the site, the limit of which was set in an erosive contact with Holocene materials (silts rich in organic matter).

Taking into account the possibility that the source sediments of the reworked materials might have been preserved, the Holocene sediments that limited the excavation surface to the north were removed in 2007, revealing a clast-supported breccia with dolomitic clasts and a sandy silt matrix with carbonatic cement. A detailed inspection of the matrix allowed the identification of several *Microtus vaufreyi* remains, and confirmed that these materials could have been the source of the reworked elements found in the Late Pleistocene sediments.

In 2009 a field campaign was organized to determine the extent of the *breccia* that contained the *Microtus vaufreyi* remains. For this, the edaphic deposits that smoothed out the slope of the hosting *calvero* (the local name for a small sloping hill) and which covered the geological substrate were removed, and it was confirmed that the breccia extended to the northeast along a narrow strip limited on both sides by parallel walls of Cretaceous dolomites. A few meters further, the *breccia* gave way to other silty and clayey sediments that were still confined on both sides by walls of Cretaceous dolomites. After a few meters more, the dolomite walls turned to the south, then to the east, and again to the south, all along defining a narrow strip (maximum width 4 m) filled with sediments of different nature (carbonatic breccias, chaotic accumulations of speleothems, clays, sands). The presence of speleothems attached to the dolomite walls revealed the entire conduit to be an old gallery of a cave filled with sediments, the roof of which had collapsed owing to erosion, leaving a roofless cave[172]. Subsequent excavations in a sector of these sedimentary fills led to the recovery of the materials described in the present work.

Geological context

The upper valley of the Lozoya River is of tectonic origin. It is a pop-down or sunken block limited by two pop-ups or raised blocks, the structuring of which took place during the Alpine Orogeny[173]. Reverse faults put the different blocks in contact[174]. The raised blocks are made of igneous or metamorphic siliceous rocks from the Hercynian basement. In the sunken block the sedimentary cover is preserved and includes materials deposited from the Late Cretaceous to the late stages of the Neogene. Quaternary deposits of different nature are recognized (terraces, moraines, peat bogs, alluvial fans)[175] lie over the materials of the raised and the sunken blocks. The Late Cretaceous sedimentary series begins with detrital deposits (sands, gravels and clays) of Turonian-Coniacian age[175]. Over these lie marls, limestones and dolomites of Late Cenomanian-Lower Turonian age[175]. Limestone and dolomite outcrops are mainly concentrated on the right bank of the Lozoya River, where they emerge in the form of *calveros* devoid of trees. These carbonatic rocks are extensively karstified[176], sometimes giving rise to large cavities such as the Cueva del Cabo del Río cave[177], and others that have been located underground following geophysical procedures[178,179].

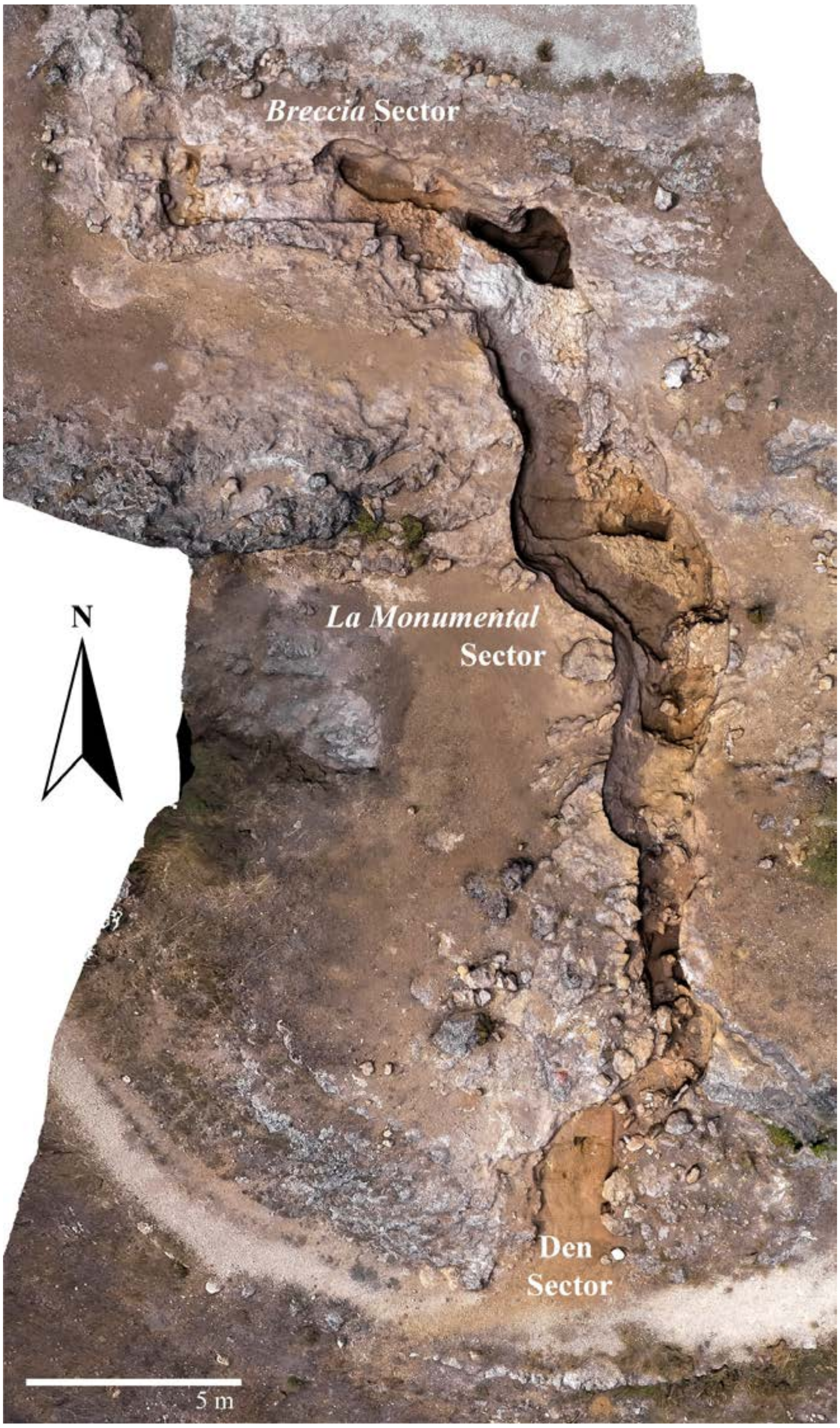

Supplementary Figure 3. Photogrametric restitution of the southern part of the Cueva Des-Cubierta cave. The layout of the old cave, now without a roof, can be seen. The different sectors into which the layout of the cave has been divided are indicated. Level 3 is located in the sector known as "La Monumental". Photo credit: Alfonso Dávila.

The Cueva Des-Cubierta cave is located in a *calvero* - known as the Calvero de la Higuera - on the right bank of the Lozoya River. This *calvero's* karst system develops in the 35 m thick Cretaceous limestones and dolomites that outcrop in the area. In these carbonate rocks, a multi-level cave system appeared, with sub-horizontal conduits, controlled by the structure and the nesting of the base level of the Lozoya River, which determined the evolution of the Quaternary landscape. Twenty four terrace levels have been identified in the Lozoya River valley: T1 (+ 220-205 m) to T15 (+ 62-64 m) belong to the Early Pleistocene, T16 (+ 50-55 m) to T20 (+ 17-20 m) to the Middle Pleistocene, T21 (+ 11-14 m) to T23 (+ 3-5 m) to the Late Pleistocene, and T24 (+ 1-2 m) to the Holocene, plus the floodplain[180].

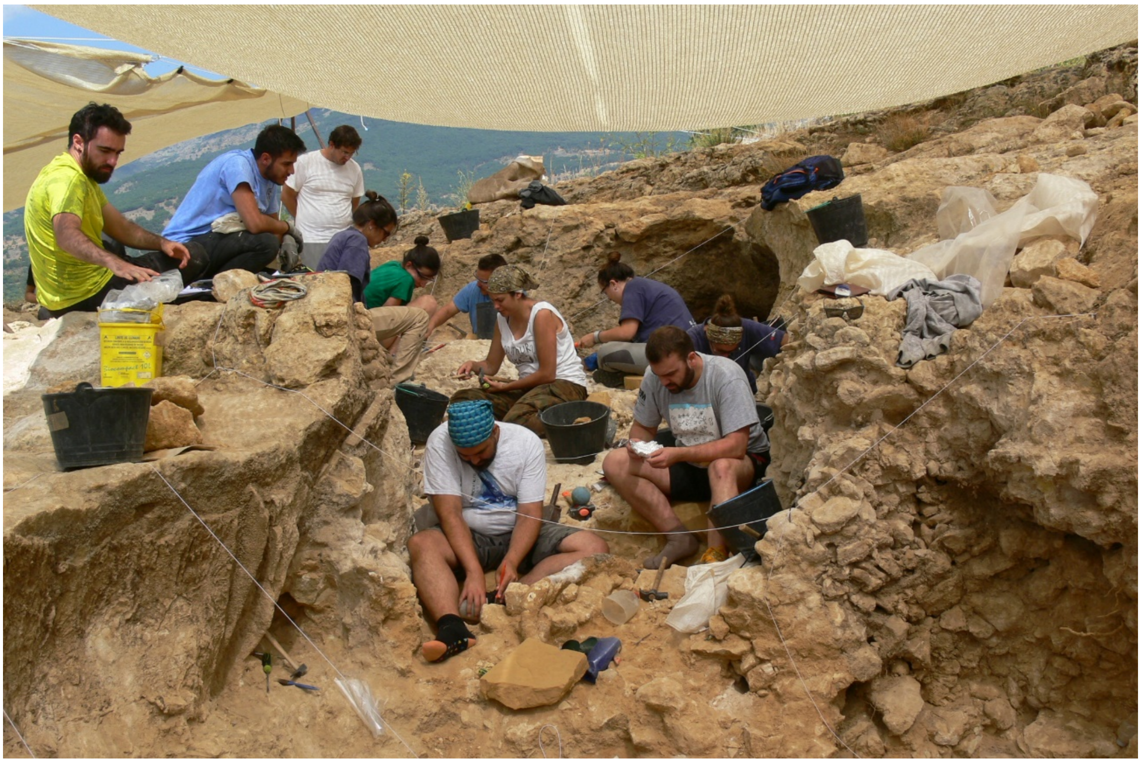

Supplementary Figure 4. Excavation in progress in the "La Monumental" sector of the Cueva Des-Cubierta cave during the 2018 campaign. One of the aurochs crania (18/29/CDC/H'44/101/272) in this level is being recovered (see Fig. 1 of the main text).

The Calvero de la Higuera multi-level karst lies between 1111 m (the top of the cave) and 1090 m above sea level (the cavities are at the edge of the reservoir). The Cueva Des-Cubierta cave lies in the uppermost known level. It is a roofless cave; its roof was dismantled by erosion at the surface of the Calvero de la Higuera (maximum altitude 1118 m) during the second half of the Late Pleistocene. Its name is a play on words, in Spanish

descubierta means both discovered and uncovered, and it alludes both to the fact that it was *discovered* by the current research team, and that it is an *uncovered* or roofless cave (Supplementary Figures 3 and 4).

It consists of a zigzag-shaped subhorizontal conduit under strong structural control, 80 m long by 2-4 m wide and about 3.5 m maximum (known) depth. It is located towards the southeastern boundary of the Calvero de la Higuera, and from its southern extreme it initially runs to the north. The first section is where the sector known as "La Monumental" is located (Supplementary Figure 3). It then zigzags to the west, then to the north, and finally to the southwest until reaching the Navalmaíllo Rockshelter and the Cueva de la Buena Pinta cave at lower altitudes.

Stratigraphy

The excavation of the "La Monumental" sector has not been completed nor has the bottom of the gallery been reached. The accessible stratigraphic series begins with Level 7 (Supplementary Figure 5), so far the oldest and deepest excavated unit of the sequence and which has only been exposed in the centremost, widest area of the cave. It consists of a light reddish-brown, sandy and silty clay with lenses of sands with scarce granules. The base of the unit has not been exposed, whereas the top of the unit is partially eroded.

A 10 to 15 cm thick speleothem (or flowstone) known as S1 precipitated over Level 7. The speleothem is complete and laterally continuous (although partially fractured in both the northern and southern sections) but gravitationally collapsed due to the erosion of the underlying Level 7 in the widest centremost part of the cave.

Level 6 is a light reddish brown sandy and silty clay deposited over the collapsed Level 7 speleothem fragments. Over an eastward dipping erosional surface a gour surface has formed due to the partial cementation of the clays.

Level 5 is a 30-45 cm thick light reddish brown sandy and silty clay with lenses of sands and granules, deposited over the above gour surface.

S2 is a thin (1-4 cm) speleothem precipitated over S1 in places where it is *in situ* and over Level 5 in areas where S1 has collapsed. S2 is overlain by the growth of several stalagmites in the centremost, widest areas of the cave.

Level 4 is a thin 15-20 cm clast-supported gravel deposit of flat, rectangular limestone and angular dolostone cobbles and boulders with a scant carbonatic clayey silt matrix.

Level 3 is a pale yellowish brown clast-supported gravel deposit, composed of limestone and angular to sub-angular dolostone cobbles and boulders with a scant carbonatic silty matrix. Towards the north and south of the cave, Level 3 rests conformably over S1 covering Level 7, whereas in the centre of the cave it lies on top of Level 4. Level 3 is up to 2 m thick in the centremost area and progressively becomes thinner outwards until it pinches out towards the North and South of the cave. In the centremost area of the cavity, the uppermost 30 cm appear edaphically modified.

Level 2 lies conformably over Level 3. Level 2 is a hardened pale reddish brown clast-supported gravel deposit cemented with carbonate, with rounded and sub-rounded heterometric limestone and dolostone pebbles and cobbles in an allochthonous silty sand matrix of calcareous, quartz, feldspar and mica composition alongside a few igneous rock fragments. Thin, laminated centimetric speleothem fragments are commonly found embedded in this level. Level 2 reaches its maximum thickness (55 cm) in the northernmost section, and pinches out southward.

Level 1 is an intensely cemented brown clast-supported gravel deposit with differently sized limestone and dolostone pebbles, cobbles and boulders, and with a silty sand carbonatic and quartz-feldspathic allochthonous matrix. It is up to 40 cm thick and liesconformably over Level 2, pinching out southward.

Level H is a 10-80 cm thick Holocene brown to dark brown Ap soil horizon up to 75 cm thick, unconformably overlying Levels 3, 2 and 1. This level includes taphonomically reworked fossils from these three underlying levels.

Site formation and chronology

The currently available data cannot confirm when the Cueva Des-Cubierta cave formed. Its origin is older than 500 ka since a stalagmite from the interior of the cavity has been dated to 513.4 ± 72 ka by the U-Th method. In addition, a molar of the rodent *Mimomys intermedius* (= *M. savini*, see[181], for a discussion on these taxa's synonymy) has been recovered from one of the stratigraphic levels in the southernmost sector of the site. This species is an important biochronological marker in Western Europe, having disappeared occurred more than 600 ka ago, i.e., before MIS15[182]. The presence of this species, even

as a reworked element, indicates that at some point there were sediments in the cavity that were deposited before the extinction of this species. These data suggest that the cavity already existed in the early Middle Pleistocene or late Early Pleistocene.

The U-Th dating of speleothem samples from different points of the Cueva Des-Cubierta cave outside of the La Monumental sector also suggests speleothems developed inside the cavity between MIS11 and the base of the MIS8.

In the La Monumental sector, Level 7 (comprised of reworked, water-laid sandy and clayey deposits) is so far the oldest deposit recorded. Since the oldest dating of the overlying S1 is 231 ± 7.6 ka (beginning of MIS7; Supplementary Tables 30 and 31), a minimum age of MIS8 is inferred for this unit. However, the base of S1 has not been dated and could be much older. S1 (which is 10 to 15 cm-thick) overlays the clayey and sandy deposits of Level 7. Five U-Th dates for the site place the formation of this speleothem as spanning the entirety of MIS7 (Supplementary Tables 30 and 31). Intense erosion, probably due to an important interglacial-glacial transition, affected the underlying Level 7 and partially wore S1 away. This led to the gravitational collapse of the speleothem in the wider cave areas.

Over these collapsed flowstone fragments, sandy and silty clay (Level 6) was deposited. These sediments were later eroded, and over this eastward dipping erosional surface, a gour surface developed due to the partial cementation of superficial clays. Overlying this gour surface, the sandy and silty clay of Level 5 is deposited. Small and large vertebrate remains confirm the karstic system was connected to the exterior, although the autochthonous nature of the sediments and fossil remains indicate this connection was either limited or far from this area.

S2 is precipitated over these deposits. Associated with this flowstone, several stalagmites developed. A $^{234}U/^{230}Th$ dating of one of these stalagmites gave an age of 135.7 ± 1.9 ka (Supplementary Tables 30 and 31) within MIS6a.

Conformably overlying the flowstone and stalagmites, flat, rectangular limestone and angular dolostone cobbles and boulders (Level 4) were deposited, probably due to the effect of frost weathering the walls and roof of the cave.

Level 3 was deposited over S2 and Level 4. The cobble and boulder morphology and the scant autochthonous carbonatic matrix reflect the initial stages of the cave roof’s collapse;

there is little or no allochthonous input. The pollen and micromammal assemblages suggest this level was deposited during a cold period. The maximum age of this level is defined by the dating of the underlying S2, which places it at the end of MIS6. An age for Level 3 within MIS5 is unlikely because conditions were predominantly warm during this interval, while the pollen and micro mammal records indicate cold conditions. Level 3 was therefore probably deposited during MIS4 or the earlier half of MIS3, at the end of which the Neanderthals became extinct. A Neanderthal connection with Level 3 is clearly documented by the typically Mousterian industry recovered.

Level 2 lies conformably over Level 3. Rounded and sub-rounded heterometric pebbles and cobbles lying alongside a silty sand matrix with quartz, feldspar, mica and igneous rock fragments, suggest there were already openings in the cave allowing for allochthonous input. A charcoal fragment with a calibrated date (2σ) of 43,402-52,964 cal BP (84.6%) and 53,154 cal BP-out of range (10.9%) indicates a minimum age of around the middle of MIS3. The mild and humid conditions implied by the pollen embedded in Level 2 indicates it was deposited during a warm oscillation during the predominantly cold MIS3 or MIS4.

Level 1 lies conformably over Level 2, and is interpreted as a continuation of the denudation and erosion of the cave, with important allochthonous input. Level H represents a Holocene soil unconformably overlying Levels 3, 2 and 1.

Supplementary Figure 5. Stratigraphic sections of the "La Monumental" sector of the Cueva Des-Cubierta cave. Profiles from different grid-squares are ordered from south (left) to north (right). The position of each profile is indicated on the site plan at the bottom right of the figure. Dashed lines indicate stratigraphic correlations between different profiles. Arrows point to the provenience of the dated samples. All the reported dating uncertainties are given at the 2σ level (OOR = out of range).

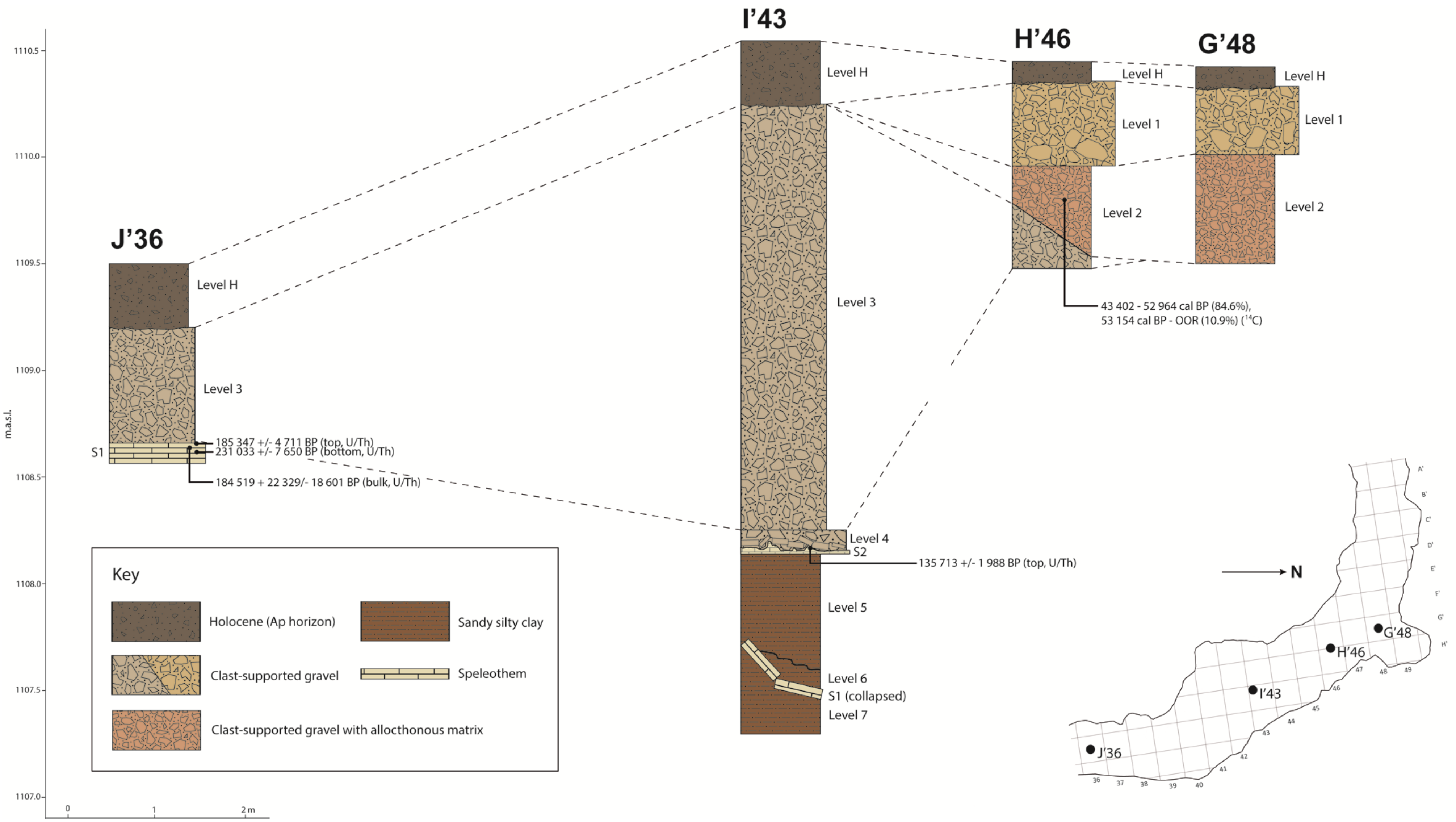

J'36
I'43
H'46
G'48
Level H
Level 1
Level 2
Level 3
Level 4
S2
Level 5
Level 6
S1 (collapsed)
Level 7
S1
185 347 +/- 4 711 BP (top, U/Th)
231 033 +/- 7 650 BP (bottom, U/Th)
184 519 + 22 329/- 18 601 BP (bulk, U/Th)
135 713 +/- 1 988 BP (top, U/Th)
43 402 - 52 964 cal BP (84.6%),
53 154 cal BP - OOR (10.9%) (^{14}C)
Key
Holocene (Ap horizon)
Sandy silty clay
Clast-supported gravel
Speleothem
Clast-supported gravel with allocthonous matrix
m.a.s.l.
1110.5
1110.0
1109.5
1109.0
1108.5
1108.0
1107.5
1107.0
0
1
2 m
N

Human remains

Some human remains have been identified in the Cueva Des-Cubierta cave. The lack of any repeated element, and the inferred ontogenetic age of all the remains being the same, is compatible with their belonging to a single individual. Five upper deciduous teeth were recovered *in situ* in the cemented sediments of Level 2, squares G'47 and H'47. Two jaw fragments that refit each other were recovered from Level H (Holocene) near the contact with the underlying Pleistocene sediments in square G'41. In that square, Level H rests on Level 3. These mandibular remains were likely moved from their original position in Level 2, which outcrops a few meters to the north, by post-depositional processes. Finally, a deciduous lower canine was recovered in Level 3 in the adjacent square H'41, one meter below one of the mandibular fragments. This element fits into the corresponding alveolus of the aforementioned mandible. After its detachment from the jaw it probably reached its derived position due to percolation through the gaps between the blocks of Level 3.

Preservation of the Cueva Des-Cubierta 1 individual

The Cueva Des-Cubierta 1 mandible is comprised of two fragments preserving the corpus from the right di2 to the left dm2 (Supplementary Figure 6). The roots of the left deciduous molars, canine and lateral incisor are partially preserved in the alveolar sockets, and the partial crown of the heavily eroded left dc1 is also preserved. Moreover, 6 isolated deciduous teeth have been recovered: 5 deciduous upper teeth (from left side dM1 to dI2 and one dI1 and one dM2 from the right side) and a lower right deciduous canine.

Description of the mandible

Virtual reconstruction and mirror-imaging of the Des-Cubierta mandible allows us to confirm that it has a squared-shaped dental arcade (Supplementary Figure 7). The external symphysis of the Des-Cubierta mandible is retreating and lacks the features related to the presence of a bony chin. The angle of the anterior symphysis in the Des-Cubierta mandible is low, whether this is measured relative to the alveolar plane (67.6°) or the basal plane (81.0°). Internally, there is a well-developed *alveolar planum* and the genioglossal fossa is triangular in shape and deep. A virtual cross-section at the level of the symphysis (Supplementary Figure 7) reveals a thickening of the cortical bone on the internal aspect, corresponding to the *alveolar planum*. The digastric fossae on the inferior

aspect of the symphysis face inferiorly and posteriorly and are separated by an extension of the mental spine. As in Gibraltar 2 and Archi 1[183], the Des-Cubierta 1 mandible is gently arching along the inferior border of the symphysis in anterior view.

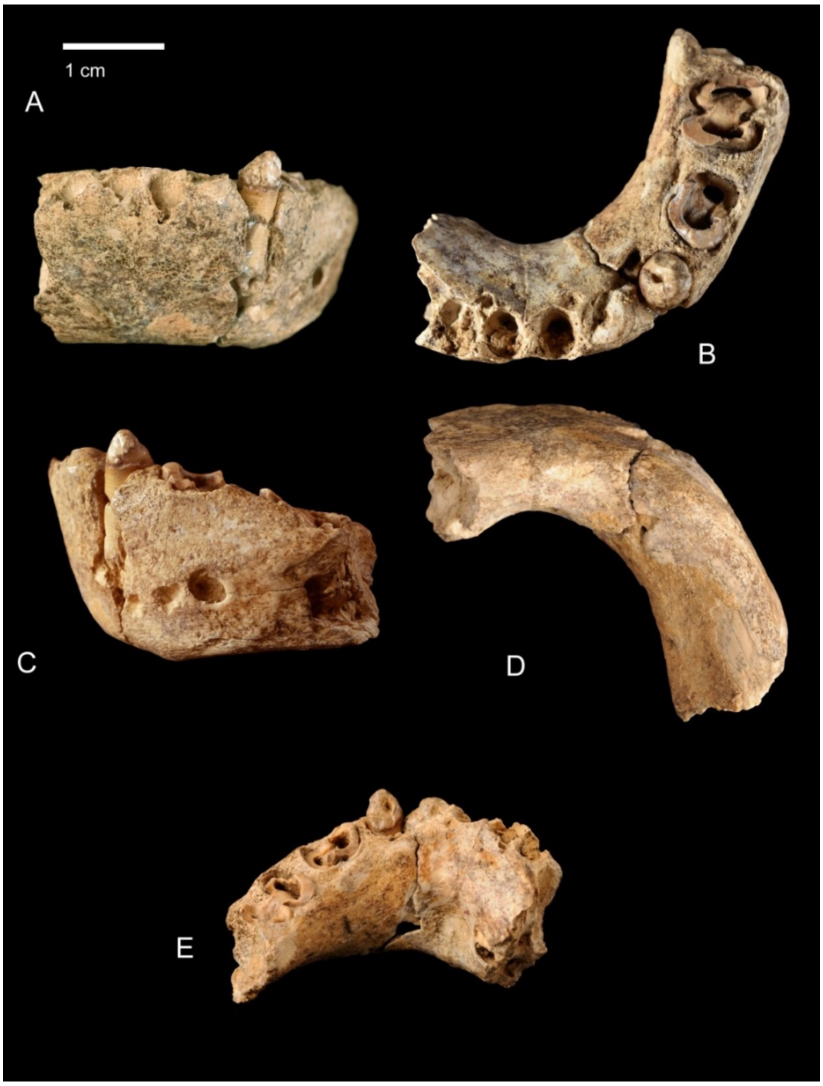

Supplementary Figure 6. The Cueva Des-Cubierta mandible (12/9/CDC/G'41/1) and 12/9/CDC/G'41/3) in anterior (A), superior (B), left lateral (C), inferior (D) and posterior (E) views. Photo credit: Mario Torquemada/MAPCM.

On the external face of the lateral corpus, a single, large mental foramen is located under the dm_1/dm_2 septum and is placed in the lower half of the body. Neandertals generally show a more posteriorly placed mental foramen with respect to the tooth row, even in the youngest individuals[184,185]. A similar placement below the dm_1/dm_2 is seen in 37.5% of Neandertals of a similar developmental stage as Des-Cubierta 1, while this placement in

modern humans is much less frequent (7.4%)[184]. In addition, the mental foramen is invariably placed in the lower half of the mandibular corpus in adult Neandertal mandibles, and this appears to be a derived feature in this taxon[186]. Internally, the mylohyoid line is pronounced and slightly inclined.

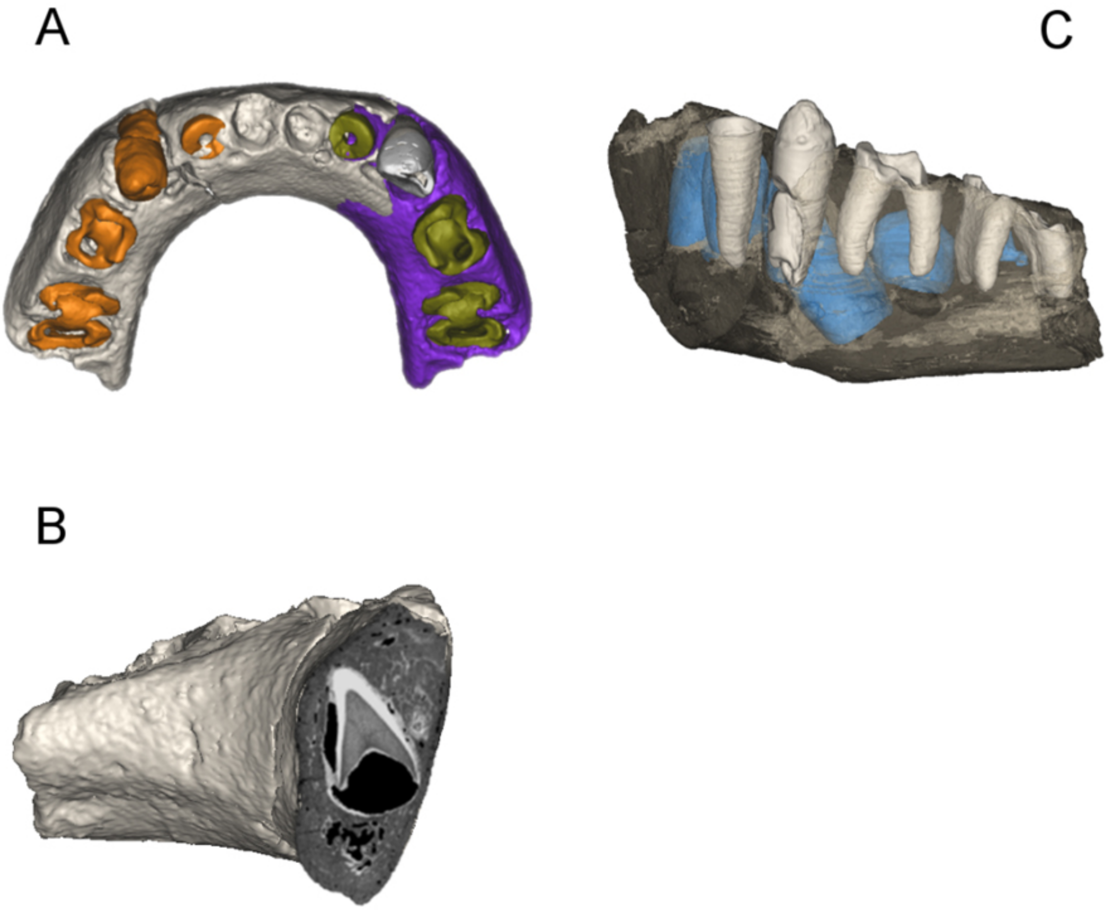

Supplementary Figure 7. Virtual reconstruction and mirror-imaging of Des-Cubierta 1. A) Virtual reconstruction showing the square shape of the alveolar arcade (grey = original specimen; purple = mirror-imaged right side; orange = original teeth; green = mirror-imaged teeth). B) Cross-section of the symphysis showing thickened cortical bone and a retreating symphysis with no chin structures. The developing germ of the first permanent incisor is also seen in the cross-section. C) Semitransparent lateral view of Des-Cubierta 1 mandible showing the root formation of the deciduous teeth and the developing permanent dentition.

The corpus height (16.4 mm) and thickness (11.8 mm) can be measured at the mental foramen. The resulting robusticity index in Des-Cubierta 1 (72.3) falls toward the upper end of the Neandertal range of variation, close to that of Roc de Marsal (Supplementary

Table 1) and Barakai. The value in Des-Cubierta 1 falls 1.68 s.d. above the mean of a small recent human sample and is closest to Skhul 1 among the fossil *H. sapiens* specimens. Comparison of fossil and recent juvenile mandibles shows Neandertals to be generally characterized by proportionally thicker mandibles for their developmental age[185].

Supplementary Table 1. Corpus measurements at the mental foramen for Des-Cubierta 1, Neandertals and modern humans. Modified from Quam *et al.*[185].

Specimen/Sample	Age at death (yrs.)[a]	Corpus height at mental foramen (mm)	Corpus thickness at mental foramen (mm)	Robusticity Index at mental foramen	Source
Des-Cubierta 1	3-5	16.4	11.8	72.0	This study
Neandertals					
Palomas 49	c. 2.0	19.2	11.4	59.4	[187]
Barakai	c. 3.0	20.1	14.2	70.6	[188]
Archi 1	c. 3.0	20.0	12.0	60.0	[188]
Roc de Marsal	c. 3.0	17.0	12.7	74.7	[189]
Devil's Tower	c 3.1	22.8	13.6	59.6	[188], age from [190]
Il Molare 1	c. 3.5	20.9	12.2	58.4	[191]
Palomas 7	c. 4.0	21.3	12.7	59.6	[187]
La Chaise 13	c. 4.0	20.5	12.5	61.0	[188]
Gegant-5	4.5–5.0	22.6	12.8	56.6	[185]
Cova Negra (CN 7755)	c. 5.0	20.0	13.3	66.5	[192]
Combe Grenal 1	c. 7.0	27.4	13.6	49.6	[193]
Modern Humans					
Le Figuier	c. 3.0	18.0	10.7	59.4	[194]
La Madeleine 4	c. 3.0	19.0	9.4	49.5	[195]
Lagar Velho	c. 4.5	20.5	11.5	56.1	[196]
Skhul 1	c. 4.5	16.4	11.0	67.1	[188]
Qafzeh 4	c. 6.0	26.3	14.2	54.0	[185]
Qafzeh 10	c. 6.0	24.2	13.3	55.0	[185]
Recent children[b]	2.0–5.0	17.2 ± 1.8	10.3 ± 1.0	60.4 ± 6.9	[189]

a) Age based on modern human standards
b) Recent children mean ± 1 s.d. (n = 20)

Dental calcification stages, age at death and MNI.

The deciduous teeth and mandible from Cueva Des-Cubierta most likely represent a single individual (MNI = 1). This assertion is based on the lack of repetition of dental elements and the compatibility of the dental wear and calcification stages of all the teeth.

Although the roots of some of the maxillary deciduous teeth are broken and the crowns of most of the mandibular deciduous teeth are missing, the preserved crowns and root formation are compatible between the maxillary and mandibular dentition where it can be directly compared. In particular, the developmental stage of the deciduous lower incisors, showing complete root formation without resorption, is compatible with that of the upper deciduous incisors. Tooth wear is either absent or slight in all the deciduous teeth. Taken together, we cannot exclude the null hypothesis that all the teeth and the mandible belong to the same individual

Based on the preserved upper and lower deciduous dentition the developmental stages are (in decreasing order): dm1 >di2 ≥ di1 > dm2. It is not possible to establish the developmental stage of either the upper or lower deciduous canine precisely, since the roots are damaged. Nevertheless, the preserved roots suggest the teeth had reached at least R¾ complete and the root formation is compatible between the upper and lower dc. While there is no M1 preserved in the Des-Cubierta child, the calcification stages of the P3 and P4 are initiated at the time of the child’s death. The relative developmental stages observed for the permanent dental elements are (in decreasing order): I1 > I2 > C > P3 > P4. The dental calcification stage of Des-Cubierta 1, falls between those in the Neandertal children from Roc de Marsal[197] and the Cova del Gegant[186], and is very similar to that of Gibraltar 2[190]. Compared with modern human standards, the dental calcification stage in Des-Cubierta 1 is compatible with an age at death of 3-5 years.

Deciduous dental morphology and metrics

The crown features in the deciduous teeth were mainly scored according to their grades of expression in adult teeth in the Arizona State University Dental Anthropology System[198] as well as those traits in adult teeth defined by Martinón-Torres et al.[19]. Dental metrics are provided in Supplementary Table 2.

Deciduous right upper first incisor (Supplementary Figure 8).

This tooth is completely preserved and shows a fully formed root. Shovelling is present on the lingual surface corresponding to ASUDAS grade 3. The shovelling can be classified as symmetric, and there is no lingual fossa associated with the shovel shape, due to the expression of a large, rounded basal eminence. The tuberculum dentale lacks a cusp with a free apex and corresponds to ASUDAS grade 1 and grade 3-4 for Martinón-

Torres et al.[19]. A moderate labial convexity is observable in occlusal view corresponding to Grade 3 (ASUDAS and Martinón-Torres et al.[19]). The single long, robust root is subtriangular, being flattened on the buccal side, with the apical foramen slightly open.

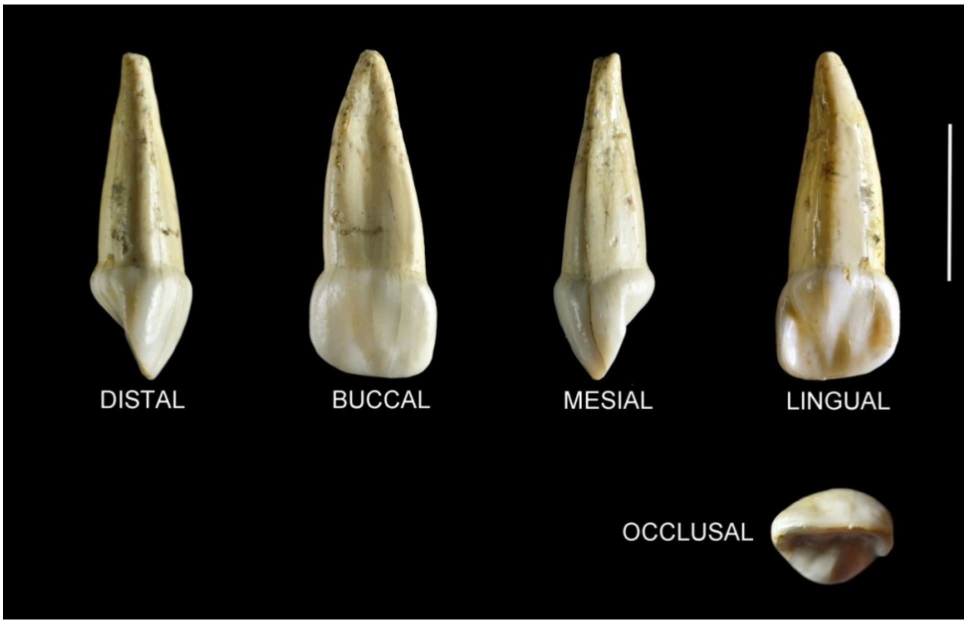

Supplementary Figure 8. Deciduous right upper first incisor (14/11/CDC/G´47/5/46). Scale bar = 1cm. Photo credit: Mario Torquemada/MAPCM.

Deciduous left upper second incisor (Supplementary Figure 9).

This tooth is complete, with only slight damage to the distal portion of the incisal surface. The tooth is shovel-shaped (Martinón-Torres et al.[19], Grade 5), and the thickened marginal ridges circumscribe a lingual fossa. On the lingual surface, a narrow crest runs superiorly from the well-developed basal eminence. There is a pronounced labial convexity of the crown (Grade 4 following ASUDAS plate for UI1 and Martinón-Torres et al.[19]). The root is long, robust and nearly rounded in cross-section.

Deciduous left upper canine (Supplementary Figure 10).

The crown of this tooth is well preserved, but the root is broken, missing approximately the apical third. In buccal and lingual views, the crown has a pentagonal shape. The mesial portion of the incisal edge is slightly shorter than the distal, but the apex is placed fairly centrally. Thickened mesial and distal marginal ridges are present on the lingual surface and canine distal accessory ridge (Grade 2 ASUDAS) is visible. The tuberculum dentale is weakly expressed, grade 0 (cingulum smooth) for Martinón-Torres et al.[19]. The labial

surface shows a moderate convexity from the base to the apex. The single root is long, robust and shows an oval cross-section.

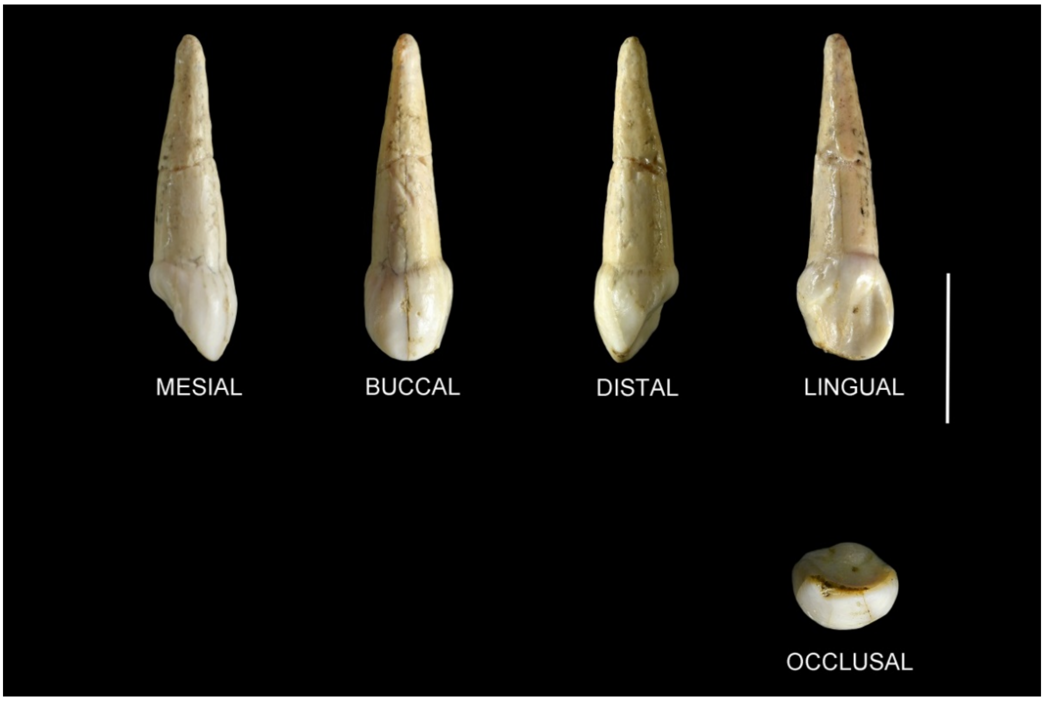

Supplementary Figure 9. Deciduous left upper second incisor (14/11/CDC/G´47/5/49). Scale bar = 1cm. Photo credit: Mario Torquemada/MAPCM.

Deciduous left upper first molar (Supplementary Figure 11).

This tooth preserves the complete and unworn crown and approximately the cervical third of the three broken roots. In occlusal view, four main cusps are present with a well-developed hypocone (Grade 5 for Martinón-Torres et al.[19]). A small metaconule (cusp 5) is also present distally (Grade 3, Martinón-Torres et al.[19]). The crown outline in occlusal view is asymmetrical, with the hypocone protruding lingually and distally. The three roots (2 buccal and 1 lingual) are unfused, showing no sign of taurodontism.

Deciduous right upper second molar (Supplementary Figure 12).

This tooth preserves a complete and unworn crown but only the very cervical portion of the root system. The roots have been broken post-mortem. Pockmarking and pitting are present on the buccal and distal surfaces of the crown and the occlusal surface also shows some alteration on the buccal and distal cusps. In occlusal view, four main cusps are present, with a well-developed hypocone (ASUDAS Grade 4 or 5). Like the dm^1, the crown outline in occlusal view is asymmetrical, with the hypocone protruding lingually

and distally. A clear crista obliqua connects the protocone and metacone. No metaconule is present, but a deep groove is present on the lingual surface of the protocone representing an expression of the Carabelli's structure.

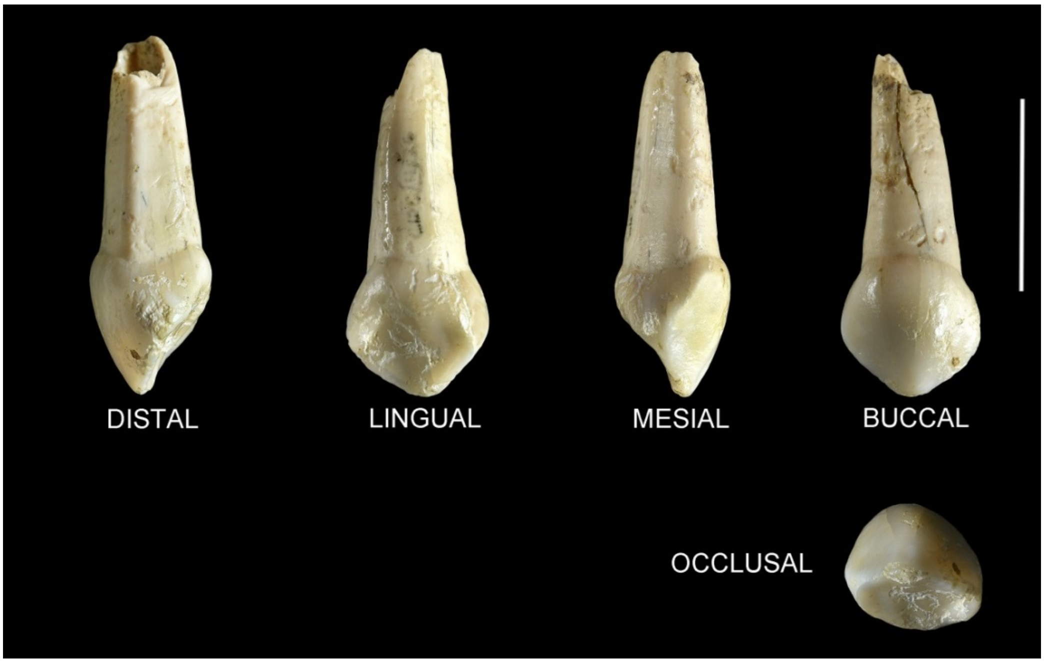

Supplementary Figure 10. Deciduous left upper canine (14/11/CDC/G´47/N5/47). Scale bar = 1cm. Photo credit: Mario Torquemada/MAPCM.

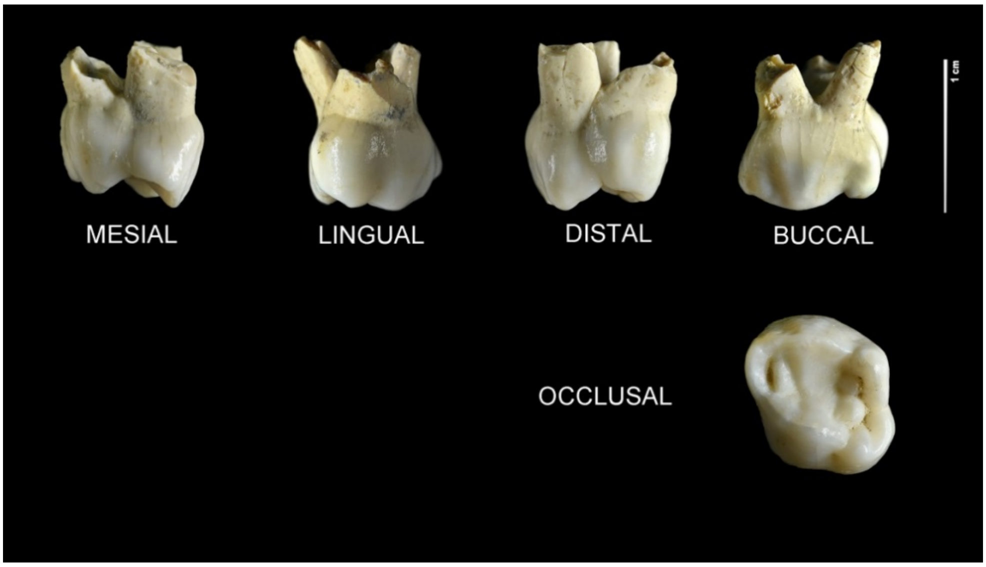

Supplementary Figure 11. Deciduous left upper first molar (14/11/CDC/H´47/N5/27). Scale bar = 1cm. Photo credit: Mario Torquemada/MAPCM.

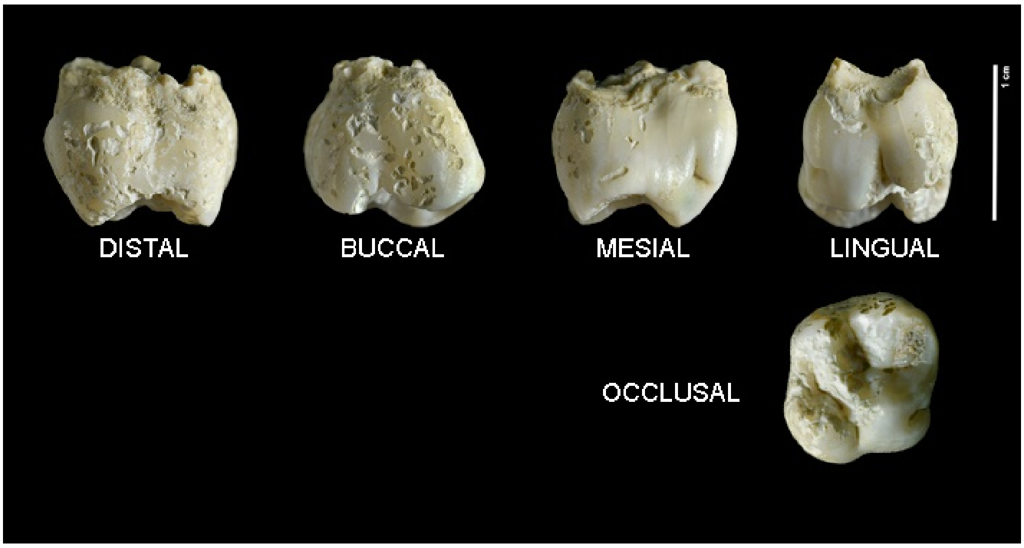

Supplementary Figure 12. Deciduous right upper second molar (14/11/CDC/G´47/N5/48). Scale bar = 1cm. Photo credit: Mario Torquemada/MAPCM.

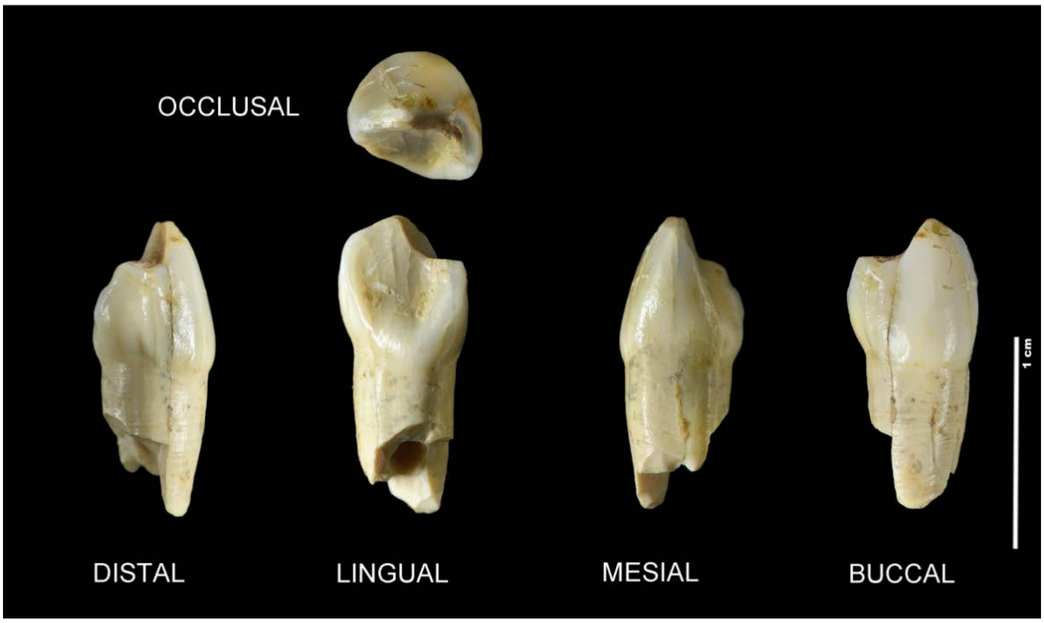

Supplementary Figure 13. Deciduous right lower canine (13/10/CDC/H'41/N100/49). Scale bar = 1cm**.** Photo credit: Mario Torquemada/MAPCM.

Deciduous right lower canine (Supplementary Figure 13).

This tooth preserves a complete crown with some damage on the distal portion of the occlusal margin. Only the apical ⅓ to ½ of the roots are present and appear to have been broken post-mortem. The mesial and distal marginal ridges are somewhat thickened, suggesting a moderate shovel-shape. The lingual basal eminence is also moderately-developed. On the lingual surface, a small localized tubercle is present at the base and is

placed slightly mesially. This could represent an expression of a canine mesial ridge (grade 2 for canine mesial ridge, Martinón-Torres et al.[19]). In lateral view, the labial surface is slightly convex from the cervix to the incisal edge. From the occlusal aspect, the labial convexity is moderate.

Supplementary Table 2. Deciduous dental dimensions (mm) in Des-Cubierta 1, Neandertals and *H. sapiens*.

	Upper di1		Upper di2		Upper dc		
Specimen/Group	MD	BL	MD	BL	MD	BL	Source
Des-Cubierta 1	7.8	6.4	6.4	5.4	7.7	6.6	Original specimen
Neandertals mean ± s.d.	7.6 ± 0.4	6.0 ± 0.3	6.1 ± 0.3	5.3 ± 0.2	7.2 ± 0.6	6.5 ± 0.6	189
Range	6.5-8.1	5.4-6.3	5.6-6.4	5.0-5.6	6.4-8.2	6.4-8.2	
n	14	14	9	9	10	10	
Fossil *H. sapiens*	7.6 ± 0.3	5.7 ± 0.6	6.7 ± 0.2	5.7 ± 0.4	7.5 ± 0.7	6.2 ± 1.1	200
Range	7.2-7.8	4.8-6.4	6.4-6.9	5.2-6.1	6.6-8.4	4.4-7.3	
n	5	5	4	4	5	5	
Recent *H. sapiens*	6.3 ± 0.6	5.0 ± 0.4	5.1 ± 0.4	4.8 ± 0.3	6.6 ± 0.5	5.9 ± 0.4	This study
Range	5.8-7.9	4.5-5.5	4.6-6.3	4.3-5.1	5.5-7.8	5.0-6.7	
n	17	17	17	17	33	33	

	Upper dm1		Upper dm2		Lower dc		
Specimen/Group	MD	BL	MD	BL	MD	BL	Source
Des-Cubierta 1	8.4	8.9	9.9	9.9	6.1	5.6	Original specimen
Neandertals	7.8 ± 0.5	9.1 ± 0.5	9.4 ± 0.6	10.2 ± 0.5	6.8 ± 0.6	6.3 ± 0.4	189
Range	7.1-8.9	8.2-9.8	8.6-10.7	9.3-11.3	5.5-8.0	5.3-7.1	
n	16	16	13	13	24	24	
Fossil *H. sapiens*	8.4 ± 0.4	8.9 ± 0.8	9.5 ± 0.5	10.4 ± 0.9	6.1 ± 0.7	5.8 ± 0.4	200
Range	7.5-8.8	7.8-10.0	8.5-10.2	9.2-11.3	5.2-7.0	4.8-6.4	
n	7	7	7	7	11	11	
Recent *H. sapiens*	7.0 ± 0.5	8.3 ± 0.7	8.9 ± 0.5	9.4 ± 0.6	5.6 ± 0.2	5.4 ± 0.3	This study
Range	6.2-8.2	7.0-10.4	8.1-9.7	7.7-10.4	5.0-5.9	4.9-5.9	
n	31	31	34	34	20	20	

In sum, several of the morphological traits in the Des-Cubierta deciduous dentition are frequently seen in Neandertals (e.g. Roc de Marsal, Chateauneuf, Pech del Azé), but are rarer in modern humans[189,199,200]. In particular, the degree of shoveling in the incisors and the shape of the crown outline in the molars are similar to the expression of these features

in Neandertal permanent teeth. While the phylogenetic polarity of individual dental traits is often difficult to establish, the combination of traits seen in the Des-Cubierta dentition is more commonly found in Neandertals[19,185,201-203].

Faunal remains

Small vertebrates

Since 2009, more than 10 tons of sediment from Levels 2, 3 and 5 of the Cueva Des-Cubierta cave have been washed and sieved. The picking out process has not yet concluded, although work with the >2 mm fraction is more advanced since such pieces can be seen with the naked eye. Thus, only qualitative data (presence/absence and relative abundance) are presented here. Levels 2, 3 and 5 are relatively rich in microvertebrates, and a large sample (more than 2000 identifiable remains) has been recovered in which at least 30 taxa have been recognised among amphibians (n = 4), reptiles (n = 4) and small mammals (n = 22) (Supplementary Table 3). Some species were recorded in all three levels, while others were found in only one. Level 2 returned the poorest sample and the smallest number of taxa identified. This is owed to its reduced extension and the cemented nature of its sediments, making the extraction of microvertebrate remains more difficult (and sometimes impossible).

From a biochronological point of view, the presence of *Microtus vaufreyi* in Level 5 (the stratigraphically lowest position) indicates this level to have been deposited during the Middle Pleistocene[170,204]. This interpretation agrees with the U-Th dates obtained for speleothems S1 and S2, located respectively below and above Level 5, which place this level within MIS6. *Microtus vaufreyi* is absent from Levels 2 and 3. The assemblages present in these last levels are very similar to one another (*Microtus arvalis*, *Microtus agrestis* and *Microtus* gr. *duodecimcostatus-lusitanicus* are the dominant species), and to those identified in other Calvero de la Higuera sites, such as in the southern sector of the Cueva del Camino cave.

In the latter site, the microvertebrate assemblages of the late MIS5 (northern and central sectors of the site) are characterized by the presence of thermophilic species, such as *Hystrix vinogradovi*, *Microtus cabrerae* and *Testudo hermanni*[33,157,158] - species absent from Levels 2 and 3 of the Cueva Des-Cubierta cave. The assemblages of these latter two levels are more similar to those found in the MIS5-4 transition of the Cueva del Camino cave (southern sector), for which the thermophilic species mentioned above are absent, and in which *Microtus arvalis*, *Microtus agrestis* and *Microtus* gr. *lusitanicus-duodecimcostatus* dominate the small-mammal assemblages[33,157]. However, the southern

sector of the Cueva del Camino cave contains remains of *Chionomys nivalis*[33,157], a species absent from Levels 2 and 3 of the Cueva Des-Cubierta cave.

Supplementary Table 3. Small vertebrates from levels 5, 3 and 2 of the Cueva Des-Cubierta cave.

Class	Order	Family	Species	Common name	Level		
					**5**	**3**	**2**
Amphibia	Anura	Alytidae	*Alytes* sp.	Midwife Toad		x	
		Bufonidae	*Bufo* gr. *spinosus-bufo*	Common Toad-Iberian Toad	x	x	x
			Epidalea calamita	Natterjack Toad	x	x	
		Ranidae	*Rana iberica*	Iberian Frog	x	x	
Reptilia	Squamata	Lacertidae	*Lacerta* s. l.	Lizard		x	
			Timon lepidus	Ocellated Lizard		x	
		Colubridae	*Coronella* cf. *austriaca*	Smooth Snake	x		
			Coronella cf. *girondica*	Southern Smooth Snake	x	x	
Mammalia	Lagomorpha	Ochotonidae	*Ochotona* cf. *pusilla*	Steppe Pika		x	
		Leporidae	*Lepus* sp.	Hare	x		
			Oryctolagus cuniculus	European Rabbit	x	x	x
	Eulipotyphla	Erinaceidae	*Erinaceus europaeus*	Western European Hedgehog	x	x	x
			Erinaceus sp.	Hedgehog	x	x	x
		Soricidae	*Sorex* sp.	Red-toothed Shrew	x	x	x
		Talpidae	*Talpa* sp.	Mole		x	
	Chiroptera	Rhinolophidae	*Rhinolophus ferrumequinum*	Greater Horseshoe Bat	x	x	
			Rhinolophus hipposideros	Lesser Horseshoe Bat	x	x	
		Vespertilionidae	*Plecotus* sp.	Long-eared Bat		x	
			Myotis myotis/blythii	Mouse-eared Bat	x	x	x
			Miniopterus schreibersii	Schreiber's Long-fingered Bat			x
	Rodentia	Gliridae	*Eliomys quercinus*	Garden Dormouse	x	x	
		Castoridae	*Castor fiber*	Eurasian Beaver	x	x	
		Cricetidae	*Arvicola sapidus*	Southern Water Vole	x	x	
			Microtus gr. *agrestis*	Field Vole	x	x	x
			Microtus arvalis	Common Vole	x	x	x
			Microtus gr. *duodecim.-lusitanicus*	Mediterranean-Lusitanian Vole	x	x	x
			Microtus vaufreyi	Vaufrey's Vole	x		
			Pliomys coronensis	Brassov Vole		x	x
			Allocricetus bursae	Extinct Hamster	x	x	
		Muridae	*Apodemus* gr. *sylvaticus-flavicollis*	Field Mouse	x	x	
				**Number of taxa:**	**22**	**26**	**11**

The microvertebrate assemblages of Levels 2 and 3 of the Cueva Des-Cubierta cave also show many similarities to those of the Navalmaíllo Rock Shelter's Level F (also deposited during the MIS5-4 transition). In Level F, the dominant species in the small-mammal assemblage are *Microtus arvalis*, *Microtus agrestis* and *Microtus* gr. *lusitanicus-duodecimcostatus*; *C. nivalis* is absent[205], as seen for Levels 2 and 3 of the Cueva Des-

Cubierta cave. However, level F includes a few remains of *Microtus cabrerae* and *Testudo hermanni*, species absent from Levels 2 and 3 of the Cueva Des-Cubierta cave, suggesting slightly warmer conditions than associated with Levels 2 and 3 of the latter cave.

The small-mammal assemblages from Levels 2 and 3 of the Cueva Des-Cubierta cave also show similarities to those from Levels 2 to 5 of the Cueva de la Buena Pinta cave, which were also deposited during the MIS4-3 transition or at the beginning of MIS3[166,167]. Once again, *Microtus arvalis*, *Microtus agrestis* and *Microtus* gr. *lusitanicus-duodecimcostatus* are dominant, but they also contain remains of *Alexandromys oeconomus*, *Lasiopodomys gregalis*, *Chionomys nivalis* or *Marmota marmota*, which are absent from the levels of the Cueva Des-Cubierta cave.

In summary, the microvertebrate assemblages of Levels 2 and 3 of the Cueva Des-Cubierta cave differ from those in the MIS5 levels of other sites of the Calvero de la Higuera Hill, in that the former contain no remains of thermophilic species. They are more similar to those recorded for the levels deposited from the MIS5-4 transition to the MIS4-3 transition, or even at the beginning of MIS3. These contain the voles *Microtus arvalis, Microtus agrestis* and *Microtus* gr. *lusitanicus-duodecimcostatus* as the dominant elements of their rodent assemblages. Therefore, it is possible to more finely tune the chronological framework provided by the dates available for the Cueva Des-Cubierta cave, which for Levels 2 and 3 are currently a maximum of *c* 130 ka (from the U/Th dating of the underlying speleothem S2) and a minimum of *c* 43 ka (the minimum age of a radiocarbon dated charcoal sample from Level 2). The age of Levels 2 and 3 should lie between the end of MIS5 (*c.* 80 ka) and the above minimum age for Level 2 (*c.* 43 ka) since: 1) they contain no elements characteristic of the Middle Pleistocene (such as *Microtus brecciensis* or *Microtus vaufreyi*) (but which are seen for in this chronology in the levels of other sites in the Calvero de la Higuera Hill), 2) nor any thermophilic elements typical of the most of MIS5, but 3) do contain remains of *Microtus arvalis*, *Microtus agrestis* and *Microtus* gr. *lusitanicus-duodecimcostatus* (the dominant elements of their small mammal assemblages). This last estimate is also consistent with the presence in these levels of the rhinoceros *Stephanorhinus hemitoechus*, the last records of which in Europe are for around 40 ka ago[206], and of *Homo neanderthalensis* in Level 2, the last records of which are also from around this date[207].

From a palaeoecological point of view, the small mammal assemblages of all three levels are dominated by voles both in terms of number of species and number of remains. This indicates the predominance of open landscapes (prairies) throughout the entire sequence. This idea is consistent with the absence of forest species, such as the red squirrel (*Sciurus vulgaris*) or the bank vole *(Clethrionomys glareolus*), which, however, are present in the late MIS5 (*c* 90 ka) sediments of the nearby Cueva del Camino cave[33,158]. The existence of some highly localized forest in a predominantly open landscape is defined by the presence of the field mouse *(Apodemus* gr. A. *sylvaticus-flavicollis*), hedgehogs *(Erinaceus europaeus* and *Erinaceus* sp.) and by the great abundance of *Bufo* gr. *Bufo spinosus* in Levels 3 and 5 (respectively 71 and 66%) compared to the much more scarce *Epidalea calamita*. In Level 3, the remains of a steppe pika (*Ochotona* cf. *pusilla*) and the greater abundance of the Iberian frog (*Rana iberica*) indicate this level was deposited during the coldest conditions of the entire sequence (and colder than at present), as seen for the central and especially the southern sectors of the Cueva del Camino cave[158]. None of the taxa present in Levels 2, 3 and 5 can be considered truly thermophilic, as would be *Pelobates cultripes*, *Malpolon monspessulanus*, *Zamenis scalaris*, *Testudo hermanni*, *Hystrix* cf. *vinogradovi* or *Microtus cabrerae* which are present in the northern and central sectors of the Cueva del Camino cave for the last part of MIS5 [MIS5c to MIS5a])[157,159].

Supplementary Table 4. Large mammals from levels 5, 3 and 2 of the Cueva Des-Cubierta cave.

Class	Order	Family	Species	Common name	Level		
					**5**	**3**	**2**
Mammalia	Primates	Hominidae	*Homo neanderthalensis*	Neandertal			x
	Carnivora	Felidae	*Felis silvestris*	Wild Cat	x	x	
			Panthera spelaea	Cave Lion	x	x	x
		Hyaenidae	*Crocuta crocuta*	Spotted Hyaena	x	x	x
		Canidae	*Cuon alpinus*	Dhole	x	x	x
		Ursidae	*Ursus* cf. *arctos*	Brown Bear	x	x	x
		Mustelidae	*Mustela* sp.	Weasel		x	
	Perissodactyla	Equidae	*Equus ferus*	Wild Horse	x	x	x
		Rhinocerotidae	*Stephanorhinus hemitoechus*	Narrow-nosed Rhinoceros		x	x
	Artiodactyla	Cervidae	*Capreolus capreolus*	European Roe-Deer	x	x	
			Cervus elaphus	Red Deer	x	x	x
		Bovidae	*Bison priscus*	Steppe Bison		x	x
			Bos primigenius	Aurochs		x	x
				**Number of taxa:**	**8**	**13**	**10**

Carnivores

The carnivore associations of Levels 2, 3 and 5 are (Supplementary Table 4):

- Level 2: *Panthera spelaea, Crocuta crocuta, Cuon alpinus.*
- Level 3: *Panthera spelaea, Crocuta crocuta, Cuon alpinus, Felis silvestris, Mustela* sp.*, Ursus* cf. *arctos.*
- Level 5: *Panthera spelaea, Crocuta crocuta,* cf. *Cuon alpinus, Felis silvestris, Ursus* cf. *arctos.*

Lions are the best represented in all three levels. The total number of lion bone remains for Levels 2 and 3 is 68. Level 3 included seven remains belonging to one adult in addition to one fragment of a tooth germ that might be part of a canine. Level 2 includes 60 remains representing at least two individuals (as shown by two right cuboid bones). Most elements from the skeleton are *manus* and *pes* (29 phalanges, 7 carpal and tarsal bones, and 6 metapodials [mostly with unfused epiphyses]) or caudal vertebrae (12). Five teeth were identified. A number of these elements belong to the same individual. It is interesting that one of the elements of the hyoidean apparatus (the basiyoideum), a very unusual find, was recovered from Level 2.

The widely used name *Panthera* (Leo) *spelaea* (Goldfuss)[208] corresponds to the species *sensu lato*[209]. Based on size, we have cautiously assigned the lion remains recovered at the Cueva Des-Cubierta cave to the Late Pleistocene subspecies *Panthera speleaea* cf. *spelaea* Goldfuss 1810. The typical features of this chrono-species (upper carnassial [P4] with a relatively narrower crown, a long metastyle and crown edge more convex buccally) were not present for analysis. P4 is the dental element of greatest diagnostic value for distinguishing *Panthera spelaea spelaea* from the Middle Pleistocene cave lion (*P. spelaea fossilis*), given the differences in the relationship between the length of the paracone and metastyle[210-212]. The lower dentition (p3, p4 and m1) also provides diagnostic information[212]. Compared to *P. spelaea spelaea, P. spelaea fossilis* has smaller incisors, less flattened but proportionally narrower canines, a narrower P2, and a somewhat narrower P4 with a shorter metastyle and smaller parastyle, and proportionally shorter main cusps on P3, p3 and p4. Unfortunately, only a P3 (12/9/CDC/5/G'46/598) and a p3 (14/11/CDC/5/F'47/5) were recovered from Level 2, but since some authors maintain that the evolution of the lineage from *P. spelaea fossilis* to *P. spelaea spelaea* involved a reduction in size[210,212], the P3 dimensions were compared with those of lions

of different chronologies. It would seem that Middle Pleistocene lions were slightly larger (as shown by the skull and postcranial bones) than Late Pleistocene specimens, although the teeth are roughly the same size[213]. Thus, slight differences in size distinguish between Middle and Late Pleistocene dentitions. However, when comparing the P3 from the Cueva Des-Cubierta cave with those recovered at other Pleistocene sites in Eurasia and Alaska, it falls at the lowest point in the size range. The scatter plot in Supplementary Figure 14 shows lion P3 dimensions for late Middle and Late Pleistocene localities. The Romain La Roche (RLR) faunal assemblage dates to either the Late Middle Pleistocene (MIS6) or Early Late Pleistocene (MIS5-4); ESR/U-Th datings suggest 150 ± 18 ka for levels VI-VII[214]. Some authors consider the RLR cave lions to be an intermediate form between *P. spelaea spelaea* and *P. spelaea fossilis* and suggest the name *Panthera (Leo) spelaea intermedia*[209]. It is clearly different from *P. spelaea fossilis* because of its smaller size. The Taubach and Ehrigsdorf (central Germany) assemblages both date to the Eemian interglacial (MIS5e) or the previous warm period (MIS7)[215]. MIS11 is assumed for the Azé 1-3 assemblage[209]. The specimens from Siberia and Alaska are all from the latest Late Pleistocene[216].

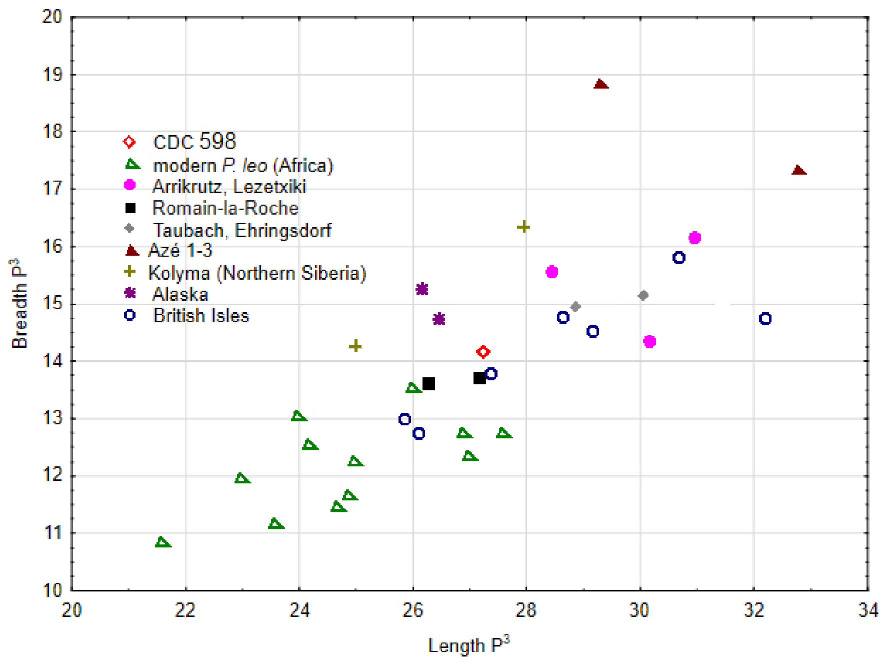

Supplementary Figure 14. Scatter plot showing the size of the *P. spelaea* P3 from the Cueva Des-Cubierta cave against that of P3 elements from other Eurasian and Alaskan populations. Data from the following: for British sites (Bleadon Cave, Kent's Hole, Sandorn Hill)[217]; for Romain-La-Roche[218]; for Azé 1-3[219]; for Kolyma (northern Siberia) and Alaska[216].

The dimensions of the P3 specimen from Level 2 are much smaller than those of the Middle Pleistocene lions from Azé, and group better with the rest of the *P. spelaea spelaea* samples as the smallest in the range (Supplementary Figure 14). These remains are thus proposed to belong to *Panthera spelaea* cf. *spelaea* Goldfuss 1810, which is in agreement with the proposed chronology for the site.

In the analyzed levels of the Cueva Des-Cubierta cave, dholes (*Cuon alpinus* Pallas 1811) are well represented, including 21 remains belonging to an adult and a juvenile (a cub with milk dentition) in Level 2, three elements in Level 3 (one being an adult mandible with its dentition), and a metapodial bone and a talus probably belonging to *C. alpinus* in Level 5. Thus, the MNI for Level 2 is at least two individuals (one adult and a cub), and for Level 3 the MNI is one adult.

Only two remains of spotted hyaenas were recovered from Levels 2 and 3 - an upper P2 and a lower carnassial, respectively. Few hyaena remains were recovered from these two levels, yet these animals are well represented in all levels at the other Pinilla del Valle/Calvero de la Higuera sites.

The wild cat (*Felis silvestris*) is also represented in Level 3 by four postcranial elements, all belonging to the same adult individual (MNI = 1). Level 2 only provided a tiny incisor of a small felid (*Felis/Lynx*).

A mandible of an adult weasel or stoat (*Mustela* sp.) was found in Level 3.

Ungulates

Among the ungulates, bovines are by far the most predominant in Level 3, and indeed throughout the stratigraphic sequence (88.18% of all identified ungulate specimens). Bovine remains include large cranial fragments (mainly the neurocranium and horn cores) and some postcranial elements. In most cases, postcranial remains are quite fragmentary and of little diagnostic value, but cranial fragments and metapodials often allow for proper identification. Both *Bison priscus* (steppe bison) (15 individuals) and *Bos primigenius* (aurochs) (five individuals) were identified, and co-occurred in Level 3. The greater number of the identified bovine elements were assigned to *Bison priscus* (15 individuals), suggesting this species to have been more common. Thus, it is reasonable to assume that most of the bovine remains that are not identifiable at the species level (eight individuals were identified only at the level of *Bos/Bison*) may well correspond to the steppe bison.

Two bovines were identified in Level 2 (39.94%), *Bison priscus* and *Bos/Bison*. Cervids are also relatively common, with at least two species represented. All cervid remains from Level 3, which include cranium and antler fragments, were identified as *Cervus elaphus* (red deer); these accounted for 3.04% of the ungulate specimens of Level 3, and 3.60% of those of Level 2. Level 3 provided six elements of the small cervid *Capreolus capreolus* (the roe deer), representing a minimum of two individuals. Rhinoceros remains, including two crania (one very well preserved) from Level 3 make up 3.66% in Level 3, and 25.83% in Level 2. Crania, teeth and postcranial remains show the morphology and size diagnostic features of *Stephanorhinus hemitoechus* (the steppe rhinoceros). Finally, two horse (*Equus ferus*) remains were found in Level 3, and one in Level 2.

The exceptionally high percentage of bovine remains is remarkable. An analysis of 86 MIS3 ungulate assemblages from Western Europe[220], showed just four of them with bovine percentages of 40-60%, with none so high as recorded for the Cueva Des-Cubierta cave. It cannot be ruled out that that this unusual composition reflects a human activity-associated bias.

This faunal complex predominantly includes species adapted to open landscapes. The steppe bison *B. priscus*, the steppe rhinoceros *S. hemitoechus*, and the horse *E. ferus* are all grazers normally associated with open grassland environments[96,221-223]. However, the presence of mixed feeders such as the red deer *C. elaphus* and the aurochs *B. primigenius* indicate some trees were present, probably patches of open forest in the surroundings of the site. The presence of the roe deer *C. capreolus* also agrees with the presence of forested areas.

Quantification of the large-mammal assemblages of Levels 3 and 2

A total of 3576 large mammal remains were identified in Level 3. Of these, 1314 were indeterminate remains, 1613 were identified anatomically and taxonomically, and 649 anatomically. Based on their size, the latter were attributed to the large, medium or small animal categories (Supplementary Table 5). The most abundantly represented taxa are bovines (*Bos/Bison*) (NISP = 1471; %NISP = 91.2%). The remaining taxa are much less abundant: *Stephanorhinus hemitoechus* (59; 3.7%), *Cervus elaphus* (49; 3.0%), *Panthera spelaea* (8; 0.5%); *Capreolus capreolus* (NISP = 6; 0.4%), *Felis silvestris* (4; 0.2%), *Cuon*

alpinus (3;0.2%), *Ursus* cf. *arctos* (3; 0.2%), *Equus ferus* (2; 0.1%), *Crocuta crocuta* (1; 0.1%), *Mustela* sp. (1; 0.1%)(Table 1).

Supplementary Table 5. Weight categories, weight ranges (kg), taxa identified, and correlation with the size classes used in studies of extant African fauna. Modified from Rodríguez-Hidalgo[103].

Size category	Weight (kg)	Taxa included	African size classes (82, 83)	Approximate weight (kg)
Large (LS)	>800<br>200-800	*Stephanorhinus hemitoechus*<br>*Bison priscus*<br>*Bos primigenius*<br>*Equus ferus*<br>*Panthera spelaea*<br>*Ursus* cf. *arctos*	Size 5<br>Size 4	900-2700<br>350-900
Medium (MS)	50-200	*Cervus elaphus*<br>*Dama dama*<br>*Crocuta crocuta*	Size 3<br>Size 2	100-350<br>20-120
Small (SS)	10-50	*Capreolus capreolus*<br>*Cuon alpinus*<br>*Felis silvestris*<br>*Mustela* sp.	Size 2<br>Size 1	20-120<br><20

Supplementary Table 6. Quantification of the abundance of large mammal species for Level 2. Number of identified specimens (NISP); relative frequency of number of identified specimens (%NISP); Minimal number of elements (MNE); relative frequency of minimal number of elements (%MNE); minimum number of individuals (NMI); relative frequency of minimum number of individuals (%NMI).

LEVEL 2	NISP	%NISP	NME	%NME	NMI	%NMI
Homo neanderthalensis	5	1.50	1	0.49	1	7.14
Felis silvestris	1	0.30	1	0.49	1	7.14
Panthera spelaea	60	18.02	51	25.00	2	14.29
Crocuta crocuta	1	0.30	1	0.49	1	7.14
Cuon alpinus	21	6.31	19	9.31	2	14.29
Carnivora indet.	13	3.90	1	0.49	1	7.14
Equus ferus	1	0.30	1	0.49	1	7.14
Stephanorhinus hemitoechus	86	25.83	56	27.45	2	14.29
Cervus elaphus	12	3.60	8	3.92	1	7.14
Bos/Bison	133	39.94	65	31.86	2	14.29
TOTAL	333	100.00	204	100.00	14	100.00
Big sized mammals	180					
Medium sized mammals	171					
Small sized mammals	12					
Indeterminable	157					

At Level 3, the skeletal representation of the bovines is characterized by the great abundance of crania (Supplementary Tables 7 and 10), with a NISP of 1294 (88% of NISP) and an NME of 28 (calculated from the tips of the horn cores), of which 15 belong to *Bison priscus*, five to *Bos primigenius* and eight to *Bos/Bison*. None of the other anatomical elements exceed an NME of 10.

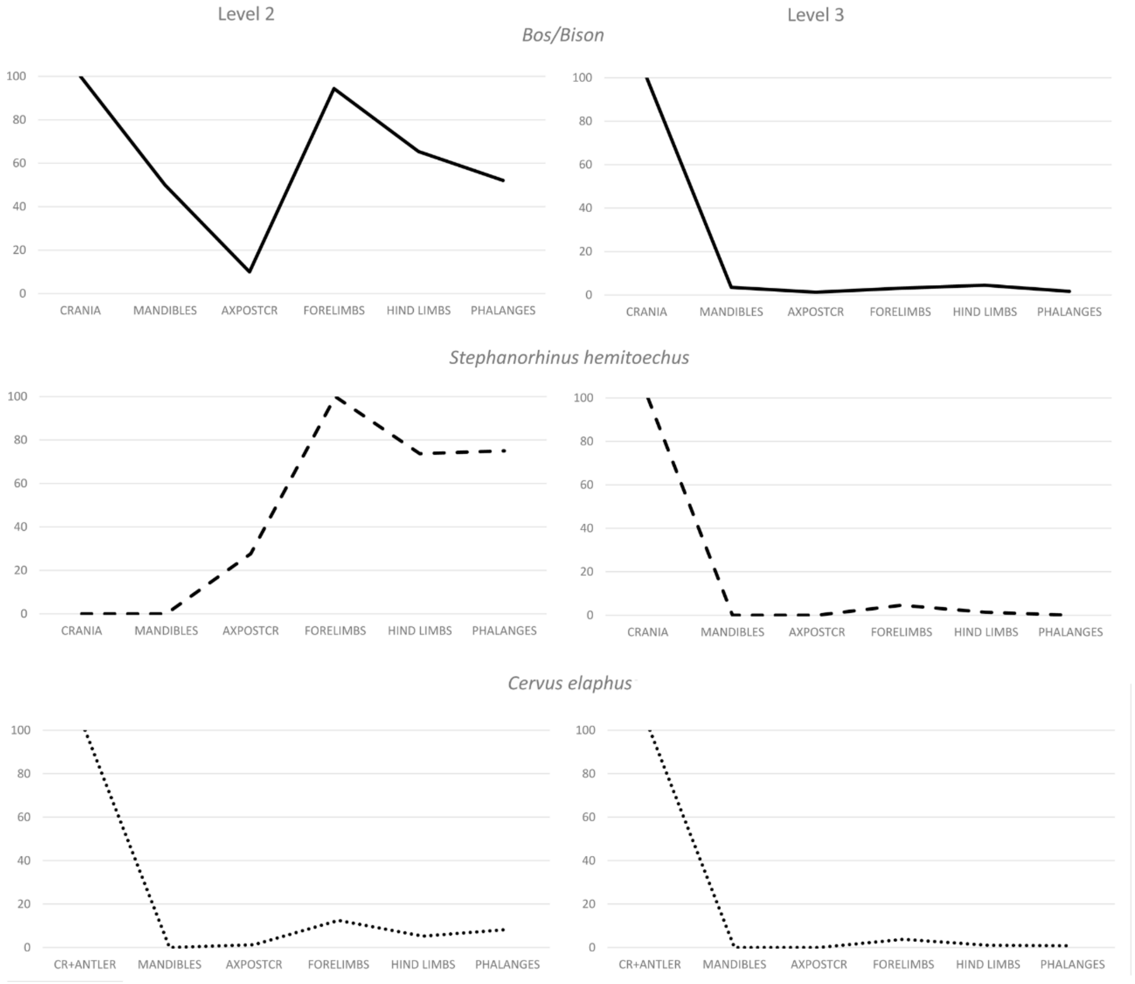

Supplementary Figure 15. Skeletal representation by anatomical group (%MAU) for *Bos/Bison*, *Stephanorhinus hemitoechus* and *Cervus elaphus* from Level 2 and Level 3. CR = cranium. For abbreviations see Supplementary Tables 7, 8 and 9.

Extended Data Fig. 6 show the skeletal representation (%MAU by anatomical group) for the main taxa (large bovines, steppe rhinoceroses and reed deer) seen in Level 3. The sharp contrast between the abundance of crania (more than 75% for all three taxa) compared to the other anatomical groups (<5%) is quite notable. The same can be seen in Supplementary Figure 15, in which the anatomical representation of these taxa in Levels 2 and 3 is compared. Postcranial remains are better represented in Level 2. When the

percentage of cranial remains is high in Level 2, it is due to the contribution of the teeth to the calculation of the abundance of this anatomical region, which, on the contrary, are very scarce in Level 3.

A total of 853 large mammal remains were identified for Level 2, 157 of which were of indeterminate identity. A total of 328 remains were identified anatomically and taxonomically, and 363 anatomically. Based on their size, the latter were attributed to the large, medium or small animal categories (Supplementary Table 5). The most abundant taxa are bovines (*Bos/Bison*) (NISP = 133; 39.9%), followed by *Stephanorhinus hemitoechus* (86; 25.8%), *Panthera spelaea* (60; 18.0%), *Cuon alpinus* (21; 6.3%), *Cervus elaphus* (12; 3.6%), *Equus ferus* (1; 0.3%), *Crocuta crocuta* (1; 0.3%) and *Felis silvestris* (1; 0.3%) (Supplementary Table 6). For bovines, an NME of 65 was determined, for which elements of the axial skeleton (cranium, hemi-mandible, teeth, vertebrae and ribs) reach an NME of 11, the forelimbs (scapula, humerus, radius, ulna, carpals and metacarpals) an NME of 17, the hind limbs (coxal, femur, tibia, tarsals) 12, and the phalanges and sesamoids, 25 (Supplementary Table 7). According to these data, and to the relative abundance (%MAU) (Supplementary Figure 15) of the anatomical elements, the representation of the anatomical elements is relatively homogeneous. A MNI of 2 was calculated from the lunate remains recovered.

Stephanorhinus hemitoechus is the second best represented taxon in terms of NISP, with the same minimum number of individuals as bovines (MNI = 2) (Supplementary Table 8). The NME is 56, for which those of the axial skeleton reach an NME of 13, the forelimbs 11, the posterior extremities 14, and the phalanges and sesamoids 18. According to these data and the %MAU of the anatomical elements, the representation of anatomical elements is again relatively homogeneous. An MNI of 2 was determined from the calcaneus elements recovered, one of them unfused and the other fused. Thus, the first belonged to a young individual and the other to an adult.

Supplementary Table 7. NISP, MNE, MAU and %MAU of the anatomical groups of the bovine remains from Levels 2 and 3. Cranium (CRN); Mandible (MN); Upper Isolated Tooth (UIT); Lower isolated tooth (LIT); Incisive tooth (IT); Atlas (ATL); Axis (AX); Cervical vertebra 3e7 (CEV 3e7); Thoracic vertebra (TV); Indeterminate vertebra (IV); Rib (RB); Sternum (ST); Lumbar vertebra (LMV); Sacrum (SA). Caudal vertebra (CAV); Scapula (SC); Humerus (HM); Radius (RD); Ulna (UL); unciform (UCF); semilunar (SEMIL); scaphoid (SCAP); Metacarpal (MC); Innominate (IM); Femur (FM); Patella (PT); Tibia (TA); Talus (AS); Calcaneus (CL); Scapho-cuboid (SCAP-CUB); Metatarsal (MT); Indeterminate metapodial (IMP); First phalange (PHF); Second phalange (PHS); Third phalange (PHT).

Bos/Bison									
		**Level 2**				**Level 3**			
**Anat Groups**	**Element**	**NISP**	**NME**	**MAU**	**%MAU**	**NISP**	**NME**	**MAU**	**%MAU**
AxCr	CRN	49	1	1	100	1294	28	28	100
AxMn	MN	3	1	0.5	50	7	2	1	3.57
AxPost	UIT	3	1	0.10	10	2	2	0.36	1.29
	LIT	3	1			4	3		
	IT	7	1			9	6		
	ATL	0	0			8	6		
	AX	1	1			6	3		
	CEV	3	2			7	3		
	RB	3	1			1	1		
	LMV	1	1			7	4		
	CAV	1	1			1	1		
FORELIMBS	SC	2	1	0.94	94.44	13	2	0.875	3.13
	HM	5	2			13	4		
	RD	1	1			4	2		
	UL	1	1			1	1		
	MAG-TRAP	2	2			1	1		
	UNF	2	2			1	1		
	PYR	1	1			1	1		
	SEMIL	4	4			4	2		
	MC	4	3			5	2		
HIND LIMBS	FM	1	2	0.65	65.38	3	2	1.26	4.51
	PT	1	1			1	1		
	LTM	2	2			10	3		
	AS	3	3			3	3		
	CL	2	1			4	4		
	SCAP-CUB	1	1			2	2		
	IMP	1	1			1	1		
	MT	1	1			11	6		
PH+SE	PHF	5	5	0.52	52.08	2	2	0.46	1.64
	PGS	5	5			5	5		
	PHT	3	3			4	4		
	SE	12	12			0	0		
	Total	133	65			1471	108		

Supplementary Table 8. NISP, MNE, MAU and %MAU of the anatomical groups of rhinoceros remains from Levels 2 and 3. Cranium (CRN); Mandible (MN); Upper Isolated Tooth (UIT); Lower isolated tooth (LIT); Incisive tooth (IT); Atlas (ATL); Axis (AX); Cervical vertebra 3e7 (CEV 3e7); Thoracic vertebra (TV); Indeterminate vertebra (IV); Rib (RB); Sternum (ST); Lumbar vertebra (LMV); Sacrum (SA). Caudal vertebra (CAV); Scapula (SC); Humerus (HM); Radius (RD); Ulna (UL); unciform (UCF); semilunar (SEMIL); scaphoid (SCAP); Metacarpal (MC); Innominate (IM); Femur (FM); Patella (PT); Tibia (TA); Talus (AS); Calcaneus (CL); Scapho-cuboid (SCAP-CUB); Metatarsal (MT); Indeterminate metapodial (IMP); First phalange (PHF); Second phalange (PHS); Third phalange (PHT).

Stephanorhinus hemitoechus									
		**Level 2**				**Level 3**			
**Anat groups**	**Element**	**NISP**	**NME**	**MAU**	**%MAU**	**NISP**	**NME**	**MAU**	**%MAU**
AxCr	CRN	0	0	0	0	52	2	2.00	100
AxMn	MN	0	0	0	0	0	0	0.00	0
Axpostcr	UIT	6	1	0.14	27.66	2	2	0.03	1.60
	LIT	7	1			1	1		
	ATL	0	0			0	0		
	AX	0	0			0	0		
	VCE	0	0			0	0		
	RB	2	2			0	0		
	LMV	0	0			0	0		
	CAV	9	9			0	0		
FORELIMBS	SC	0	0	0.5	100	0	0	0.09	4.55
	HM	4	1			1	1		
	RD	2	1			0	0		
	UL	2	2			0	0		
	MAGN	1	1			0	0		
	UCF	2	2			0	0		
	PYR	1	1			0	0		
	SEMIL	0	0			0	0		
	II MC	1	1			1	1		
	III MC	1	1			0	0		
	IV MC	1	1			0	0		
HIND LIMBS	IM	1	1	0.37	73.68	2	1	0.03	1.32
	FM	1	1			0	0		
	PT	0	0			0	0		
	TA	0	0			0	0		
	AS	2	2			0	0		
	CL	3	2			0	0		
	SCAF	0	0			0	0		
	CUB	1	1			0	0		
	CUN 2	1	1			0	0		
	CUN 3	1	1			0	0		
	IMP	5	1			0	0		
	II MT	3	1			0	0		
	III MT	2	1			0	0		
	IV MT	3	2			0	0		
PH+SE	PHF MC/MT II	3	2	0.38	75.00	0	0	0	0
	PHF MC/MT III	7	5			0	0		
	PHF MC/MT IV	2	1			0	0		
	PHS MC/MT II	2	2			0	0		

	PHS MC/MT III	3	2			0	0		
	PHS MC/MT IV	3	2			0	0		
	PHT MC/MT II	1	1			0	0		
	PHT MC/MT III	1	1			0	0		
	PHT MC/MT IV	1	1			0	0		
	SE	1	1			0	0		
	Total	86	56			59	8		

Supplementary Table 9. NISP, MNE, MAU and %MAU of the anatomical groups of red deer remains from Levels 2 and 3. Cranium (CRN); Mandible (HMN); Upper Isolated Tooth (UIT); Lower isolated tooth (LIT); Incisive tooth (IT); Atlas (ATL); Axis (AX); Cervical vertebra 3e7 (CEV 3e7); Thoracic vertebra (TV); Indeterminate vertebra (IV); Rib (RB); Sternum (ST); Lumbar vertebra (LMV); Sacrum (SA). Caudal vertebra (CAV); Scapula (SC); Humerus (HM); Radius (RD); Ulna (UL); unciform (UCF); semilunar (SEMIL); scaphoid (SCAP); Metacarpal (MC); Innominate (IM); Femur (FM); Patella (PT); Tibia (TA); Talus (AS); Calcaneus (CL); Scapho-cuboid (SCAP-CUB); Metatarsal (MT); Indeterminate metapodial (IMP); First phalange (PHF); Second phalange (PHS); Third phalange (PHT).

Cervus elaphus									
		**Level 2**				**Level 3**			
**Anat groups**	**Element**	**NISP**	**NME**	**MAU**	**%MAU**	**NISP**	**NME**	**MAU**	**%MAU**
AxCr+Ant	CRN	5	1	1	100	44	5	5	100
AxMn	MN	0	0	0	0	0	0	0	0
AxPost	ATL	0	0	0.0125	1.25	0	0	0	0
	AX	0	0			0	0		
	CEV	0	0			0	0		
	TV	0	0			0	0		
	IV	0	0			0	0		
	RB	0	0			0	0		
	ST	0	0			0	0		
	LMV	0	0			0	0		
	SA	1	1			0	0		
FORELIMBS	SC	1	1	0.13	12.5	2	2	0.19	3.75
	HM	1	1			1	1		
	RD	0	0			0	0		
	UL	0	0			0	0		
	UCF	0	0			0	0		
	SEMIL	0	0			0	0		
	SCAP	0	0			0	0		
	MC	0	0			0	0		
HIND LIMBS	IM	0	0	0.05	5.26	0	0	0.05	1.05
	FM	0	0			1	1		
	PT	0	0			0	0		
	TA	1	1			0	0		
	AS	0	0			0	0		
	CL	0	0			0	0		
	SCAP-CUB	0	0			0	0		
	MT	0	0			0	0		
	IMP	0	0			0	0		
PH+SE	PHF	1	1	0.08	8.33	0	0	0.04	0.83
	PGS	0	0			0	0		
	PHT	0	0			1	1		
	SE	2	2			0	0		
Total		12	8			49	10		

Supplementary Table 10. List of crania recovered from Level 3 of the Cueva Des-Cubierta cave, their taxonomic identification, and the initials given to their main fragments (MAPCM = Museo Arqueológico y Paleontológico de la Comunidad de Madrid).

Number	Taxonomic identification	Accession codes for the main fragments (MAPCM).
1	*Bos primigenius*	10/19/CDC/H'41/1/32, 11/13/CDC/H'41/1/25
2	*Bison priscus*	10/19/CDC/I'41/1/29, 10/19/CDC/I'40/1/21/24
3	*Bos/Bison*	10/19/CDC/I'43/1/14
4	*Bos/Bison*	10/19/CDC/H'43/1/20
5	*Bos/Bison*	10/19/CDC/I’40/1/27, 90/23/CDC/H'40/1/2
6	*Bison priscus*	11/13/CDC/G'42/1/14
7	*Bison priscus*	11/13/CDC/H'42/100/18/24, 12/9/CDC/G'42/100/21
8	*Bison priscus*	11/13/CDC/I'41/1/19
9	*Bos primigenius*	11/13/CDC/I'40/100/9
10	*Bison priscus*	11/13/CDC/I'40/1/60, 9/23/CDC/H’40/1/1
11	*Bos/Bison*	12/9/CDC/I'38/100/12, 12/9/CDC/I'39/1/19
12	*Bos/Bison*	12/9/CDC/I'38/1/22a, 12/9/CDC/I'39/1/23
13	*Bos/Bison*	10/19/CDC/G'41/1/16e
14	*Bison priscus*	12/9/CDC/I'39/1/22b, 14/11/CDC/J'38/101/175, 14/11/CDC/J'38/101/217/233
15	*Bos primigenius*	12/9/CDC/G'45/1/82
16	*Bos primigenius*	13/10/CDC/J'40/101/74
17	*Bison priscus*	13/10/CDC/J'39/101/91/95
18	*Bos/Bison*	13/10/CDC/J'37/101/23,15/13/CDC/J'36/101/97/98/99
19	*Bison priscus*	14/11/CDC/J'36/251/26
20	*Bison priscus*	14/11/CDC/G’45/101/4, 15/13/CDC/G'45/101/91
21	*Cervus elaphus*	14/11/CDC/I'41/101/47, 14/11/CDC/I'40/101/53
22	*Stephanorhinus hemitoechus*	15/13/CDC/H’42/101/50, 15/13/CDC/I’42/101/179
23	*Cervus elaphus*	15/13/CDC/J’40/101/4/15
24	*Bison priscus*	15/13/CDC/J'39/101/3, 12/9/CDC/I'39/101/25
25	*Bison priscus*	15/13/CDC/J'39/101/163/185,15/13/CDC/K'40/101/1
26	*Bison priscus*	15/13/CDC/K'39/101/110/169, 16/12/CDC/K’39/101/169
27	*Stephanorhinus hemitoechus*	16/12/CDC/J’40/101.2/681
28	*Bos/Bison*	16/12/CDC/J'40/101.2/605, 16/12/CDC/I'40/101.2/58
29	*Bison priscus*	17/26/CDC/J'37/101/54/120
30	*Bison priscus*	17/26/CDC/K'38/101/235-240
31	*Bison priscus*	17/26/CDC/I'43/101.C/96/97
32	*Cervus elaphus*	17/26/CDC/K'38/101/182
33	*Bos primigenius*	18/29/CDC/H'44/101/272
34	*Cervus elaphus*	20/14/CDC/K'39/101.2/33
35	*Cervus elaphus*	20/14/CDC/G'44/101/13

Palynology

Only Levels 5, 3 and 2 of the Cueva Des-Cubierta cave have been examined palynologically, a consequence of their accessibility (owed to their outcropping conditions), the stage of excavation reached, and the degree of cementation affecting the different cave sediments. The following results have so far been obtained.

- Level 5. The 11 samples available for this level reveal a low taxonomic diversity, with just 18 palynomorphs, an aquatic taxon, monolete and trilete spores, and 11 types of non-pollen microfossil (NPM) (Supplementary Figure 16). The scant representation of *Pinus*, and of mesophilic woodland and riparian species, indicates an open landscape, which in turn explains the presence of NPM type 207 (*Glomus fasciculatum*). Herbaceous species dominate (Supplementary Figure 17), especially steppe species, accompanied by nitrophilic taxa. Tree taxa, along with mesophilic and thermophilic taxa, *Pinus* and *Juniperus* are represented (if poorly) largely in the bottom half of Level 5, with taxa classified as "other plants" dominating the upper half. Such a composition and behaviour (Supplementary Figure 17) suggest a progressive drying, backed up by the appearance of more dryland NPMs and the installation of Mediterranean woodland (Supplementary Figure 16). At the very top of the Level, however, the latter woodland type substitutes mesophilic woodland in some samples, and there is a notable reappearance of *Pinus*. The dispersion and diversity of the data confirm the above information, and reveal Level 5 to be the best represented in terms of mesophilic and nitrophilic species (Supplementary Figure 17). In addition, two compositional phases are quite clear: L-5Lower, characterised by the greater presence of mesophilic woodland and nitrophilic plants, plus some *Juniperus*; and L-5Upper, characterised by the scant representation of trees (with *Juniperus* as the major element despite its very small presence), and the great expansion of cosmopolitan plants (suggesting conditions to have been colder and drier). Since Level 5 lies between the speleothems S1 and S2, dated to 135 ka and 185 ka respectively, it must have formed during MIS6. This would explain the low taxonomic diversity and the dominance of open landscapes. The change in plant cover between L-5Lower and L-5Upper also indicates that conditions became progressively colder.

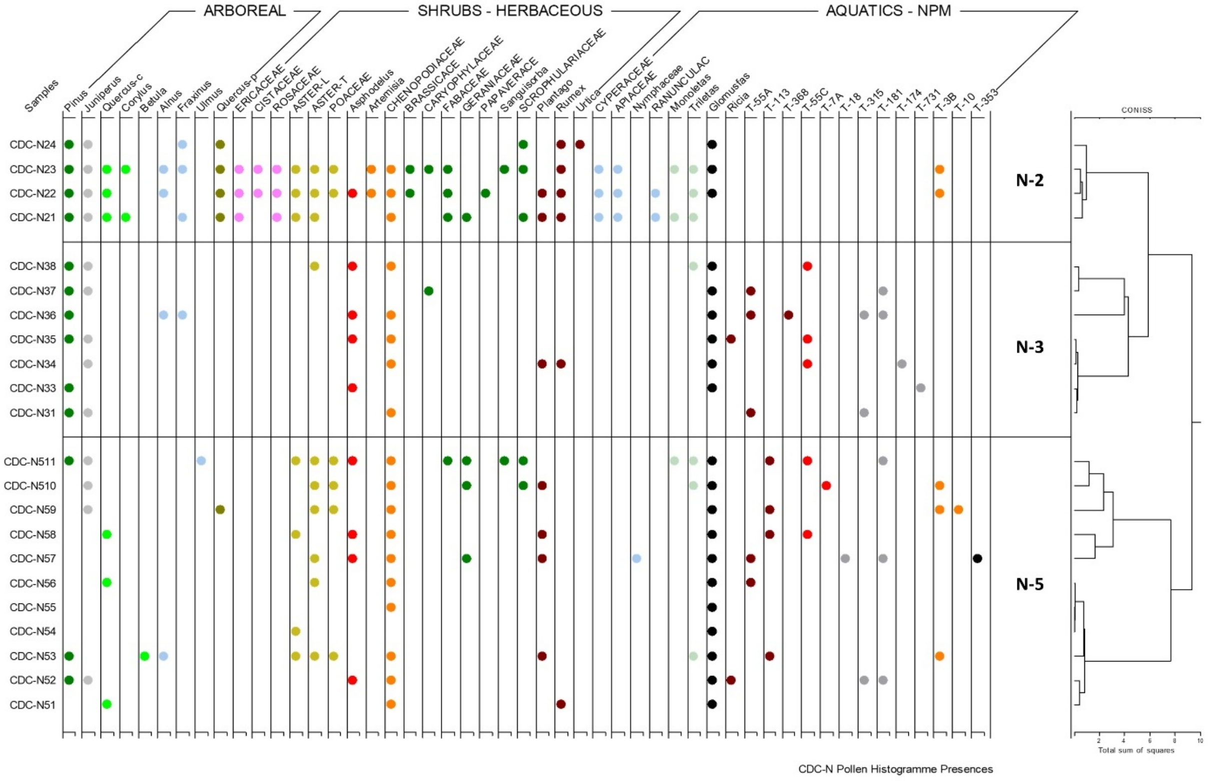

Supplementary Figure 16. Presence of pollen types in Levels 5, 3 and 2 in the central sector of the Cueva Des-Cubierta cave.

- Level 3. The eight samples available for this Level show the smallest taxonomic diversity for the entire sequence (Supplementary Figure 16), with just 10 palynomorphs, 8 NPM, and trilete spores. However, it is in these samples that trees are best represented (Supplementary Figure 17), a consequence of the expansion of *Pinus* and *Juniperus*. Mesophilic taxa were represented in sample CDC-L3-6, after which point in time *Pinus* and *Juniperus* became significantly less abundant in favour of herbaceous plants. Steppe taxa fluctuate in abundance across Level 3, but are always less abundant than in Level 5 (in sample CDC-L3-7 they were not represented at all). The expansion of *Juniperus* and the appearance of steppe taxa (Supplementary Figure 17) indicate a time of colder, drier conditions. This would explain the reduction seen in herbaceous and nitrophilic taxa, and the increase in the presence of carbonicolous NPMs (types T-55 C and 7 A) and *Asphodelus*, both associated with the presence of fire (Supplementary Figure 16). Level 3 represents the culmination of dry, cold conditions, which, bearing in mind the available dating information, situate its formation during MIS4 or at the beginning of MIS3.

- Level 2. Compared to Levels 5 and 3, the four samples available for Level 2 bear witness to a great increase in diversity for all groups of vegetation, except for the NPMs (24 palynomorphs, 2 aquatic taxa, monolete spores, and 8 NPMs). *Pinus* dominates, but mesophilic and Mediterranean woodland are still represented, as are (to a lesser extent) riparian and aquatic taxa (Supplementary Figure 16). This, plus the decline in *Juniperus* and steppe taxa (only represented in sample L-2-2) (Supplementary Figure 17), show conditions became warmer. In agreement with the dating information available, this suggests the formation of Level 2 may have occurred around the beginning of MIS 3.

Discussion

The above results show how the ecosystem responded to changes in the climate; the regional vegetation changed with alterations in temperature and the amount of available water. From a compositional point of view, the vegetation was rather homogeneous, with fluctuations in taxonomic diversity responding to improvements (L5Lower and L2) and worsenings (L5Upper) in the climate, or indeed the installation of rather more adverse conditions (Level 3). The continued presence of NPM type 207 (*G. fasciculatum*) reveals the dominance of open landscapes at the local level. However, factors other than changes in the climate could have affected the plant cover. High densities of wild ungulates could have interfered with the regeneration of woody vegetation[224].

Also noteworthy is the progressive reduction in mesophilic woodland taxa over Levels 5 and 2, and their almost complete absence from Level 3. Mediterranean woodland developed over Level 2, and an expansion of xeric and steppe vegetation occurred during the time of Level 3. Thus, the landscape was quite open in dry periods (cold or otherwise), in agreement with conclusions reached in studies of the herpetofauna of the Jarama and Manzanares valleys[225]. The detection of riparian woodland - although with fluctuations in its abundance - clearly indicates that environments suitable for it were present, their size dependent on the amount of liquid water available.

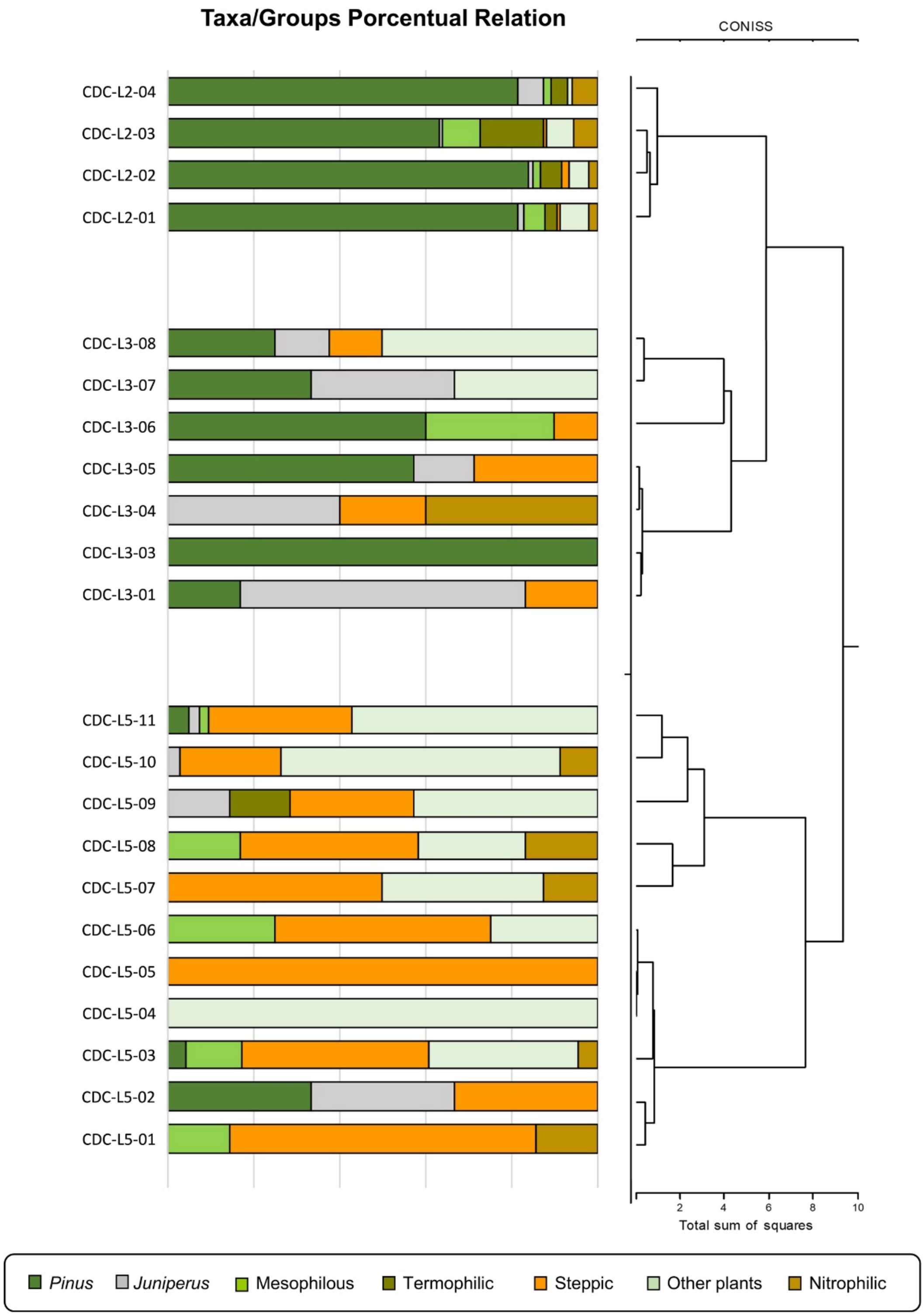

Supplementary Figure 17. Histogram showing the taxa identified and the most significant groupings of sequence CDC-19 (L5 = Level 5; L3 = Level 3; L2 = Level 2). Arboreal mesophilic taxa include *Betula*, *Corylus*, *Juglans*, *Tilia*, and deciduous *Quercus*, and riparian species such as *Alnus*, *Fraxinus* and *Ulmus*; thermophilic (Mediterranean) taxa include evergreen *Quercus* and Ericaceae, Cistaceae and Rosaceae: steppic herbaceous plants include *Artemisia* and Chenopodiaceae; nitrophilic taxa include *Plantago*, Polygonaceae, Rubiaceae, *Rumex* and *Urtica*; 'other plants' = all other herbaceous plants.

The representation of nitrophilic plants (which were at their least abundant over Level 3) can be understood in terms of the number of grazing animals present (which leave behind their manure).

Given the dating information available, Level 5 reflects fluctuations that occurred during the overall cold period of MIS6. Level 3, however, represents the most extreme climate, perhaps corresponding to MIS4. The slight improvement seen in Level 2 points to the start of MIS3.

Conclusions

Levels 5-2 of the Cueva Des-Cubierta cave reflect the response of the regional plant cover to changes in temperature and available water. From a compositional point of view, the vegetation was rather homogeneous, with fluctuations in taxonomic diversity responding to improving or worsening climatic conditions (Level 5 and Level 2, respectively), or indeed the installation of rather more adverse conditions (Level 3). The continued presence of NPM type 207 (*G. fasciculatum*) reveals the dominance of open landscapes at the local level. Over Level 5, mesophilic and Mediterranean woodland gradually declined, becoming absent in Level 3, but then reappeared in Level 2. Level 3 reveals the appearance of pine forests, the expansion of *Juniperus*, and the appearance of steppe vegetation, while over the time of Level 2 these latter two types of vegetation suffered a marked reduction.

1- The detection of riparian woodland (albeit fluctuating in terms of its abundance), bears clear witness to the permanence of such places, their size dependent on the availability of liquid water. This suggests large water courses flowed through gallery forest. The identification of aquatic plants in (almost exclusively) Level 2 suggests that times existed when it was wetter or water was more consistently available.

2- The nitrophilic species throughout the sequence - continuous in Level 2, less common in Level 5, and sporadic in Level 3 - might be understood in terms of the abundance of grazing animals.

3- Level 3 appears to represent a dry period, while Level 5 shows changes in the available water, and Level 2 appears as quite wet. Level 3 appears to have been a time of more extreme cold and dryness than Levels 5 and 2.

4- Within this climatic framework, the presence of *Asphodelus* (a pyrophyte) and carbonicolous NPMs (types 55 C and 7 A), which are found at the top of Level 5 and throughout Level 3, but are only eventual in Level 2, might indicate a relationship between cold and fire.

5- The behaviour of the vegetation and the available dating information place Level 5 in the isotopic stage of MIS6, Level 3 within MIS4 or a cold period at the beginning of MIS3, and Level 2 within a more temperate period at the beginning of MIS3.

Lithic Industry

As seen for the other Calvero de la Higuera sites, quartz was the main knapped material associated with the Cueva Des-Cubierta cave (76.6% in Level 2, 72.8% in Level 3), followed by chert, porphyry, rock crystal, and other materials.

Quartz is the most abundant knappable resource in the Guadarrama Mountain range. It is available in primary position in the form of dykes and outcrops, in secondary position as cobbles and pebbles transported by the many secondary water courses (tributaries and temporary streams) in the Lozoya River Valley[226], and on slopes where it is has become available through natural erosion. Its use at the Cueva Des-Cubierta cave highlights the expedient exploitation of local resources.

Between 2009 and 2019, the lithic assemblages recovered from the main occupations of the cave (n = 734 from Level 2, and n = 1421 from Level 3) included a set of anvils, hammerstones, cores, flakes and Mousterian shaped tools (Supplementary Tables 11 and 12). Quartz tends to break and shatter irregularly, and conchoidal fractures that allow the shape of final product to be predicted, do not always occur. The Neanderthal toolmakers that used this cave therefore required a good knowledge of the surrounding landscape; they needed to know where the best quality nodules that would allow the production of larger, better flakes, could be acquired. Collecting the raw material used for the large anvils would have demanded more planning and a greater expenditure of energy since it all had to be transported uphill to the cave (Supplementary Figure 18). Indeed, the economy of raw material use at the site implies strategic decisions had to be taken regarding the acquisition of different materials for use in specific contexts and activities. Certainly, at the other Calvero de la Higuera hill sites (such as the Navalmaíllo Rock Shelter where different subsistence activities took place)[162,163], the lithic tools that have been found are smaller, suggesting a different resource collection strategy was followed.

Quartz cores were the knapped materials most commonly recovered, and usually reflected a mid-to-late state of exploitation (determined taking into account the amount of remaining cortex) (Extended Data Fig. 1 and Supplementary Figure 19 and 20.1-4). The mean dimensions of Level 2 cores were 55 x 44 x 32 mm, while for those from Level 3 they were 59 x 46 x 31 mm.

Supplementary Table 11. Tool types (number and percentage) by raw material. Level 2.

Level 2	Quartz	Chert	Quartzite	Porphyry	Rock crystal	Sandstone	Limestone	Other rocks	Total
Pebbles	4 (0.7%)	0	1 (4%)	2 (10%)	0	0	0	5 (6%)	12 (1.6%)
Hammerstones	8 (1.4%)	0	3 (12%)	6 (30%)	0	23(42.9%)	1 (100%)	22 (26.5%)	43 (5.9%)
Anvils	1 (0.2)	0	0	0	0	0	0	3 (3.6%)	4 (0.5%)
Fractured pebbles	3 (0.5%)	0	5 (20%)	0	0	1 (14.3%)	0	7 (8.4%)	16 (2.2%)
**Total pebbles**	**16 (2.8%)**	**0**	**9 (36%)**	**8 (40%)**	**0**	**4 (57.1%)**	**1 (100%)**	**37 (44.6%)**	**75 (10.2%)**
Cores on pebble	45 (8%)	1 (5.3%)	3 (12%)	3 (15%)	0	0	0	1 (1.2%)	53 (7.2%)
Cores on flake	9 (1.6)	1 (5.3%)	2 (8%)	0	1 (5.9%)	0	0	0	13 (1.8%)
**Total cores**	**54 (9.6%)**	**2 (10.5)**	**5 (20%)**	**3 (15%)**	**1 (5.9%)**	**0**	**0**	**1 (1.2%)**	**66 (9%)**
Tools on pebble	0	0	0	0	0	0	0	0	0
Tools on flake	58 (10.3%)	2 (10.5%)	1 (4%)	3 (15%)	4 (23.5%)	0	0	1 (1.2%)	69 (9.4%)
**Total shaped tools**	**58 (10.3%)**	**2 (10.5%)**	**1 (4%)**	**3 (15%)**	**4 (23.5%)**	**0**	**0**	**1 (1.2%)**	**69 (9.4%)**
Flakes	338 (60.1%)	9 (47.4%)	6 (24%)	4 (20%)	7 (41.2%)	0	0	1 (1.2%)	365 (49.7%)
**Total simple products**	**338 (60.1%)**	**9 (47.4%)**	**6 (24%)**	**4 (20%)**	**7 (41.2%)**	**0**	**0**	**1 (1.2%)**	**365 (49.7%)**
Fragments	84 (14.9%)	1 (5.3%)	3 (12%)	0	5 (29.4%)	0	0	10 (12%)	103 (14%)
Undetermined	12 (2.1%)	5 (26.3%)	1 (4%)	2 (4%)	0	3 (42.9%)	0	33 (39.8%)	56 (7.6%)
**Total**	**562 (76.6%)**	**19 (2.6%)**	**25 (3.4%)**	**20 (2.7%)**	**17 (2.3%)**	**7 (0.9%)**	**1 (0.1%)**	**83 (11.3%)**	**734**

Supplementary Table 12. Tool types (number and percentage) by raw material. Level 3.

Level 3	Quartz	Chert	Quartzite	Porphyry	Rock crystal	Sandstone	Limestone	Other rocks	Total
Pebbles	6 (0.6%)	0	2 (4.9%)	8 (14.5%)	0	6 (20%)	6 (75%)	29 (15.4%)	57 (4%)
Hammerstones	30 (2.9%)	0	5 (12.2%)	16 (29.1%)	0	6 (20%)	2 (25%)	39 (20.7%)	98 (6.9%)
Anvils	4 (0.4)	0	0	3 (5.5%)	0	0	0	8 (4.3%)	15 (1%)
Fractured pebbles	9 (0.9)	0	5 (12.2%)	3 (5.5%)	0	1 (3.3%)	0	10 (5.3%)	28 (2%)
**Total pebbles**	**49 (4.7%)**	**0**	**12 (29.3%)**	**30 (54.5%)**	**0**	**13 (43.3%)**	**8 (100%)**	**86 (45.7%)**	**198 (13.9%)**
Cores on pebble	94 (9.1%)	2 (7.4%)	6 (14.6%)	5 (9.1%)	2 (5.3%)	2 (6.7%)	0	4 (2.1 %)	115 (8.1%)
Cores on flake	43 (4.2%)	3 (11.1%)	1 (2.4%)	0	2 (5.3%)	0	0	2 (1.1 %)	51 (3.6%)
**Total cores**	**137 (13.2%)**	**5 (18.5%)**	**7 (17.1%)**	**5 (9.1%)**	**4 (10.5%)**	**2 (6.7%)**	**0**	**6 (3.2%)**	**166 (11.7%)**
Tools on pebble	1 (0.1%)	0	0	0	0	0	0	0	1 (0.1%)
Tools on flake	91 (8.8%)	4 (14.8%)	1 (2.4%)	1 (1.8%)	7 (18.4%)	0	0	2 (1.1 %)	106 (7.5%)
**Total shaped tools**	**92 (8.9%)**	**4 (14.8%)**	**1 (2.4%)**	**1 (1.8%)**	**7 (18.4%)**	**0**	**0**	**2 (1.1%)**	**107 (7.5%)**
Flakes	626 (60.5%)	17 (63%)	15 (36.6%)	16 (29.1%)	23 (60.5%)	2 (6.7%)	0	17 (9%)	716 (50.4%)
**Total simple products**	**626 (60.5%)**	**17 (63%)**	**15 (36.6%)**	**16 (29.1 %)**	**23 (60.5%)**	**2 (6.7%)**	**0**	**17 (9%)**	**716 (50.4%)**
Fragments	124 (12%)	1 (3.7%)	4 (9.7%)	3 (5.5%)	4 (10.5%)	1 (3.3%)	0	14 (7.4%)	151 (10.6%)
Undetermined	6 (0.6%)	0	2 (4.9%)	0	0	12 (40%)	0	63 (33.5%)	83 (5.8%)
**Total**	**1034 (72.8%)**	**27 (1.9%)**	**41 (2.9%)**	**55 (3.9%)**	**38 (2.7%)**	**30 (2.1%)**	**8 (0.6%)**	**188 (13.2%)**	**1421**

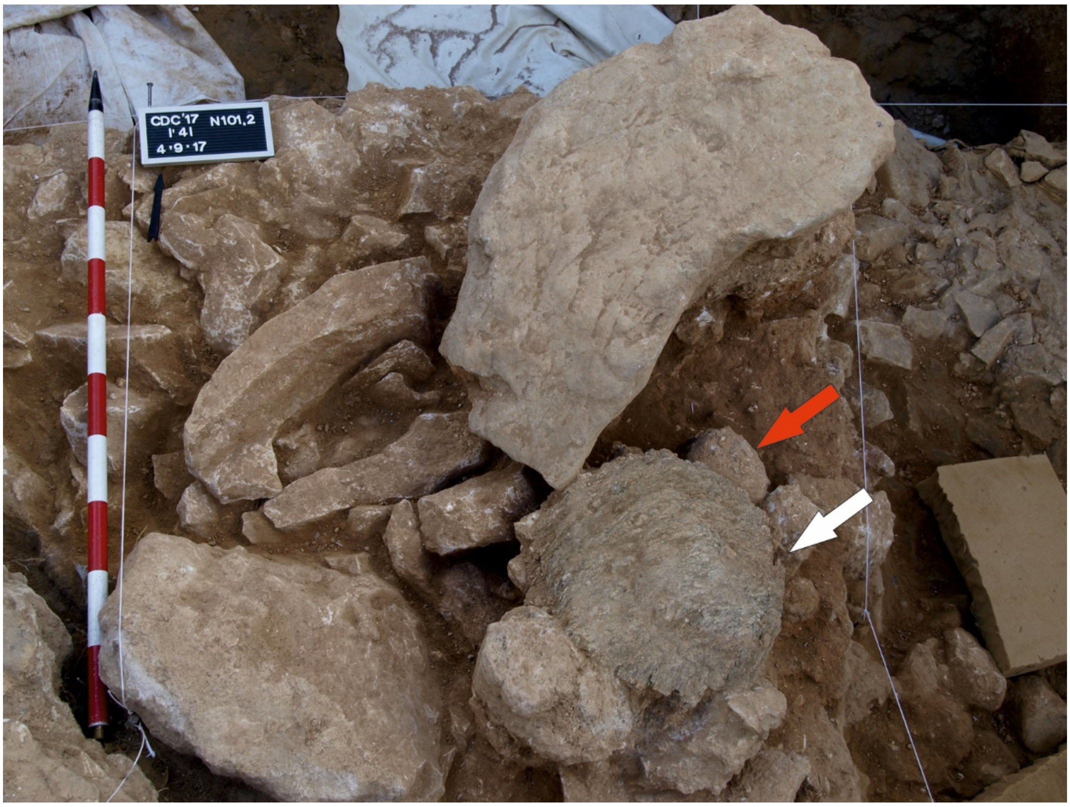

Supplementary Figure 18. Gneiss anvil in a poor state of preservation (white arrow) (17/26/CDC/I'41/101.2/21) associated with a limestone hammerstone (red arrow) (17/26/CDC/I'41/101.2/11) (Level 3). Photo credit: Javier Trueba/MSF.

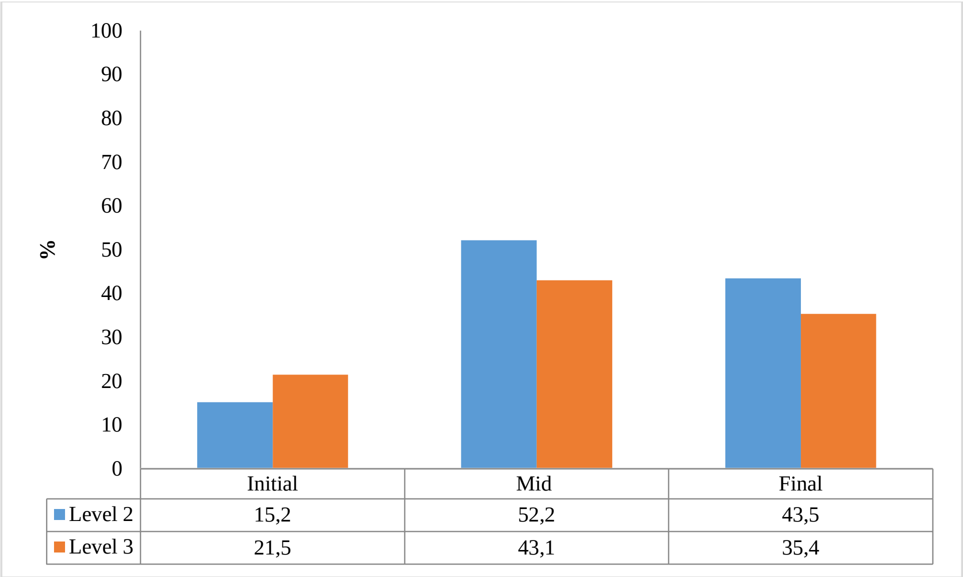

Supplementary Figure 19. Stages of quartz core abandonment.

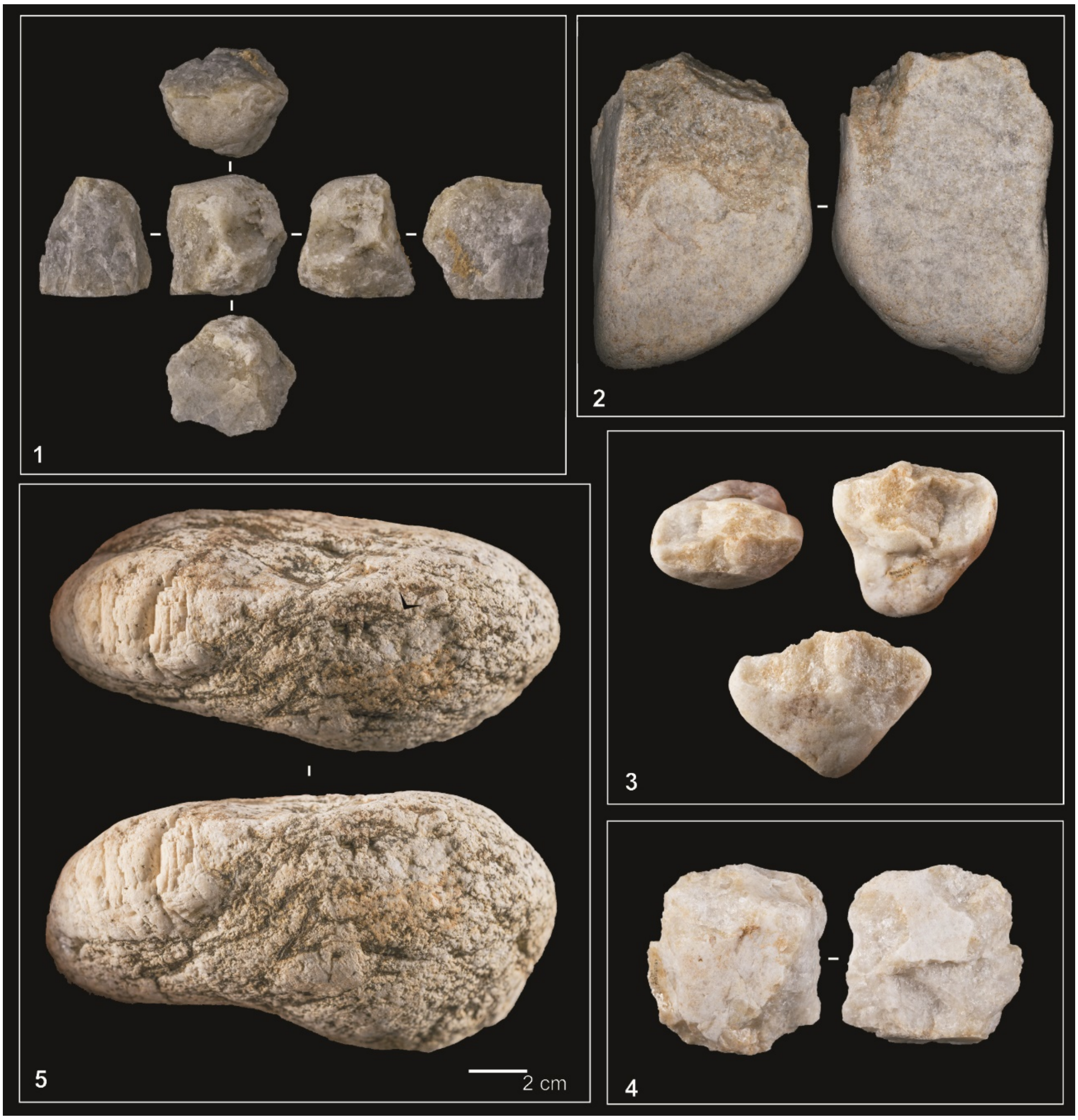

Supplementary Figure 20. Cores and a hammerstone. Levels 2 and 3, Cueva Des-Cubierta. Black arrow on Fig. E3.5 points to battering marks. Photo credit: Alfonso Dávila (1, 3, 4 and 5) and Javier Trueba/MSF (2).

1. Trifacial quartz core. Bipolar knapping on an anvil (17/26/CDC/F’48/5/28) (Level 2).
2. Bifacial quartz core. Orthogonal bipolar knapping (10/19/CDC/J’42/1/21) (Level 3).
3. Bifacial quartz core in the *initial stage* of the *exploitation (*18/29/CDC/K’38/101/72) (Level 3).
4. Bifacial quartz multidirectional discoid core (18/29/CDC/K’36/101/2) (Level 3).
5. Hammerstone on gneiss (18/29/CDC/G’45/101/18) (Level 3).

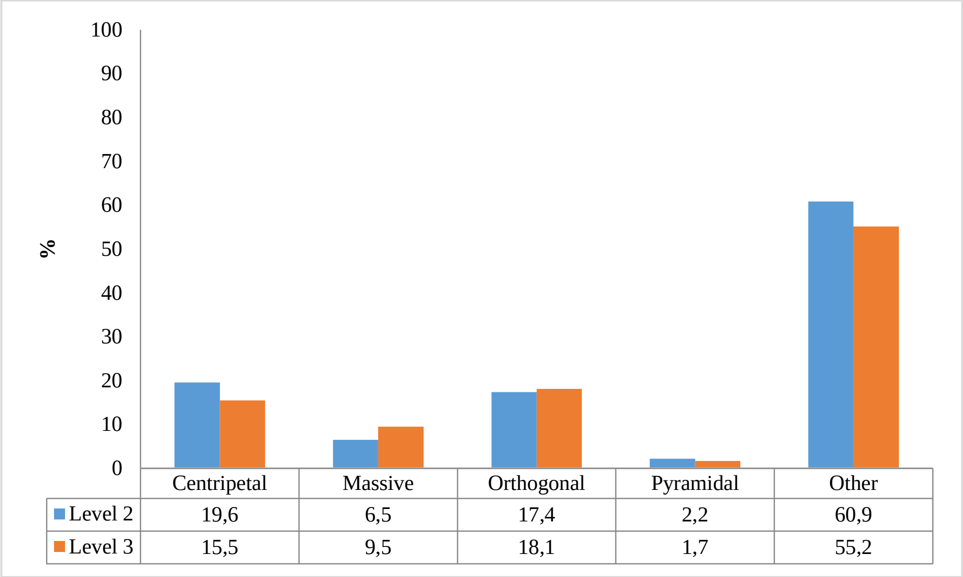

	Centripetal	Massive	Orthogonal	Pyramidal	Other
Level 2	19,6	6,5	17,4	2,2	60,9
Level 3	15,5	9,5	18,1	1,7	55,2

Supplementary Figure 21. Quartz cores. Main knapping methods.

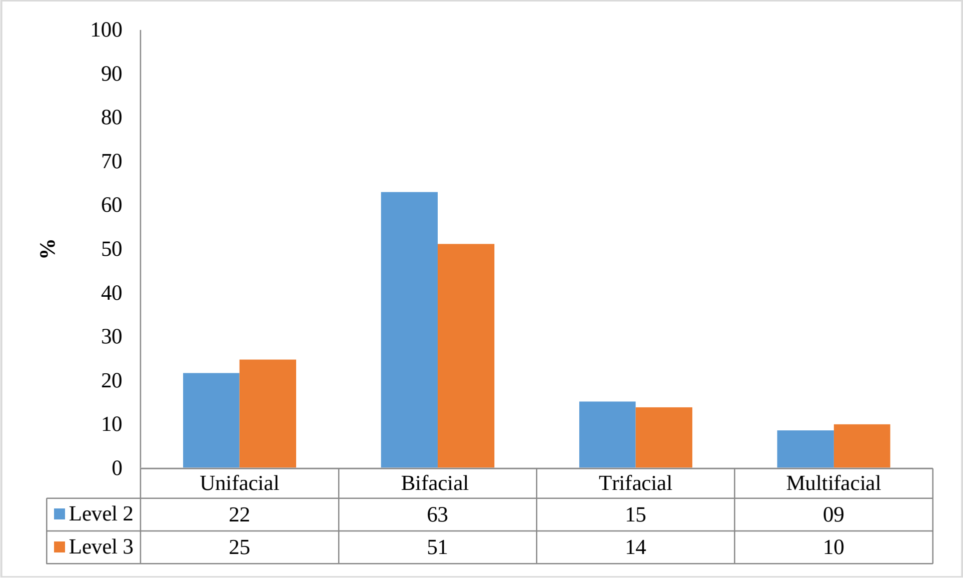

	Unifacial	Bifacial	Trifacial	Multifacial
Level 2	22	63	15	09
Level 3	25	51	14	10

Supplementary Figure 22. Quartz cores. Knapping techniques.

The re-use of broken hammerstones as cores was common (Extended Data Fig. 2). The cores were generally knapped in an expeditious manner, taking advantage of the special features of each blank. The dominant knapping methods used were centripetal and orthogonal, both in a bifacial and unifacial manner (Extended Data Fig. 1 and 2, and Supplementary Figure 20). Some 10% of the sample was made up of shaped tools, with denticulates and notches the most common, followed by sidescrapers and retouched flakes (Extended Data Fig. 1 and Supplementary Figure 20). Most of the anvils (Extended Data Fig. 2) and larger hammerstones (Supplementary Figure 20.5) were found in Level

3, most commonly associated with large mammal crania (see Figs. 1 and 2 of the main text). The hammerstones from Level 2, were slightly lighter (371.3 g) than those from Level 3 (409.6 g). Impact fractures and signs of post-hammering battering are common (Extended Data Fig. 2). In contrast, most of the anvils (of metamorphic origin) show only a few such marks, although this is largely due to their poor state of preservation.

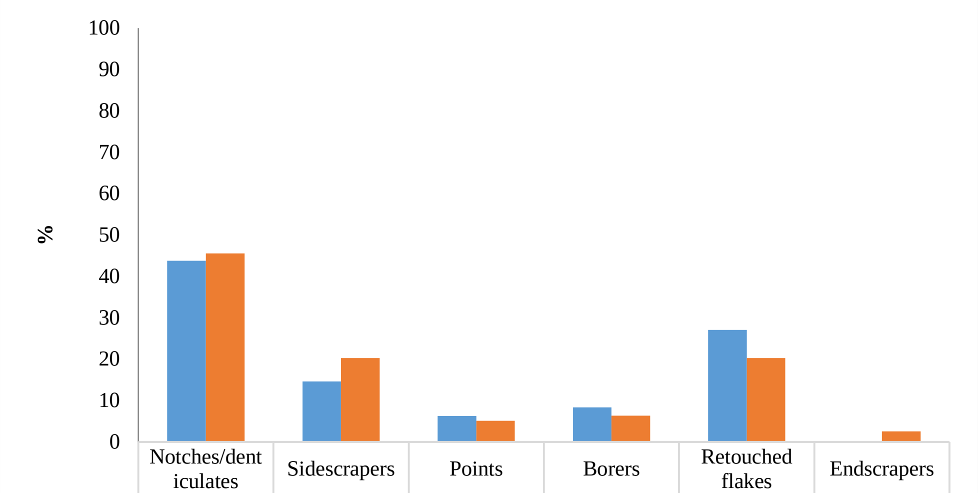

	Notches/denticulates	Sidescrapers	Points	Borers	Retouched flakes	Endscrapers
Level 2	43,8	14,6	6,3	8,3	27,1	
Level 3	45,6	20,3	5,1	6,3	20,3	2,5

Supplementary Figure 23. Categories of shaped quartz tools.

Simple flakes were the most common items (49.7% in Level 2, 50.4% in Level 3), with nearly half showing some cortex (48.2% of those from Level 2, and 50.9% of those from Level 3). Very few lithic items showed traits of heat alteration (1.3% for Level 2, 0.9% for Level 3).

The features of the lithic samples from the Cueva Des-Cubierta cave (prior to use-wear analysis) afford an initial understanding of the activities carried out at the site. The large number of hammerstones used as retouchers (10.2% in Level 2 and 13.9% in Level 3), and cores (9% in Level 2 and 11.7% in Level 3), bear witness to much knapping activity. The existence of larger hammerstones, along with anvils commonly associated with the large mammal crania mentioned above, suggests they may have been used in the latters' processing.

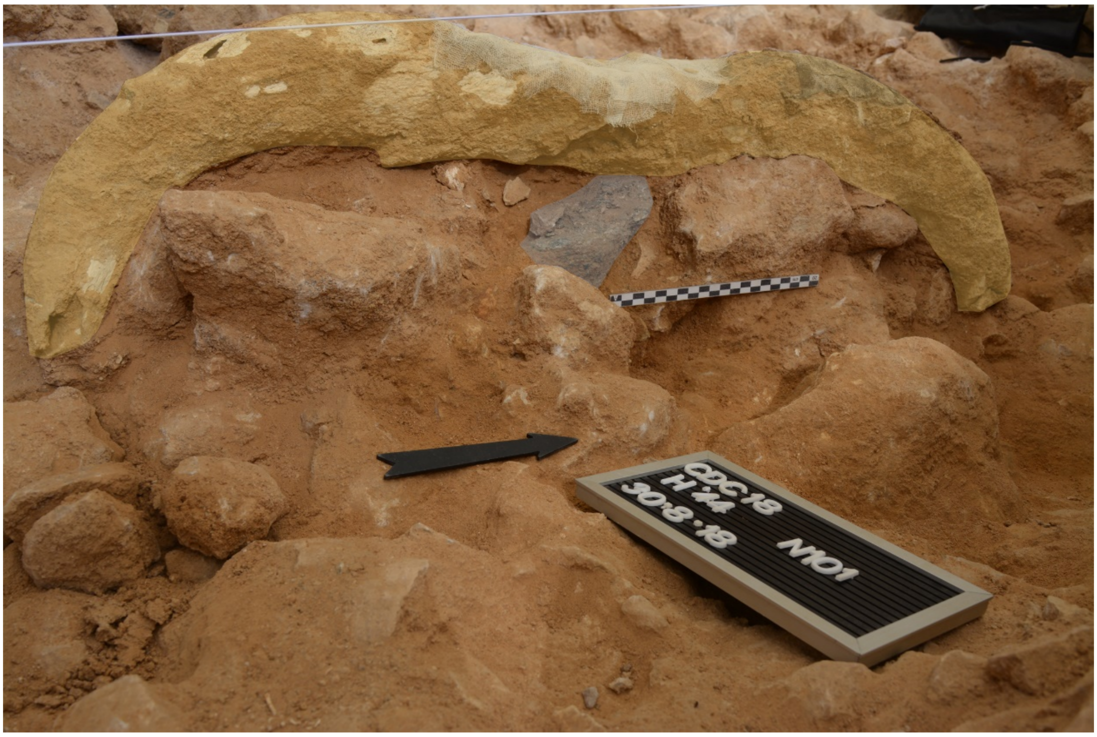

Supplementary Figure 24. Photograph corresponding to Figure 1 of the main text. The aurochs cranium has been highlighted with a yellowish shading, and the anvil under the cranium with greyish shading.

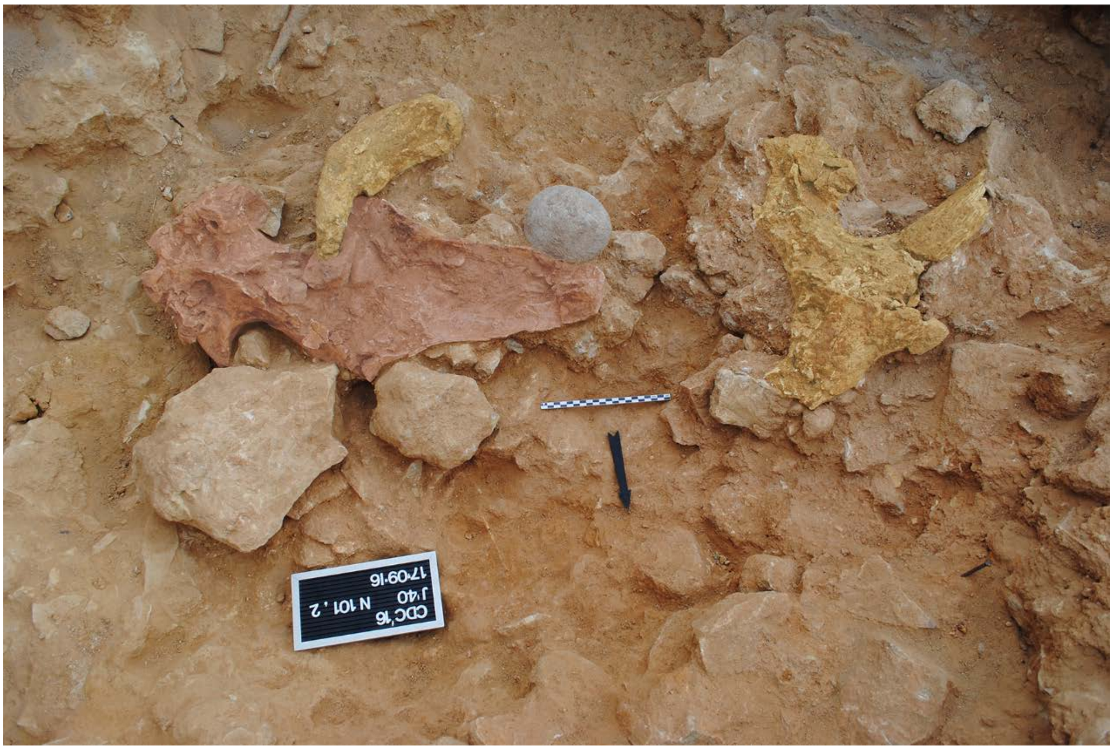

Supplementary Figure 25. Photograph corresponding to Figure 2 of the main text. The rhinoceros cranium has been highlighted with a reddish shading, the bovine cranial remains with a yellowish shading, and the granite hammerstone with a greyish shading.

Evidence of Fire

Fire-induced thermoalteration of bone remains, dolomitic clasts, lithic industry, charcoals and sedimentary matrix was evident. Nearly 38% of the archaeological remains from Level 2 had been affected by fire, as had 34.0% of those from Level 3 (Supplementary Tables 13 and 14).

Supplementary Table 13. Evidence of fire damage on archaeological remains (Levels 2 and 3). The right columns for each type of remain shows the number of remains (NR) and associated percentage representation (%).

	Bone remains		Clast remains		Lithic industry		Charcoals
	Remains NR	Burned remains NR (%)	Remains NR	Burned remains NR (%)	Remains NR	Burned remains NR (%)	Burned remains NR
Level 2	1388	62 (3.98)	2369	307 (12.95)	778	14 (1.79)	915
Level 3	4923	1502 (30.50)	5439	710 (13.05)	1442	16 (1.1)	338
Total	6304	1564	7808	1017	2220	30	1253

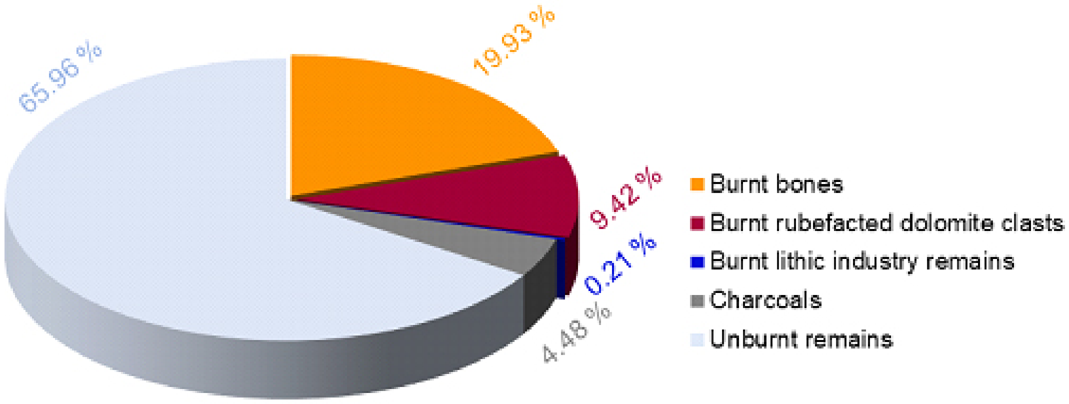

Supplementary Figure 26. Level 3: evidence of fire damage on archaeological remains.

The colour change suffered by some of the dolomitic clasts provided good evidence of thermoalteration; 307 (12.9%) affected clasts were documented for Level 2, and 710 (13.0%) for Level 3 (Supplementary Table 13 and Figures 26-28). The affected clasts from Level 2 were predominantly under 10 cm along the longest axis (NR = 225, 71.9 %)(Supplementary Table 14). The affected rubefacted clasts of Level 3 were up to 20 cm along the longest axis (NR = 650, 91.4%) (Supplementary Table 15).

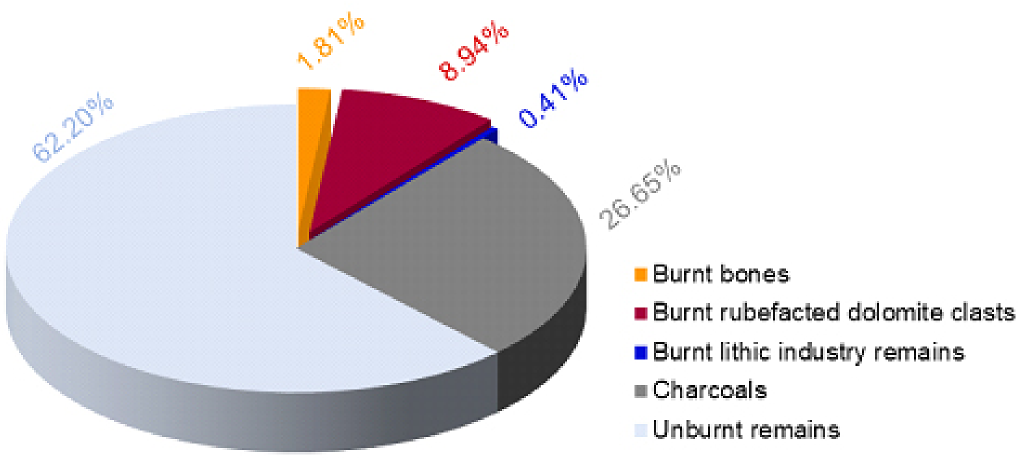

Supplementary Figure 27. Level 2: evidence of fire damage on archaeological remains.

Supplementary Table 14. Level 2: number of dolomitic burned clast remains (NR) by length (cm).

Length (cm)	Burned clast remains (NR)
<10 cm	**225**
10-20 cm	84
20-30 cm	4
30-40 cm	0
>40 cm	0
**Total**	**313**

Few lithic industry pieces showed signs of thermoalteration. Indeed, quartz (the main primary material present, followed by flint, quartzite and porphyry) may not always show evidence of thermal treatment[112]. Only 14 (1.8%, NR = 778) thermally affected pieces were identified from Level 2, and only 16 (1.1%, NR = 1442) from Level 3 (Supplementary Table 13).

In general, the lithic tools found were likely burnt after their final configuration; only one piece was affected by fire before it was knapped.

At Level 2 the charcoal fragments are more abundant (NR = 915), larger and better preserved than at Level 3 (NR = 338). These differences may be due to post-depositional processes (Supplementary Table 13).

Supplementary Table 15. Level 3: number of dolomitic burned clast remains (NR) by length (cm).

Lenght (cm)	Burned remains (NR)
<10 cm	**396**
10-20 cm	254
20-30 cm	49
30-40 cm	7
>40 cm	4
**Total**	**710**

Bone remains were the most common burnt elements, making up 30.50% of all faunal remains from Level 3 (NR_{burnt} = 1502 out of a total 4923) (Supplementary Table 13), but only 4.0% of Level 2 (NR = 62 out of a total of 1388) (Supplementary Table 13). The most common colour change among those of Level 2 and 3 was Grade 3 (carbonization; 75.5% and 68.3% respectively) (Supplementary Tables 16 and 17). The most common fragment size was 2-5 cm for both Levels (60.4% for Level 2 and 69.67% for Level 3) (Supplementary Tables 18 and 19). The presence of tiny, completely burnt remains, including in the fragmentation faces, plus the high degree of fragmentation, may indicate that the original bones were subject to high temperatures after becoming fragmented.

The cranial and post-cranial parts of the skeleton were thermoaltered in similar proportion (with slightly more cranial elements so altered) (Supplementary Table 20 and Figures 29 and 30). At least four crania thought to be trophies (three from bovines and one from a cervid) were fire-affected.

The characteristics of the sedimentary matrix of Level 3, and the post-depositional process that took place, would have greatly hindered the conservation of any combustion structures, making their identification difficult. However, the spatial distribution results revealed the presence of areas and points of combustion where the thermoaltered archaeological remains were concentrated at different depths.

In the southern part of the cave, an area of greater temperatures and fragmentation was identified. In addition, a speleothem soil at the base of Level 3 showing signs of direct burning clearly indicates combustion occurred inside the cave (Extended Data Fig. 3).

Supplementary Table 16. Level 3: burned bones classified by colour change (including double colorations). The right column shows the number of remains (NR) and associated percentage representation (%).

Colour change scale	NR (%)
Grade 1	9(0.98))
Grade 2	52(5.69)
Grade 3	**624(68.34)**
Grade 4	44(4.49)
Grade 5	3(0.32)
**Double coloration**	
Grade 1+2	0
Grade 1+3	2(0.21)
Grade 1+2+3	0
Grade 1+2+3+4	0
Grade 1+3+5	1(0.1)
Grade 2+3	28(3.06)
Grade 2+3+4	1(0.1)
Grade 2+3+4+5	2(0.21)
Grade 2+4	1(0.1)
Grade 3+4	88(9.63)
Grade 3+5	21(2.30)
Grade 3+4+5	22(2.40)
Grade 4+5	18(1.97)
Total	**913**

Supplementary Table 17. Level 2: burned bones classified by colour change (including double colorations). The right column shows the number of remains (NR).

Colour change scale	NR (%)
Grade 1	1(2.22)
Grade 2	1(2.22)
Grade 3	**34(75.5)**
Grade 4	3(6.66)
Grade 5	0
**Double coloration**	
Grade 1+2	0
Grade 1+3	0
Grade 1+2+3	0
Grade 1+2+3+4	0
Grade 1+3+5	0
Grade 2+3	3(6.66)
Grade 2+3+4	0
Grade 2+3+4+5	0
Grade 2+4	0
Grade 3+4	1(2.22)
Grade 3+5	1(2.22)

Grade 3+4+5	0
Grade 4+5	1(2.22)
Total	**45**

Supplementary Table 18. Level 3: number of remains (NR) of burned bones by length (cm).

**Length (cm)**	**Burned remains (NR)**
<2 cm	285
2-5 cm	**835**
5-10 cm	79
>10 cm	21
**Total**	**1220**

Supplementary Table 19. Level 2: number of remains (NR) and associated percentage representation (%), of burned bones by length (cm).

**Length (cm)**	**Burned remains (NR)**
<2 cm	17
2-5 cm	**31**
5-10 cm	6
>10 cm	1
**Total**	**55**

Supplementary Table 20. Levels 2 and 3: burnt bones classified by anatomical position (cranial/post-cranial). The right columns show the number of remains (NR) and associated percentage representation (%).

	**Faunal remains**			
	Cranial remains NR	Burned cranial remains NR (%)	Post-cranial remains NR	Burned post-cranial remains NR (%)
Level 2	139	**8 (5.75)**	852	40 (4.69)
Level 3	2759	**678 (24.57)**	1149	235 (20.45)
Total	2898	685	2001	275

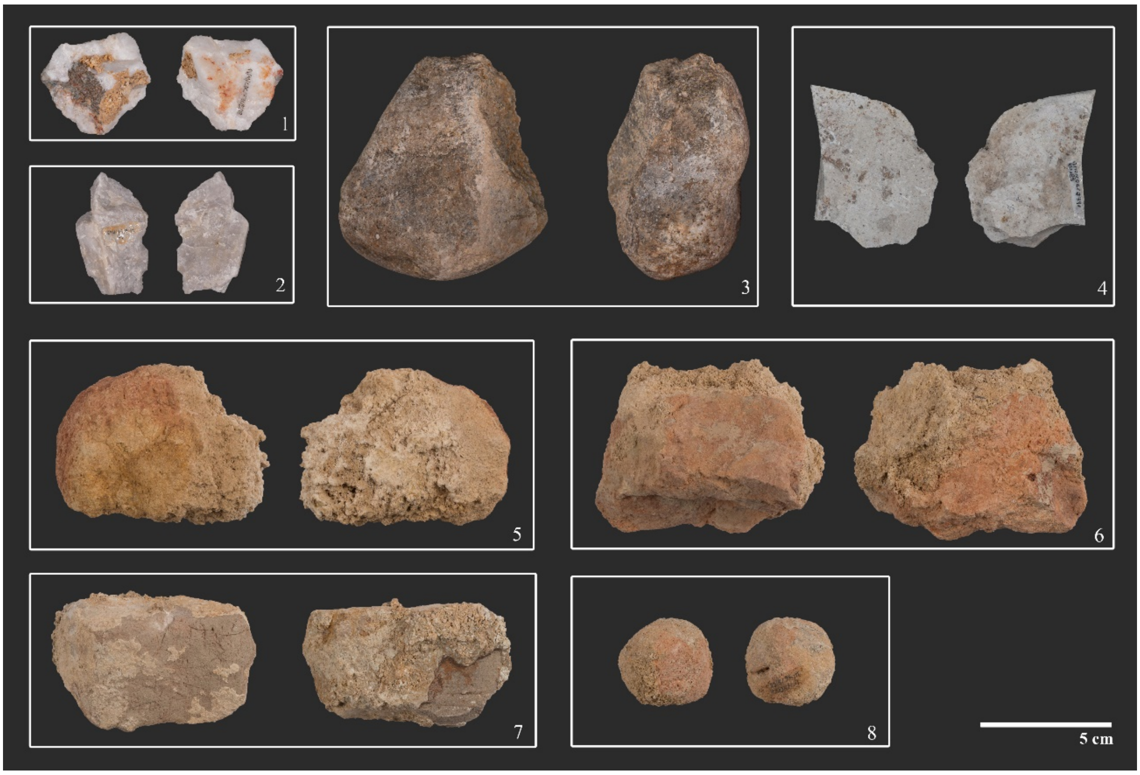

Supplementary Figure 28. Burnt lithic industry and burnt/rubefacted dolomite clasts (Levels 2 and 3).

Burnt lithic industry: **1.** Burnt quartz core (14/11/CDC/F´47/5/18). **2.** Burnt quartz fragment (15/13/CDC/H´45/5.5/84). **3.** Broken, burnt quartzite hammerstone (14/11/CDC/I´40/101/6). **4.** Burnt chert flake with thermal potliding (13/10/CDC/J´39/101/85).

Burnt rubefacted dolomitic clasts: **5.** Redish rubefacted limestone block (15/13/CDC/K'39/101/3). **6.** Redish rubefacted limestone block (14/11/H'45/5.5/104). **7.** Grey rubefacted limestone block (14/11/H'45/5.5/47). **8.** Redish rubefacted limestone block (16/12/H'46/5/66).

Photo credit: Alfonso Dávila.

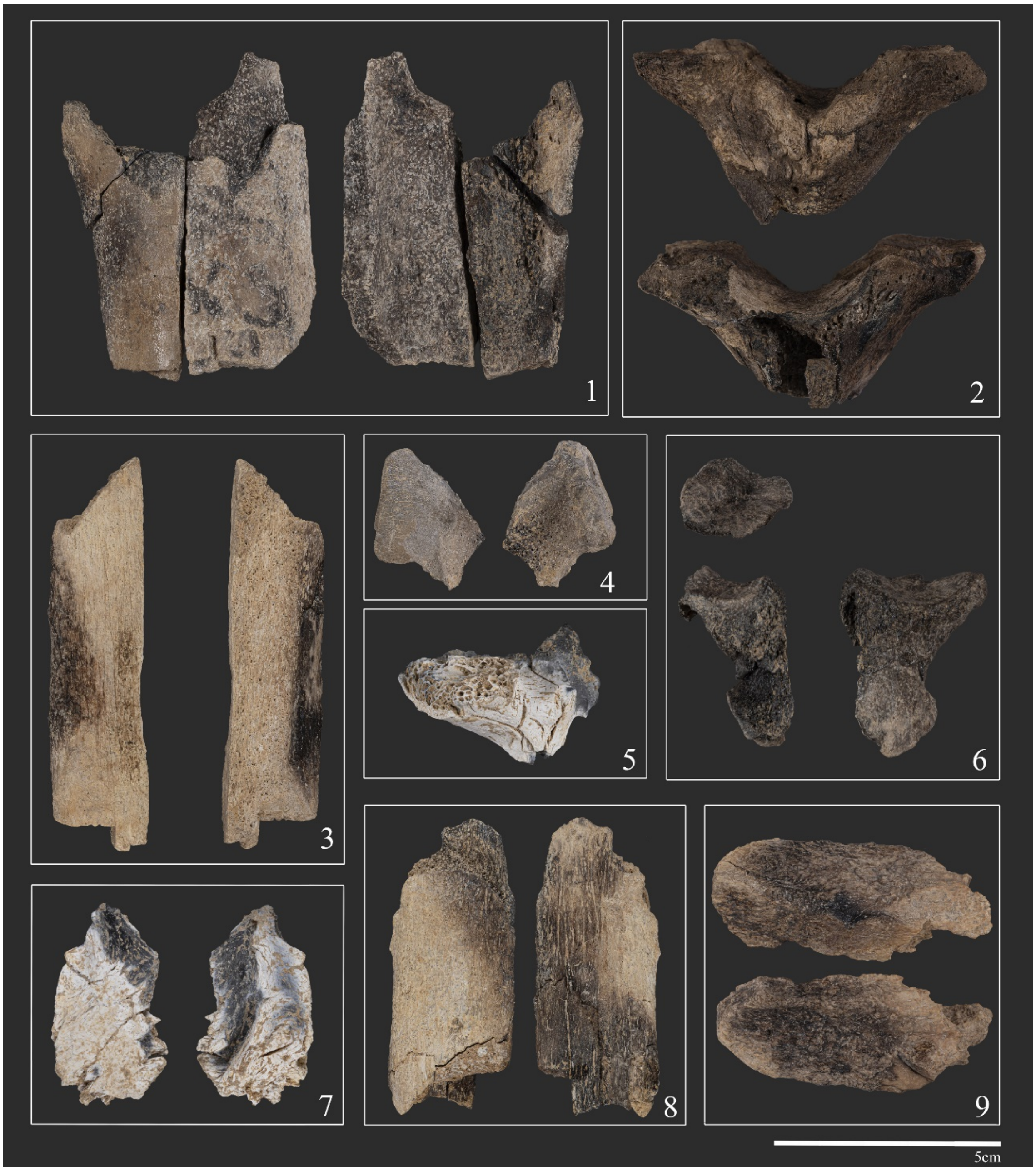

Supplementary Figure 29. Post-cranial burnt bones from Level 3, Cueva Des-Cubierta cave. The burning damage described here is as defined by [117,118]. **1**. Long bone diaphysis portion (15/13/CDC/J'38/101/143); grades 2+3. **2.** Vertebra portion (15/13/CDC/G'41/1/28); grades 2+3. **3**. Rib portion (11/13/CDC/G'42/100/1); grades 2+3. **4.** Long bone diaphysis portion (16/12/CDC/J'38/101/13); grades 2+3+4. **5.** Indeterminate post-cranial bone portion (14/11/CDC/J'37/101/80); grade 4. **6.** Second phalanx portion (16/12/CDC/J'39/101/49); grades 3. **7.** Indeterminate post-cranial bone portion (19/34/CDC/J'39/101/23); grades 3+5. **8**. Long bone diaphysis portion (11/13/CDC/G'42/1/25); grades 2+3. **9.** Indeterminate post-cranial bone portion (11/13/CDC/I'40/1/25); grades 2+3. Photo credit: Alfonso Dávila.

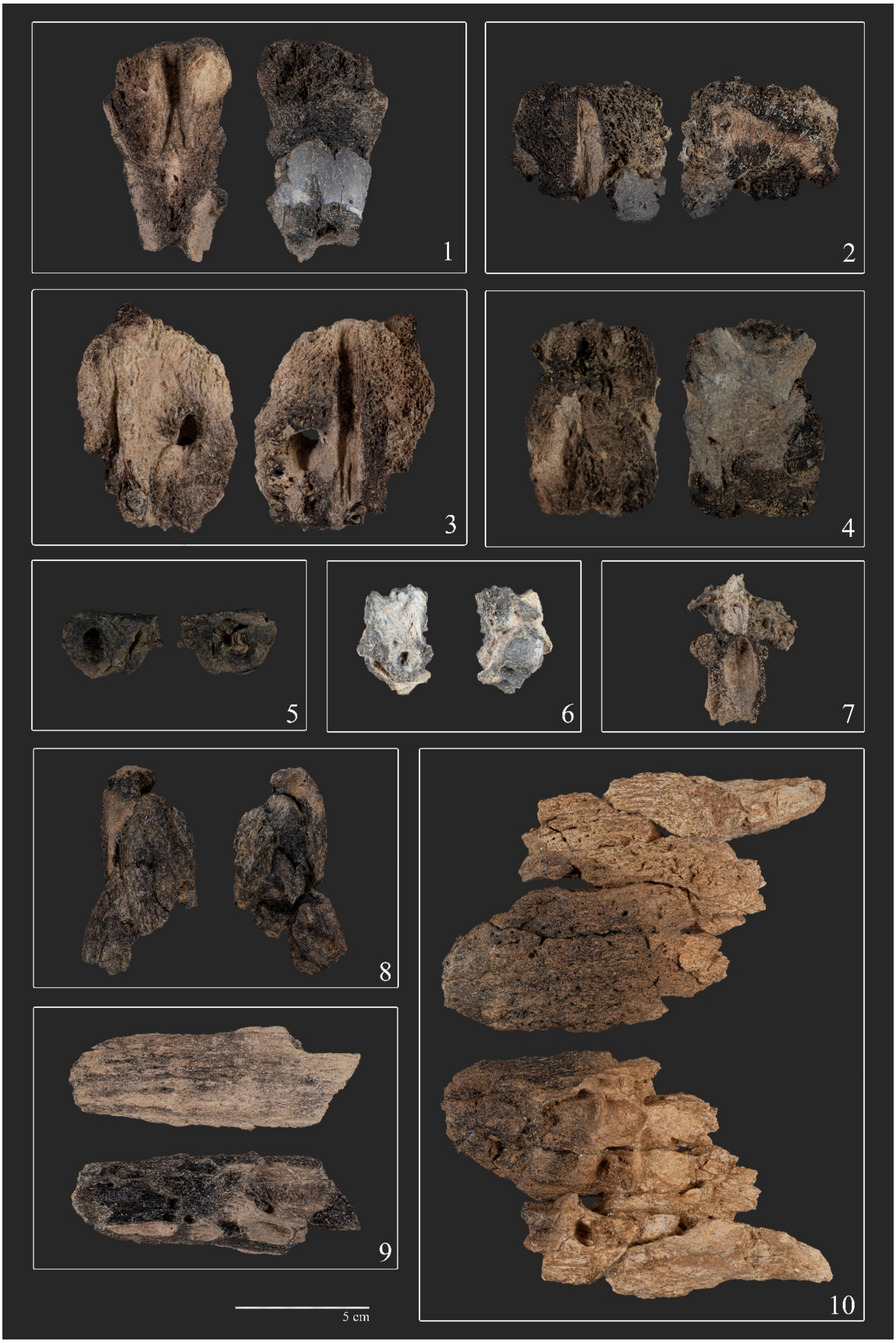
1
2
3
4
5
6
7
8
9
5 cm
10

Supplementary Figure 30. Cranial bones of large bovines from Level 3, Cueva Des-Cubierta. The burning damage described here is as defined by [117,118]. **1.** Basilar portion of the occipital bone (12/9/CDC/J’37/101/41); grades 3+4. **2**. Cranial portion (12/9/CDC/J’37/101/27); grades 2+3+4. **3.** Cranial portion (15/13/CDC/I’36/101/1); grades 2+3. **4.** Basilar portion of the occipital bone (14/11/CDC/I’36/251/2); grades 2+3+4. **5**. Petrous bone (14/11/CDC/I’36/251/18); grade 3. **6.** Cranial portion (14/11/CDC/I’36/251/20); grade 4. **7.** Cranial portion (14/11/CDC/I’36/251/3); grades 2+3+4. **8.** Apical portion of horn core (16/12/K’39/101/169); grades 2+3. **9.** Horn core portion (16/12/J’38/101/1); grades 2+3. **10.** Basal portion of horn core (16/12/K’39/101/100g); grades 2+3. Photo credit: Alfonso Dávila.

Presence of hearths

During the excavation of Level 3, several concentrations of burnt bones, charcoal, rubefacted carbonate rocks and thermally altered lithic industry were found, which are here proposed to correspond to hearths. However, the type of sediment in which they were found (formed mainly by large clasts with a small amount of quickly friable matrix) can leave them difficult to appreciate and excavate.

The most evident hearth was found in square J'36 over speleothem S1. Several rubefacted carbonate rocks (with reddish and greyish colours) were located in this square, spatially associated with concentrations of burnt bones and charcoal, and with a bison horn showing evidence of thermal alteration. The heat produced by the hearth caused a colour change in the underlying speleothem, which shows a dark spot in the area close to the concentration of heat-altered elements. According to preliminary experimental results, carbonate clasts acquire reddish tones after direct exposure to fire, and greyish when buried in the vicinity of a heat source (where the environment would lack oxygen), and also for other reasons. Supplementary Figures 31 to 39 provide graphic documentation of the excavation of this square, where all the elements mentioned above are recognizable.

This evidence is sufficient to justify the presence of hearths inside Level 3. One of the present authors is currently undertaking a doctoral thesis on the characteristics of Neanderthal behaviour, investigating the pyrotechnology of the Cueva Des-Cubierta cave. This thesis seeks to recognize structures or combustion zones through microcontextual (micromorphological analysis) and molecular methods (extraction of lipid markers). Another author is conducting her doctoral thesis on the spatial distribution

of the archaeological items at the site. Hopefully, these results will confirm the findings made by the research team during the excavation of the site.

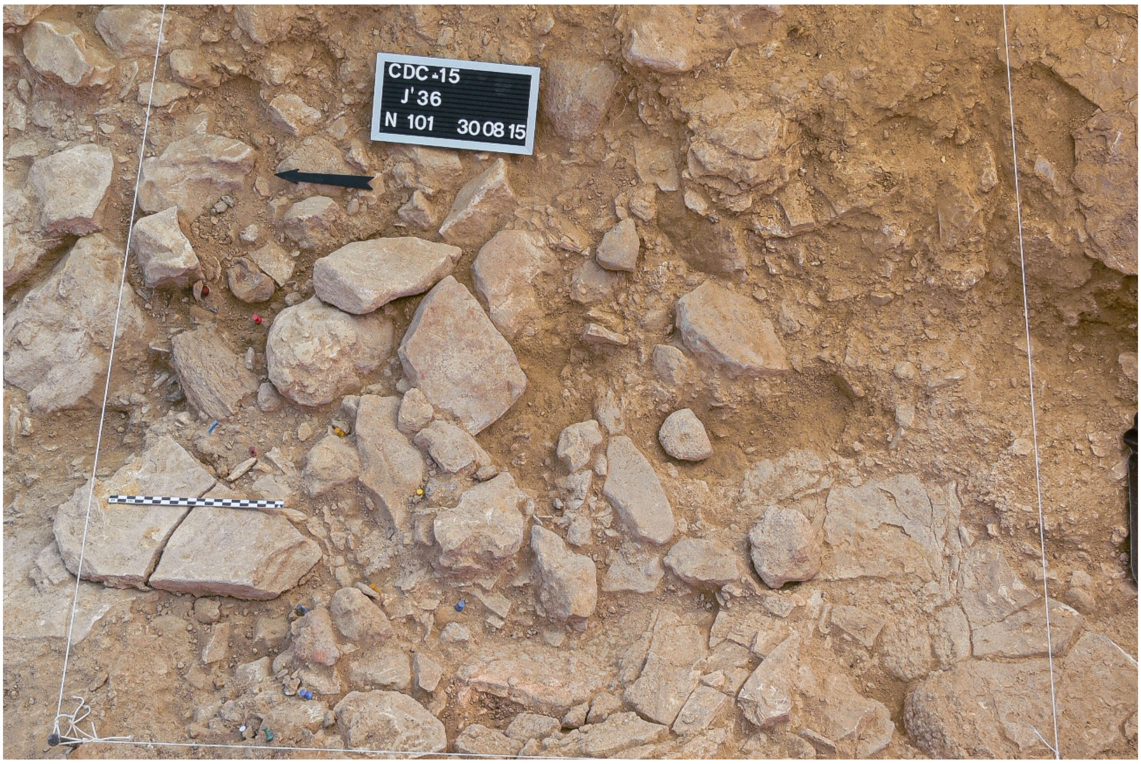

Supplementary Figure 31. Square J'36 during the 2015 excavation campaign. Black arrow points north (left side of the picture). A part of a bison horn is beginning to appear at the north end of the square. Speleothem S1 begins to be visible in the western part of the square.

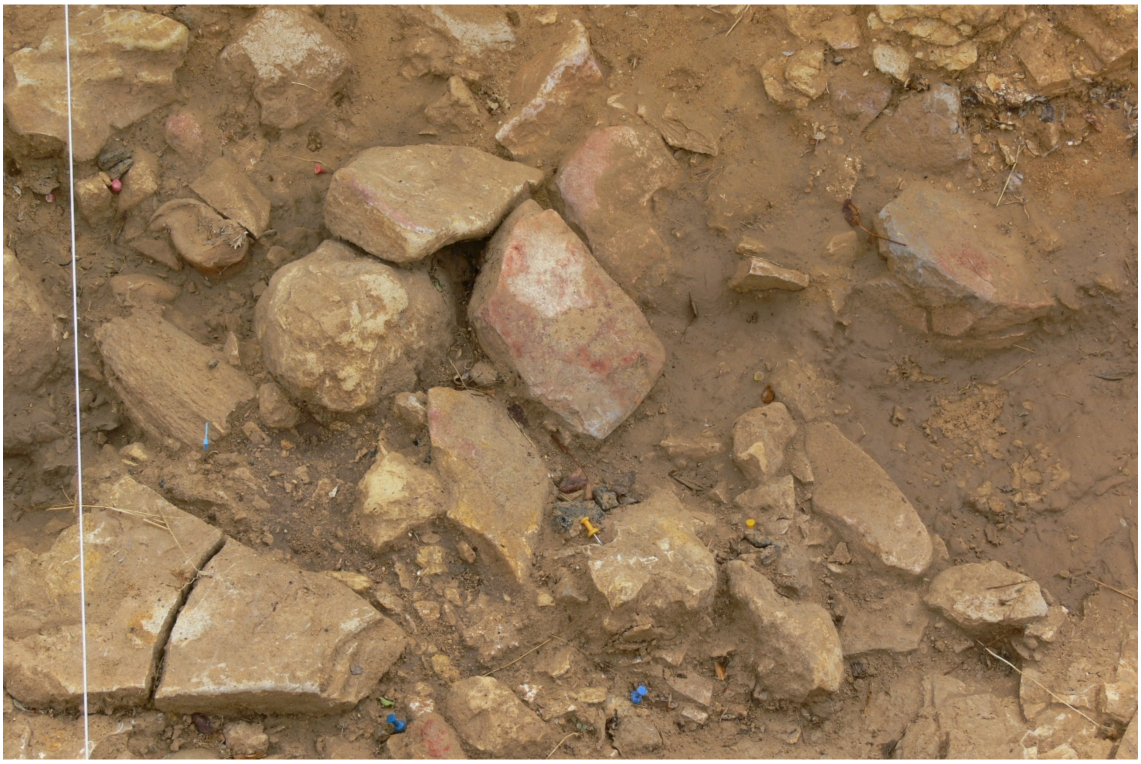

Supplementary Figure 32. Detail of square J'36 after a summer storm and partial site flooding. The wetting of the clasts highlights the reddish and greyish colours due to rubefaction.

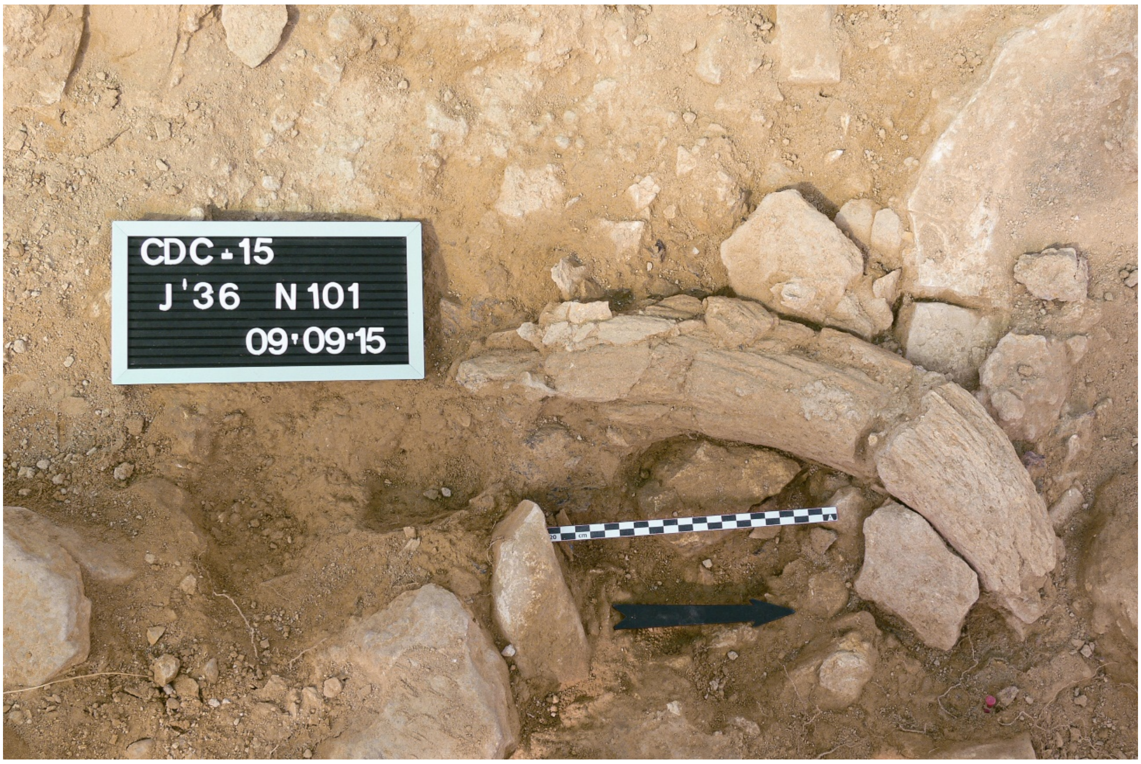

Supplementary Figure 33. Detail of the same square some days later (the most advanced stage of excavation). The bison horn is now fully visible (note that north is now to the right of the image).

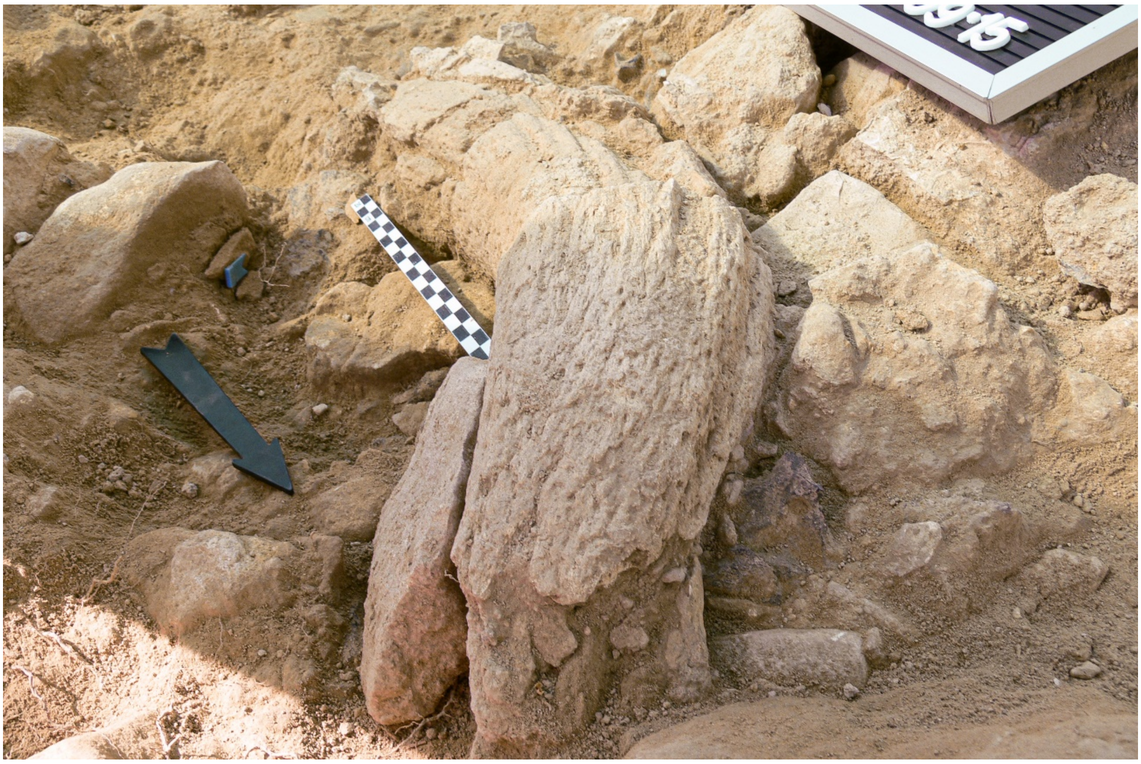

Supplementary Figure 34. Detail of the apical region of the horn. To its right, at its base, is a concentration of dark, burnt bones.

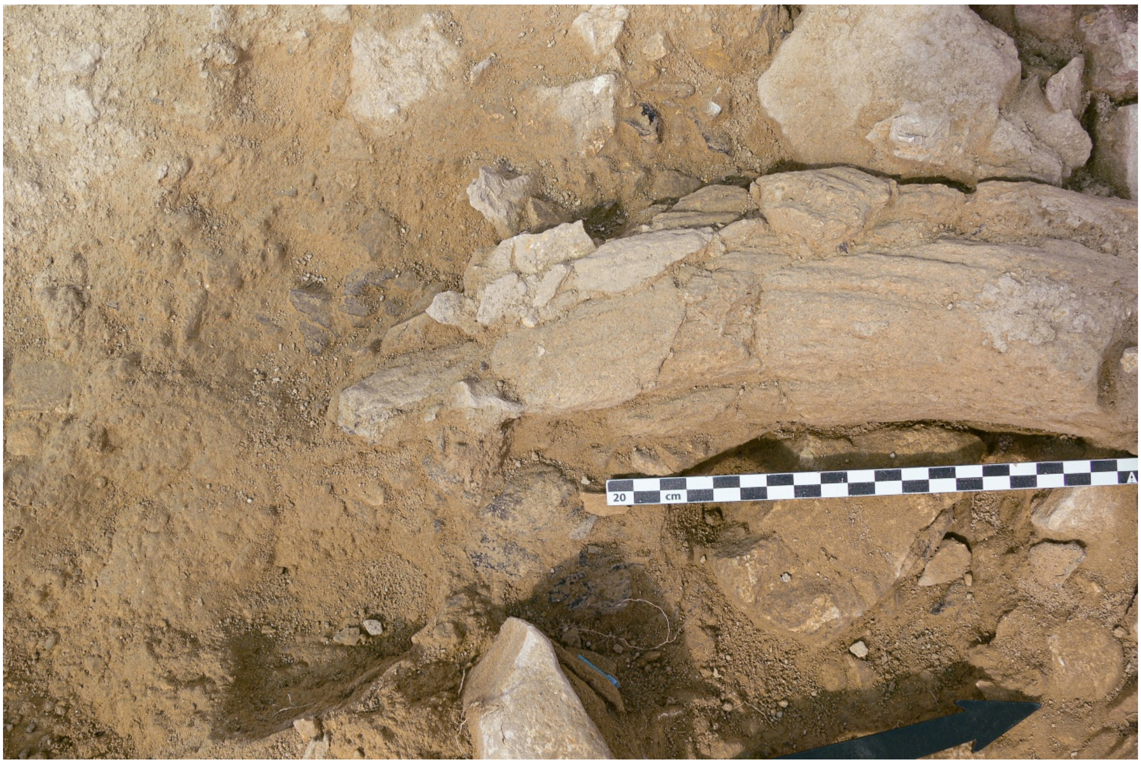

Supplementary Figure 35. Detail of the proximal region of the horn. On both sides, there are concentrations of dark-coloured burnt bones.

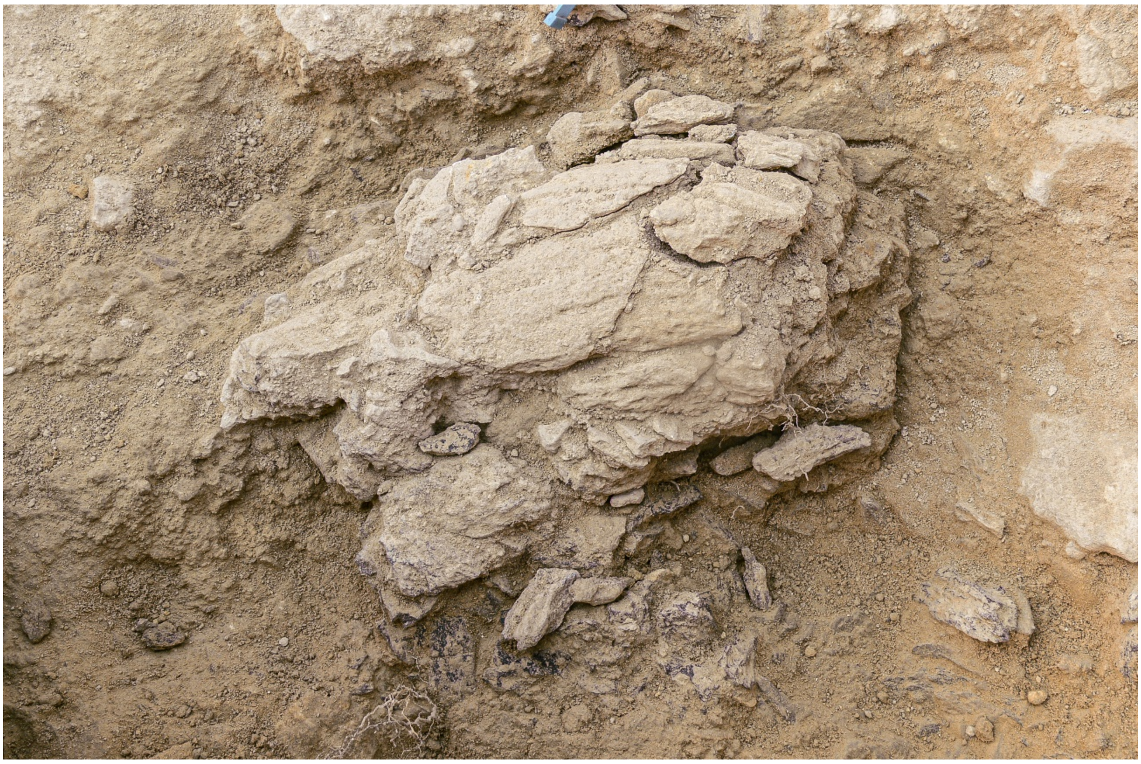

Supplementary Figure 36. Detail of the proximal region of the horn after removal of its apical region. The lower part of the image shows the concentration of burnt bones at its base.

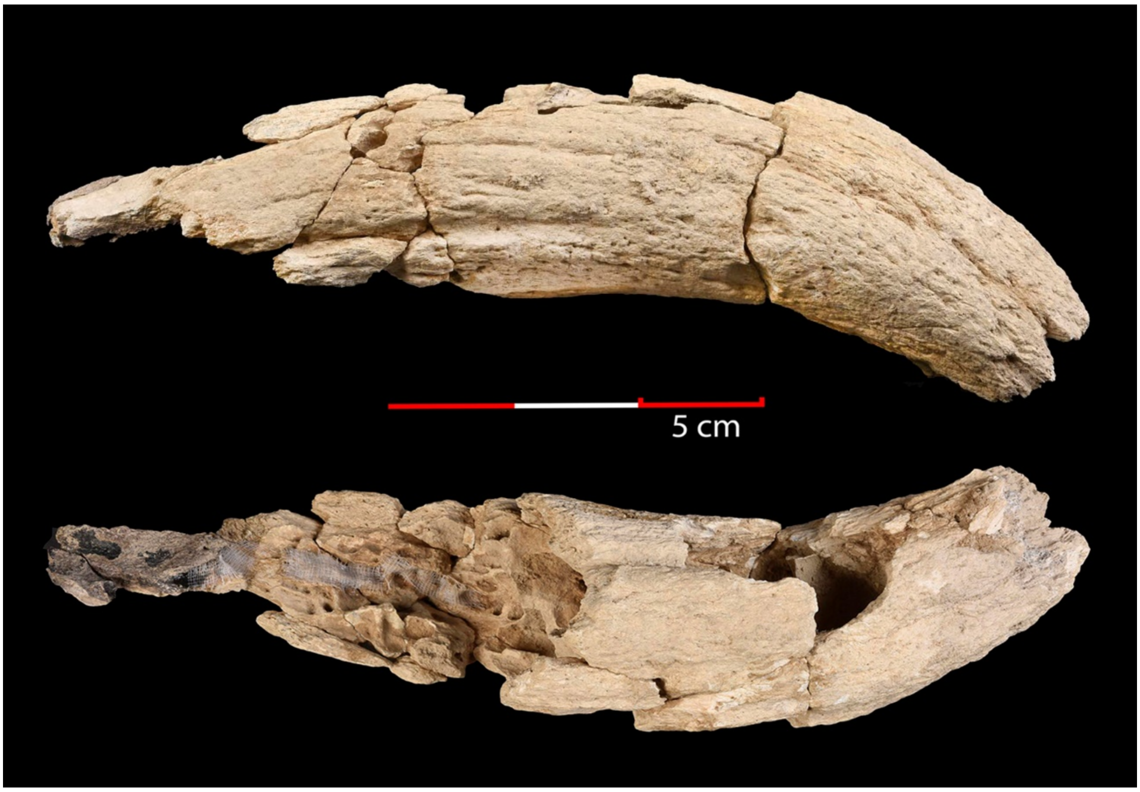

Supplementary Figure 37. The bison horn from square J'36 now restored. In the lower part (with respect to its position in which it was found during the excavation) of its proximal region, gray colorations can be seen due to its exposure to high temperatures (lower image). Photo credit: Mario Torquemada/MAPCM.

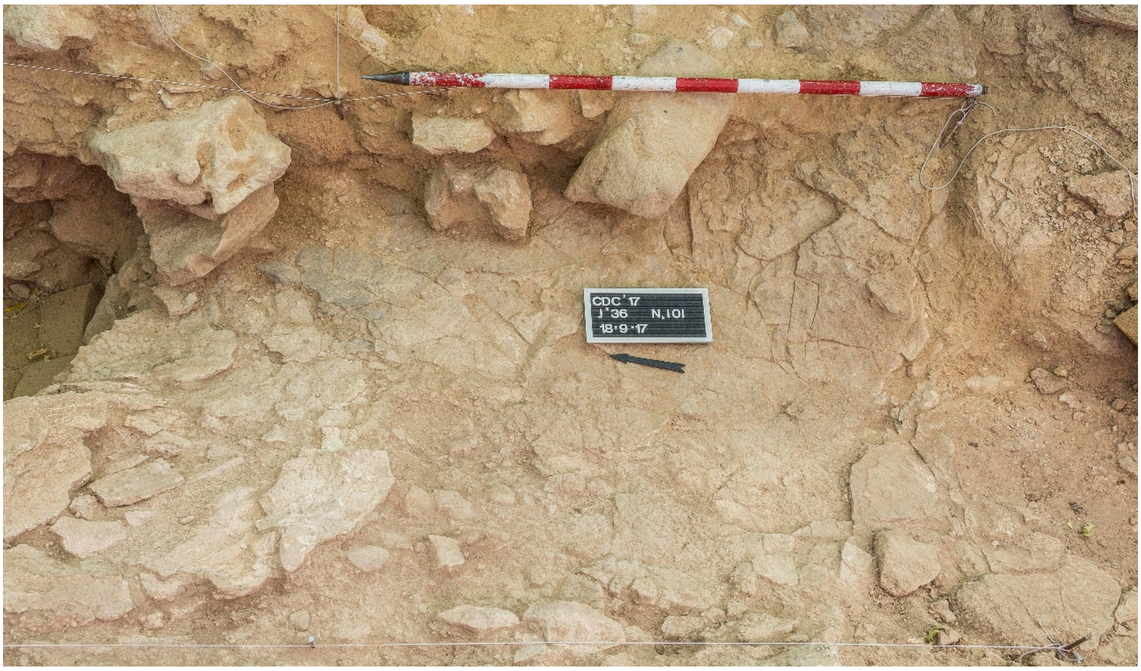

Supplementary Figure 38. Square J'36 during the 2017 excavation campaign. Speleothem S1 is visible on almost the entire surface of the square. At its eastern end, a dark spot can be seen, interpreted here to be due to its thermal alteration. Photo credit: Alfonso Dávila.

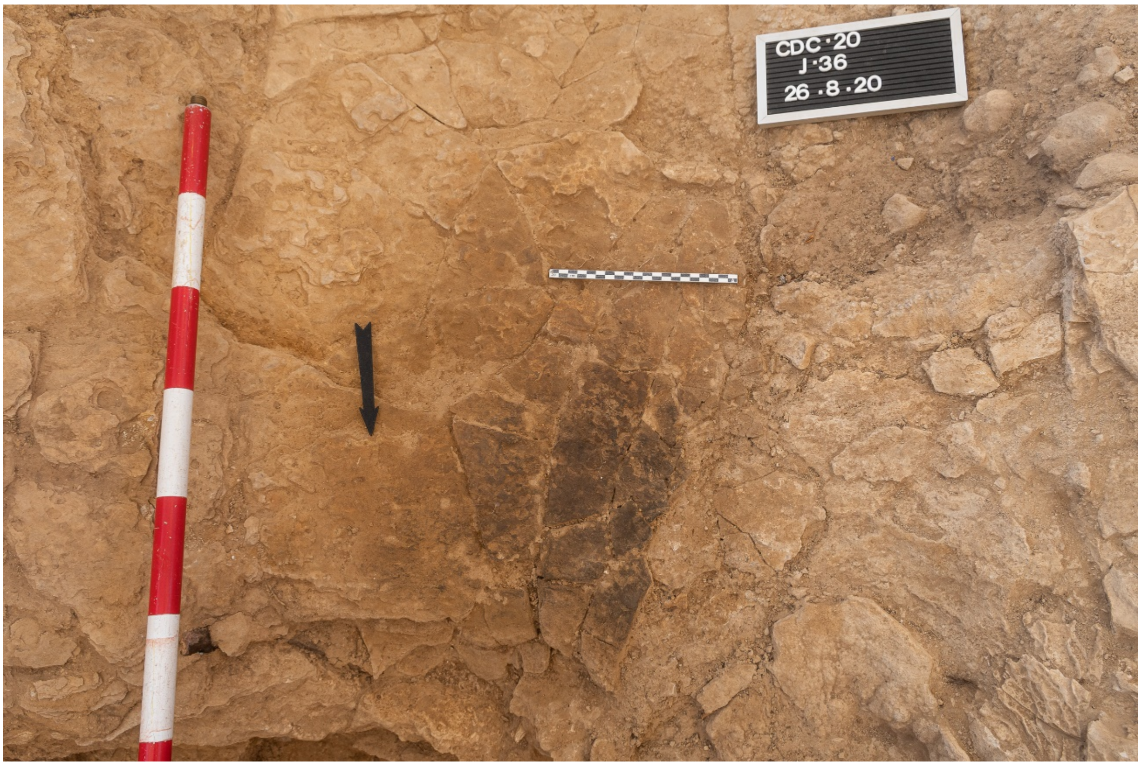

Supplementary Figure 39. Detail of the dark spot on speleothem S1 in square J'36 during the 2020 excavation campaign. The speleothem has been moistened to improve the visibility of the dark spot (note that north is now to the lower part of the image). Photo credit: Alfonso Dávila.

Taphonomy

The anatomical representation of large mammals, especially of bovines, was different for Levels 2 and 3. As indicated in the Faunal remains section of the Supplementary Results, in Level 2 nearly all the elements of the bovine skeleton are represented, although elements of the postcranial skeleton predominate by far. In Level 3, the representation of the postcranial skeleton is meagre; the most documented elements were crania.

Post-depositional modifications

Although fragmented, the assemblage is generally well preserved. However, post-depositional changes such as the presence of concretions (30.0%), oxides of manganese (10.4%), dissolutions (14.5%), and rounded (14.9%) and polished surfaces (17.7%), were present (although low grade [R1-P1], according to Cáceres[118]). These modifications indicate the existence of damp conditions with the presence of low energy runoff water typical of karst systems (Supplementary Table 21).

Supplementary Table 21. Percentage of post-depositional modifications (Level 3).

Post-depositional modification	%
Concretion	30.0
Oxides of manganese	10.4
Rounded	14.9
Polish	17.7
Dissolution	14.5

The presence of small cranium fragments reflects the notable fragmentation of these elements inside the cavity owing to post-depositional processes. The sedimentary package of Levels 3 and 2 is characterized by an abundance of clasts or blocks with little matrix between them. This composition would favour the fragmentation of any bone remains accumulating in the filling.

The abundance of bone remains with modifications due to rounding and polishing (grade 1 and 2), concretion coatings, and the yellowish coloration of the bone surfaces, together suggest the presence (although not permanent) of runoff water inside the cavity (Supplementary Table 21).

Although in general terms the recovered bones show good structural preservation (despite the clear post-depositional fractures shown by some specimens), their cortical conservation is poor. The results of the bone surface modifications analysis are therefore probably underestimates.

Supplementary Table 22. Anthropic modifications identified for the faunal remains of Level 2.

Level 2	Cut marks	Location	Activity	Anthropogenic breakage
**Rib**	1 (Sl)	Shaft	Defleshing	-
**Femur**	-			1
**Tibia**	-			1
**Long bone**	-			1
**TOTAL**	1			3

Modifications of anthropic origin

Anthropic cut marks and fracturing were both documented, and both for Level 2 and 3.

In Level 2, remains with anthropic modifications were scarce (Number of Specimens = 4, 0.4%); indeed, they were identified on just three long bones and one flat bone, all of them belonging to a large bovine. The three long bones (a tibia, a femur and an indeterminate bone) showed modifications related to percussion fracturing (conchoidal scars and flakes) to obtain the marrow. The flat bone (a rib) showed an incision, possibly made during the defleshing of the animal (Supplementary Table 22).

For Level 3, anthropic activity was identified on 36 remains (1.6% of the total). The most abundant modification was anthropic fracturing (NSP = 32); cut marks were very rare (NSP = 8). Four of the recovered remains showed both types of modification (Supplementary Table 23).

Most of these modifications were identified on postcranial skeletal remains (mainly the appendicular section) of large adult animals (Supplementary Figure 40). However, modifications of anthropic origin were also seen on elements of medium-sized animals, e.g., a humerus of *Cervus elaphus* that showed anthropic fracturing (Supplementary Tables 23 and 24).

In Level 3, two cranial remains (one from *Stephanorhinus hemitoechus*, the other from *Bison priscus*), for which the cortical bone surface was better preserved, showed clear evidence of human activity (Extended data Fig. 4 and 5). The rhinoceros cranium showed

both cut marks and fractures of anthropic origin. On the bison cranium, cut marks were identified in the nasal area (Supplementary Table 25).

Supplementary Table 23. Anthropogenic modifications identified for the faunal remains of Level 3. The columns show number of specimens (NSP) by anatomical elements and the size of the captured animals.

	Anthropic breakage			Cut marks	
	Very large-sized	Large-sized	Medium-sized	Very Large-sized	Large-sized
**Cranium**	2	-	-	1	1
**Radius**	-	1	1	-	-
**Humerus**	-	1	1	-	-
**Metacarpal**	-	1	-	-	-
**Rib**	-	1	-	-	-
**Vertebra**	-	-	-	-	1
**Femur**	-	3	-	-	-
**Tibia**	-	1	-	-	1
**Metapodial**	-	1	-	-	-
**Phalange**	-	1	-	-	-
**Long bone**	-	15	3	-	3

Supplementary Table 24. Number of specimens (NSP) showing anthropic breakage. The columns show the NSP, NSP by anatomical element, taxonomic identification, and the size of the captured animals.

		Percussion breakage
**Taxa**	**NSP**	**NSP by anatomical elements**
Stephanorhinus hemitoechus	2	2 Cranium
Bos/Bison	8	1 Rib, 2 Femur, 1 Humerus, 1 Radius, 2 Metapodial, 1 Phalange,
Bos primigenius	2	1 Femur, 1 Tibia
Cervus elaphus	1	1 Humerus
**Large size**	15	15 Long bones (indeterminate)
**Medium size**	4	1 Radius, 3 Long bones (indeterminate)

These anthropic modifications indicate that hominins processed at least two of the crania recovered from Level 3. It is important to note that the post-depositional fragmentation of most of the crania recovered from the site renders it impossible to identify any anthropic fracturing, should it have occurred.

Supplementary Table 25. Remains with cut marks, the number of cut-marks, location, morphology, and activity deduced by element, taxon and size category. Cm: Cut marks, Sl: Slice marks, Ch: Chop marks, Sk: Skinning, Df: Defleshing, Da: Disarticulation.

Element	Taxa or Size weight category	Nº Cm	Location	Cm-m	Activity
Cranium	*Stephanorinus hemitoechus*	17	Zygomatic, Basioccipital, Nasal	Sl	Sk+Df
	Bison priscus	11	Nasal	Sl	Sk+Df
Tibia	*Bos primigenius*	1	Distal shaft	Ch	Da
Vertebra	Bos/bison	4	Spinnous process	Sl	Df
Long bone (indeterminate)	Large size	4	Shaft	Sl	Df
	Large size	1	Shaft	Sl	Df
	Large size	2	Shaft	Sl	Df

Carnivore activity

The remains recovered from Levels 2 and 3 suggest that the activity of carnivores inside the cavity was practically non-existent. The associated modifications identified included only the rounding and collapse of some of the fracture edges of the bones, and the presence of small grooves and depressions. These reflect the nibbling or furrowing activity of a small carnivore with little modifying capacity.

Supplementary Table 26. Number of specimens (NSP) with carnivore tooth marks by anatomical element and size of the captured animals.

.

	Carnivore Tooth marks	
	Large-sized	Medium-sized
Rib	1(1)	1(1)
Vertebra	1(1)	-
Innominate	1(1)	-
Long bone	1(1)	-

Three remains were recovered from Level 2 that showed modifications by carnivores: a rib fragment and a coxal fragment, both from large animals, and a long bone of a medium-sized animal. Five remains were recovered from Level 3 that showed similar modifications (0.2% of the total). These were mainly flat bones of the axial skeleton of large animals, possibly bovines. However, the rib of a medium-sized animal also showed modifications made by carnivores, as did a long bone fragment from a large animal. The poor definition of the marks made it impossible to identify the carnivore involved (Supplementary Table 26).

Experimental butchering of cattle heads and comparison with the Level 3 cranial remains

The experimental butchering of the cow heads showed the best way to extract the eyes was to first break and remove the maxillae, and then the zygomatic bones. The removal of the eyes may therefore explain why the crania lack these elements. The morphology of the resulting cranium is very similar to that of the bovine crania from Level 3 (Supplementary Figure 41). The general absence of maxillae and zygomatic bones in the archaeological assemblage would indicate that these bones were removed before the crania were taken into the cave.

It is more difficult to understand how Neanderthals might have accessed the brain. The assemblage is mainly composed of crania that preserve the horns, but with a poorly conserved neurocranium, the result of post-depositional fracturing. During the experimental butchering of the cow heads, the easiest way to access the brain after removing the skin and muscle was to hit the back of the occipital bone with a hammerstone. In agreement, for most of the bovine crania from Level 3, the occipital bones were incomplete or even absent, although at this assemblage they are represented with a varying degree of integrity as isolated elements separate from the rest of the neurocranium. That might be related to accessing the brain by direct percussion on the posterior part of the occipital bone.

Summary of taphonomic results

The taphonomic analysis of the faunal remains from Level 3 revealed evidence suggestive of an anthropogenic assemblage. However, the general characteristics of the assemblage are clearly uncommon.

First, the %NISP with cut and percussion marks was low. However, this is clearly related to the presence of badly preserved cortical surfaces[227], a consequence of the post-depositional processes that affected the assemblage. The cortical surfaces were thus disturbed, and many anthropic alterations of those surfaces could have been lost. The low ratio of anthropic marks cannot be thus understood as identifying an assemblage unaffected by human activity.

The %MAU values recorded prohibit the assemblage being interpreted as the result of carnivore activity. Carnivore dens (especially hyaena dens) commonly contain fragments of herbivore crania, but post-cranial bones and teeth are also commonly present[26,27,228-235]. In Level 3, however, post-cranial elements, the mandibles and maxillae were clearly underrepresented. It might still be argued, however, that the assemblage was produced by the action of other abiotic or biotic processes. Indeed, a fossil assemblage can result from animals falling victim to a natural trap. However, in such cases the resulting accumulation would include complete animals[24,236-238]. Assemblages can also be produced by the action of a watercourse (of either high or low energy). However, the %MAU values recorded again rule this out for the examined site. Crania without maxillae and teeth, and the absence of post-cranial elements, are not characteristic of water-driven accumulations[134,239-241].

The other agents that might modify bone surfaces include ungulates and micromammals (which might gnaw at the bones for minerals or other nutrients), plants, insects, and even natural disarticulation[242-249]. However none of these agents can create accumulations of bones, much less with a clear over-representation of certain anatomical elements as in Level 3.

The differential preservation of bones can also be ruled out. Differences in bone density (see [102,250,251]) may lead to the better survival of teeth, crania, mandibles, upper limb bones, intermediate limb bones and lower limb bones[252,253], but only crania are overrepresented in the present assemblage.

Indeed, the representation of skeletal parts in Level 3 of Cueva Des-Cubierta can only be explained by the activity of Neanderthals; they could accumulate bones in a manner different to other agents, producing different %MAU patterns. The results of the experimental work make it clear that these crania were introduced into the cave without the post-cranial elements, mandibles and maxillae. This is probably due to the prior consumption of the eyes, and the meat and marrow of the different bones. The consumption of the brain - if it occurred - likely took place inside the cave as evidenced by the presence of some lithic tools, especially hammerstones.

In any event, that which occurred in the Cueva Des-Cubierta is clearly different to what went on at other Neanderthal occupations, where no such assemblage of crania without any post-cranial elements has ever been documented.

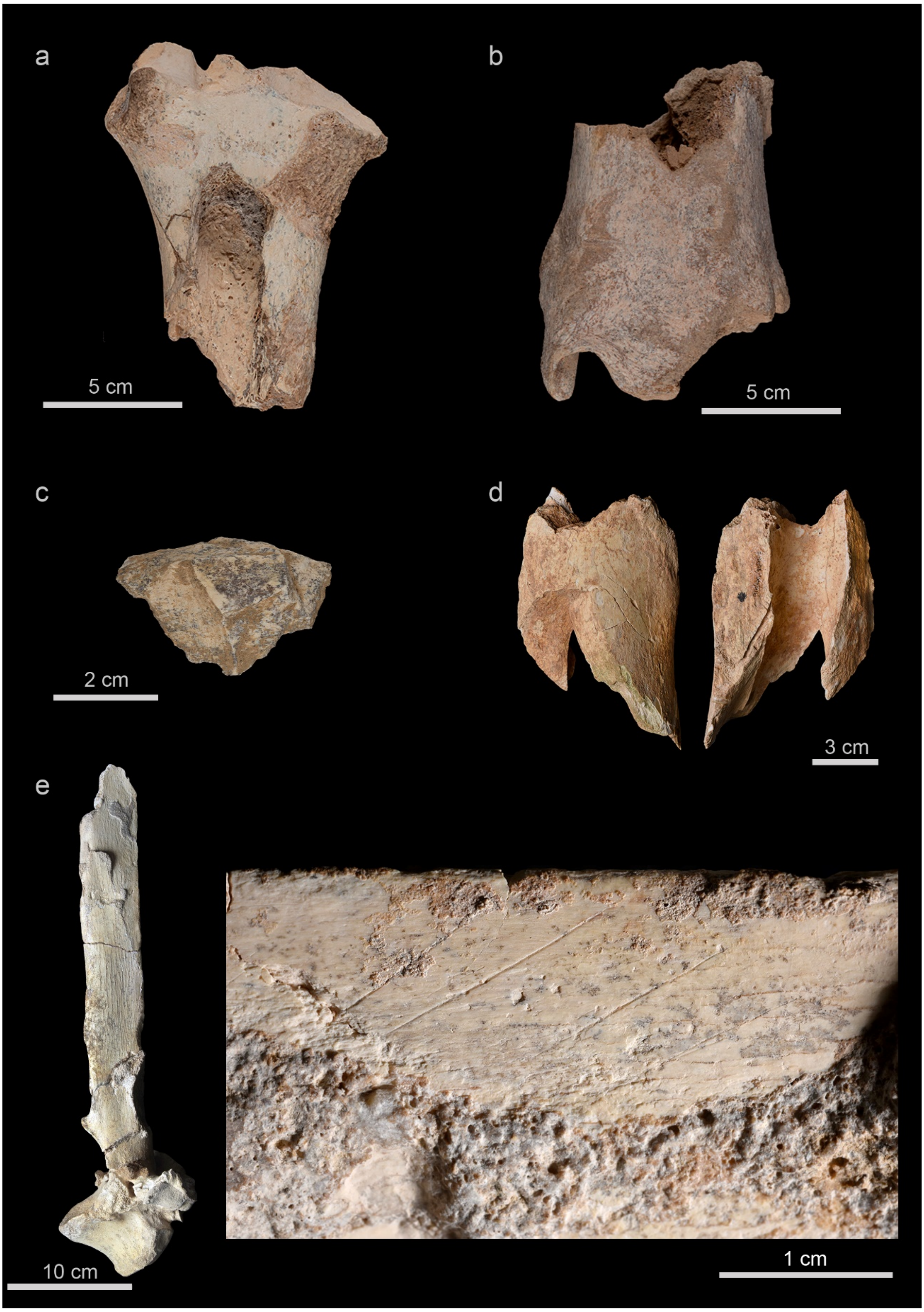

Supplementary Figure 40. Faunal remains from Level 3 of the Cueva Des-Cubierta cave with anthropogenic modifications. a) Proximal part of a bovine radius (11/13/CDC/I'40/1/16) with a green fracture pattern. b) Distal part of a bovine tibia (14/11/CDC/J'38/101/230) with green fracture planes. c) Bone flake (16/12/CDC/J'37/101/78) from a long bone of a large animal. d) Humerus shaft (14/11/CDC/I'42/101/12) of a bovine with a green fracture pattern. e) Cut marks on the spinous process of

a bovine thoracic vertebra (15/13/CDC/H'43/101/44). Photo credit: Alfonso Dávila (a, b, c and d), Mario Torquemada (e).

These crania were taken into the cave after most of the food provided by the captured animals was consumed elsewhere. As suggested in the main text, these crania may have been kept as hunting trophies and thus had a symbolic use (e.g., to project power, authority or hunting prowess, etc.). The cave is thus a new type of archaeological site.

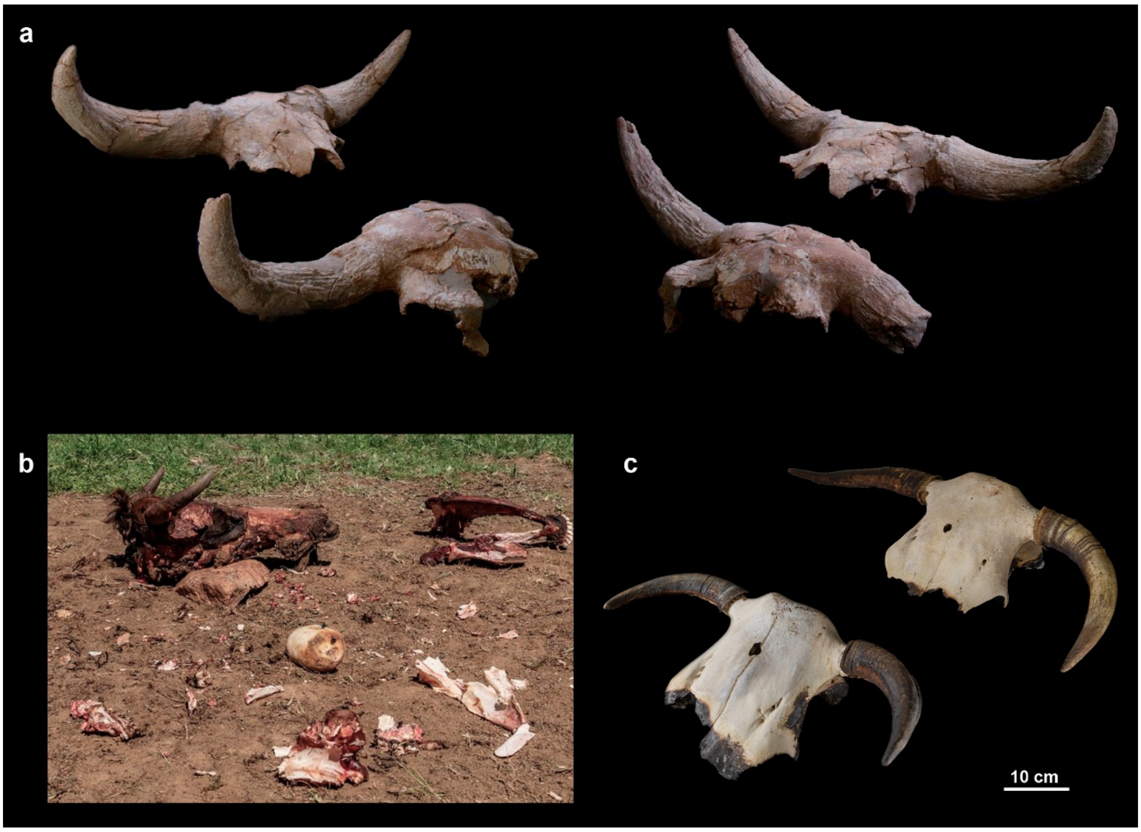

Supplementary Figure 41. Large bovine crania showing anthropogenic bone breakage. a) Two bison crania (11/13/CDC/G'42/1/14 and 11/13/CDC/H'42/100/18-12/9/CDC/G'42/100/21) recovered in Level 3 of Cueva Des-Cubierta cave. b) General view of different cranial bones belonging to a specimen of *Bos taurus,* and of stone tools used during the experimental butchering. c) Appearance of the *Bos taurus* crania after experimental bone breakage. Photo credit: Javier Trueba/MSF (a), Raquel Asiaín (b), Alfonso Dávila (c).

Radiocarbon dating

Several attempts were made to date the bone remains from Level 3, but none yielded enough collagen to allow this. Even when using one of the large bovine petrous bones, in which organic matter is usually better preserved[254,255], no dating could be made.

A minimum dating of *c*. 43 ka cal BP was obtained for a charcoal fragment from square H'45 in Level 2. Its maximum age, however, might lie beyond the range of the method used. Details of the provenience of this sample are provided in Supplementary Table 27. The result of this dating, and its calibration, are shown in Supplementary Table 28. Supplementary Figure 42 graphically presents the results of this calibration.

The age of the above charcoal sample sets a minimum age for Level 3 (which stratigraphically lies below Level 2) at >43 ka.

Supplementary Table 27. ^{14}C dating and sample provenience.

Collection	Sample	Square	Level	x/y/z	Description	Laboratory
16/09/2014	14/11/CDC/H'45/N5.5/123OX	H'45	2	432076.95/4530746.17/1109.77	Charcoal	ORAU

(x,y) are UTM coordinates, ED50 datum; (z) refers to heights above sea level (in m).

Supplementary Table 28. ^{14}C dating, results.

Sample	Lab-ID	$\delta^{13}C$ (‰)	Date BP (anni)	Age cal BP (68.3%)	Age cal BP (95.4%)
14/11/CDC/H'45/N5.5/123OX	OxA-31346	-24.50	44000±3600	51346-44319	Out of range-53154 (10.9%), 52964-43402 (84.6%)

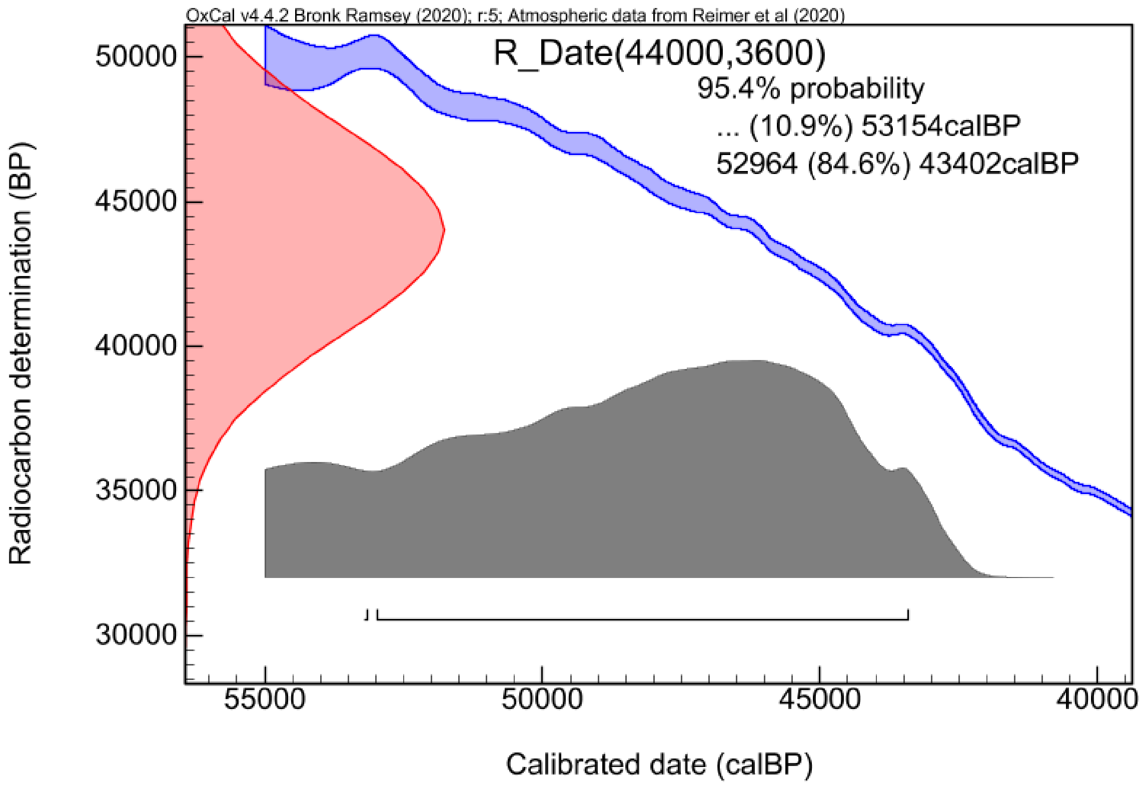

Supplementary Figure 42. Calibration of the AMS ^{14}C dating for sample OxA-31346 (Level 2). The red area indicates the Gaussian probability distribution for the determined (uncorrected) radiocarbon date. The blue strip indicates the IntCal20 calibration curve. The grey area represents the probability density function of the calibrated age.

U/Th dating

Supplementary Table 29 shows the locations at which the S1 and S2 speleothem samples were taken. S2, a stalagmite showing centimetric vertical development, was sampled in square K'41 (Supplementary Figure 43), where it formed over speleothem S1. The sample that was dated (by the CENIEH Uranium Series Laboratory) comes from the upper part of this stalagmite (Supplementary Figure 44). Another sampling point was established at the base of the sample to verify that the speleothem on which the stalagmite grew corresponded to S1.

Speleothem S1, a flowstone about 30 cm thick, was also sampled in square I’35 (Supplementary Figure 45). The initial sample, from the upper 5 cm of the flowstone, was divided into two. One subsample (CDC12_I’35_1_6) was sent to the Geochronology Laboratory of the Jaume Almera Institute of Earth Sciences (ICTJA-CSIC), and the other to the CENIEH Uranium Series Laboratory. This laboratory established five sampling points over the 5 cm of thickness of the speleothem (Supplementary Table 29 and Figure 46), and obtained a date for each.

Supplementary Table 29. U-series dating. Sample provenience. Samples CDC12_I’35_1_6 and SU-15-82 have the same provenience but were analysed by different laboratories. (the ICTJA-CSIC and CENIEH).

Collection	Lad ID	Square	x/y/z	Description	Laboratory
05/02/2020	SU-20032-1-02	K’41	432080.32/4530742.65/1108.24	S2 stalagmite (top of the stalagmite)	CENIEH
05/02/2020	SU-20032-1-01	K’41	432080.32/4530742.65/1108.24	S1 flowstone under S2 stalagmite	CENIEH
19/09/2012	CDC12_I’35_1_6	I’35	432079.34/4530736.69/1109.35	S1 flowstone (bulk sample)	ICTJA
19/09/2012	SU-15-82a	I’35	432079.34/4530736.69/1109.35	S1 flowstone (top)	CENIEH
19/09/2012	SU-16-82b	I’35	432079.34/4530736.69/1109.35	S1 flowstone (upper part)	CENIEH
19/09/2012	SU-16-82c	I’35	432079.34/4530736.69/1109.35	S1 flowstone (middle)	CENIEH
19/09/2012	SU-16-82d	I’35	432079.34/4530736.69/1109.35	S1 flowstone (lower part)	CENIEH
19/09/2012	SU-15-82e	I’35	432079.34/4530736.69/1109.35	S1 flowstone (bottom)	CENIEH

(x,y) are UTM coordinates, datum ED50; (z) refers to height above sea level (in m).

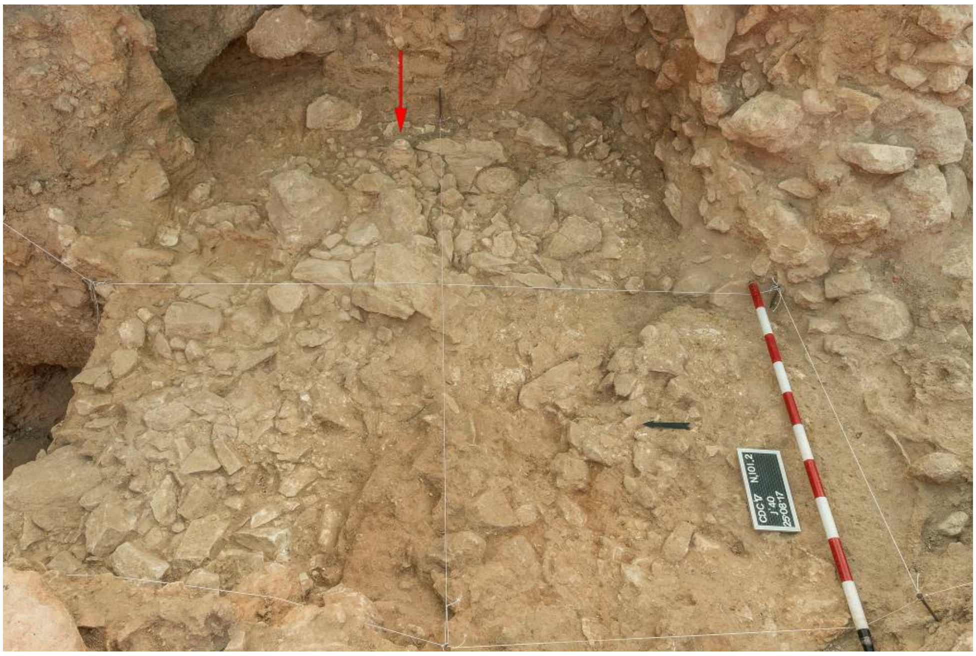

Supplementary Figure 43. Provenience of the dated S2 sample. Photo credit: Alfonso Dávila.

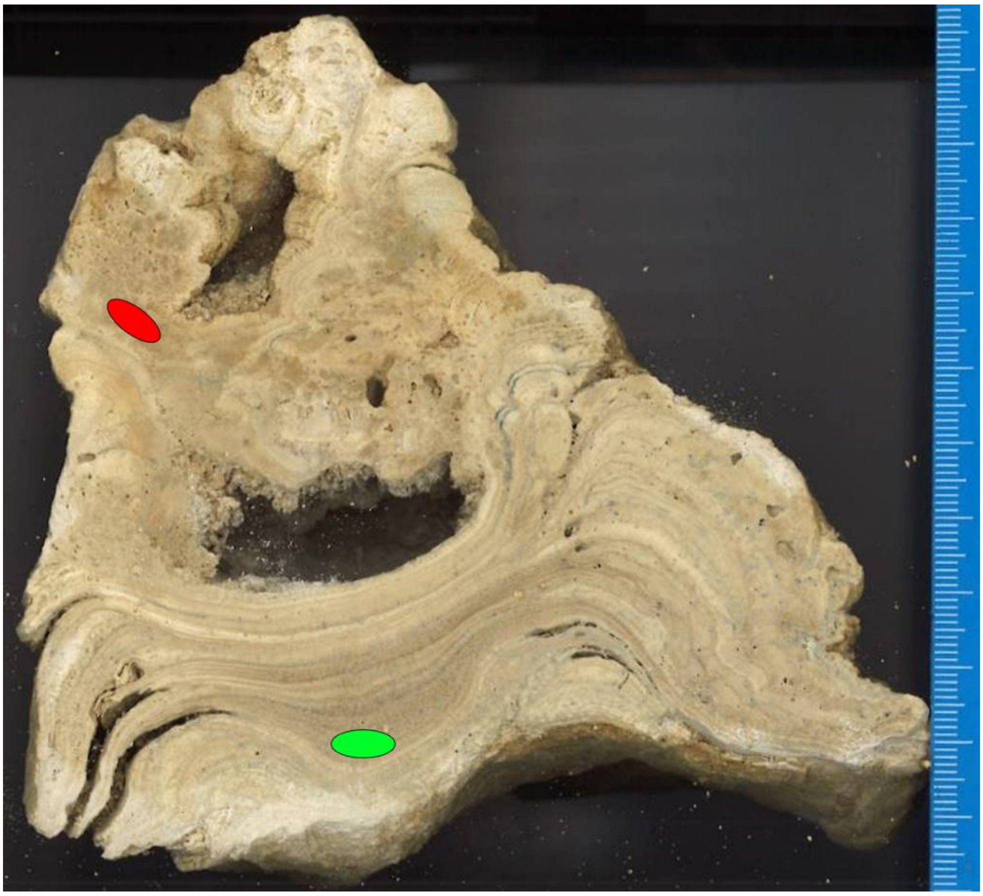

Supplementary Figure 44. Section of the S2 stalagmite over the S1 speleothem, with the areas sampled for dating indicated. The red ellipse indicates the S2 sampling point; the green ellipse indicates the S1sampling point. Photo credit: Fernando Jiménez Barredo/CENIEH.

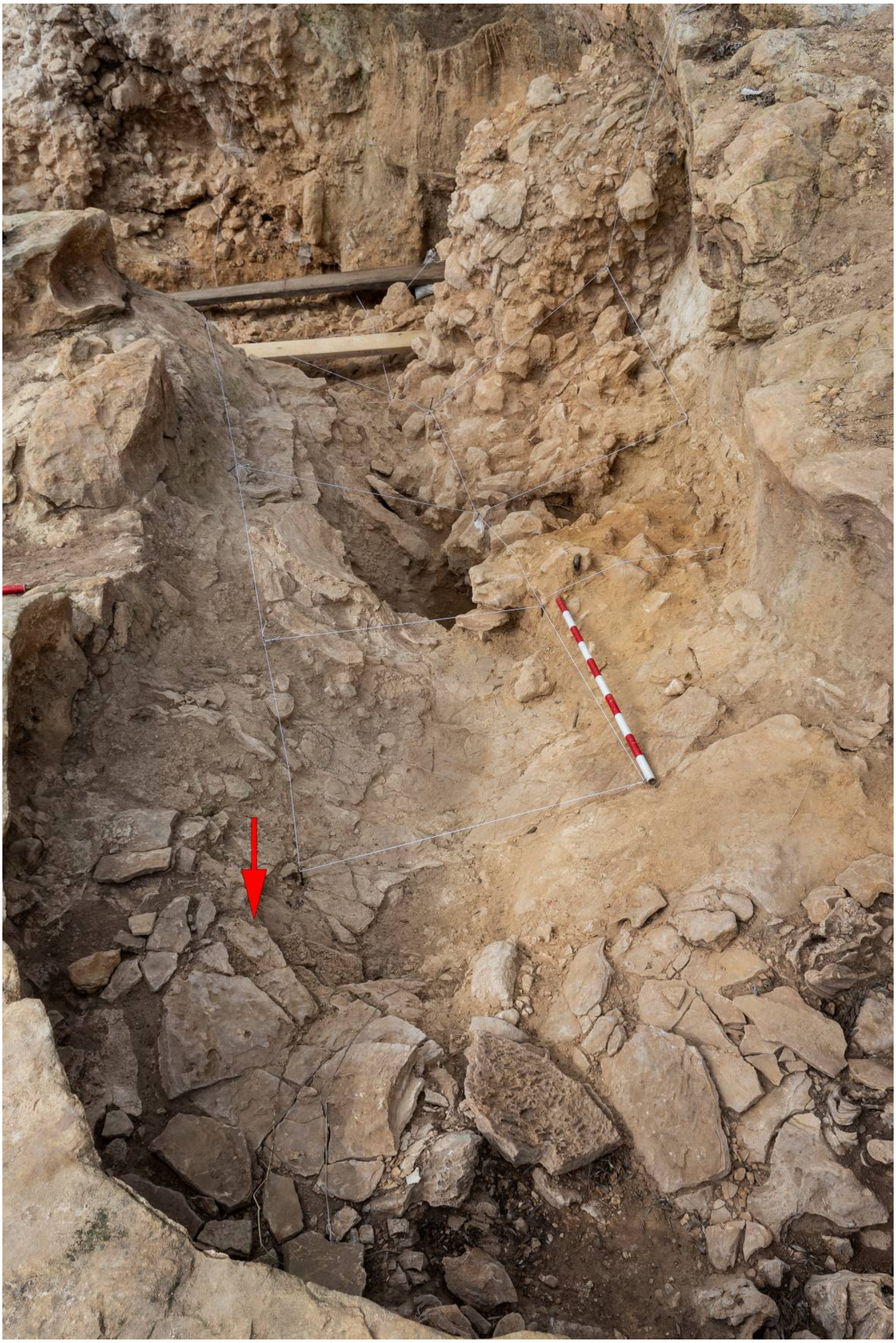

Supplementary Figure 45. Provenience of the dated S1 sample. Photo credit: Alfonso Dávila.

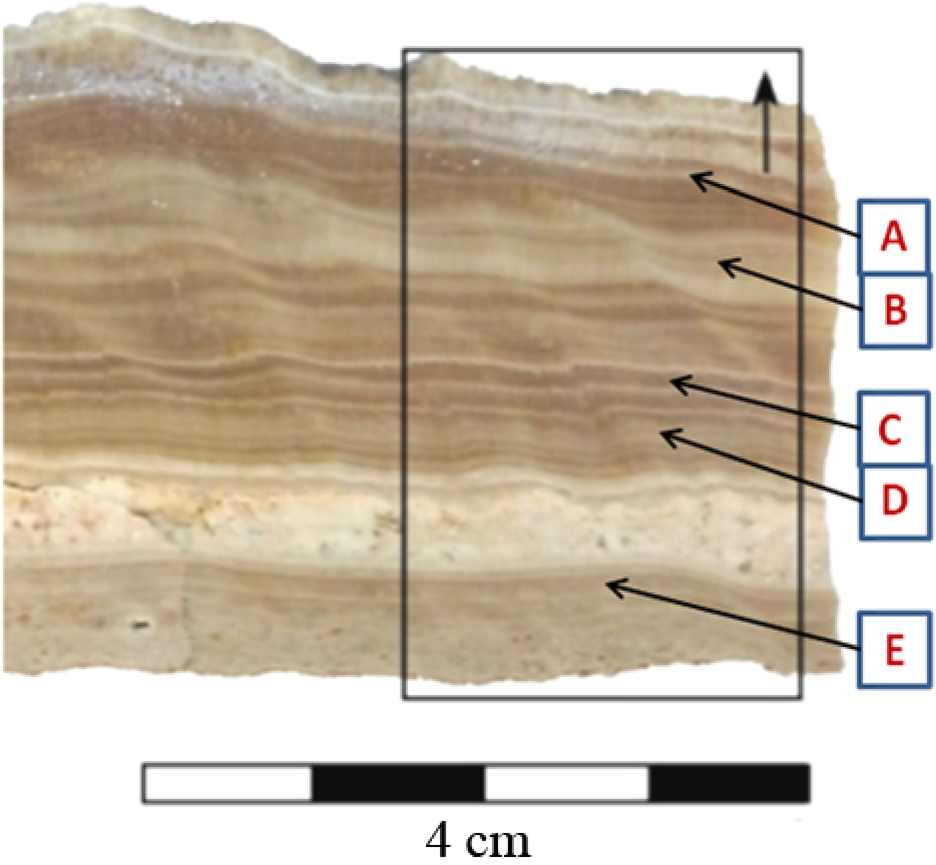

Supplementary Figure 46. Section of the S1 flowstone and detail of the microsampling performed at the CENIEH. The vertical arrow indicates the polarity of the flowstone; the oblique arrows indicate the microsampling points. Photo credit: Fernando Jiménez Barredo/CENIEH.

The S2 stalagmite was dated to around 135 ka (Supplementary Table 30); it therefore formed at the end of MIS6, still within the Middle Pleistocene (the boundary between the Middle and Late Pleistocene is at ~129 ka[256]). Consequently, the overlying Levels 4, 3 and 2 had to be deposited later, within the Late Pleistocene.

The dates obtained by the two laboratories for S1, which stratigraphically lies below S2, places it in MIS7 (spanning 243-191 ka[257]). According to the results returned by the CENIEH, the S1 sample from square I'35 gave dates from 231 ka (bottom) to 185 ka (top), while the sample from square K'41, where S1 is located immediately below stalagmite S2, was dated to 223 ka (Supplementary Tables 30 and 31). The latter result agrees with the stratigraphically lower position of the S1 flowstone with respect to the S2 stalagmite.

Supplementary Table 30. U series dating. $^{234}U/^{230}Th$ speleothem datings (CENIEH).

Stratigraphy	Sample	Lab ID	^{238}U	^{232}Th	$^{230}Th/^{232}Th$	$\delta^{234}U$	$^{230}Th/^{238}U$	^{230}Th age uncorrected	^{230}Th age corrected	$\delta^{234}U_{initial}$ estimated	^{230}Th age corrected
			ng x g^{-1}	ng x g^{-1}	Atomic ratio (x10^{-6})		Activity ratio				anni BP
S2 flowstone	SU-20032-1-02	SU 411	177.9 ± 0.5	51.6 ± 0.5	54.0 ± 0.04	191.6 ± 1.0	0.957 ± 0.001	164072 ± 7037	135782 ± 1988	304.4 ± 1.6	135713 ± 1988
S1 flowstone under stalagmite S2	SU-20032-1-01	SU 410	160.1 ± 0.6	32.5 ± 0.2	93.2 ± 0.05	204.3 ± 0.8	1160 ± 0.001	271926 ± 21771	223551 ± 2682	440.1 ± 1.7	223482 V2682
S1 flowstone (top)	SU-15-82a	ES6H	197.1 ± 0.4	3061 ± 0.062	887 ± 18	17 ± 7.8	0.8355 ± 0.0022	185854 ± 4725	185414 ± 4711	17 ± 13	185347 ± 4711
S1 flowstone (upper part)	SU-15-82b	SU77	143.9 ± 0.5	3025 ± 0.061	640 ± 15	7 ± 1	0.8369 ± 0.0042	192570 ± 3600	188918 ± 3532	7 ± 0.2	188851 ± 3532
S1 flowstone (middle)	SU-15-82c	SU75	226.0 ± 0.5	4148 ± 0.083	728 ± 16	-14 ± 3	0.8319 ± 0.0044	203909 ± 5400	200418 ± 5310	-14 ± 0.5	200351 ± 5310
S1 flowstone (lower part)	SU-15-82d	SU74	188.3 ± 0.6	7230 ± 0.145	365 ± 8	9 ± 2	0.8720 ± 0.0044	215597 ± 5150	207415 ± 6145	9 ± 0.4	207348 ± 6145
S1 flowstone (bottom)	SU-15-82e	SU 73	155.0 ± 0.5	7057 ± 0.141	331 ± 8	40 ± 8	0.9376 ± 0.0047	242089 ± 11740	231100 ± 7650	40 ± 1.4	231033 ± 7650

$\delta^{234}U = 1000\ [(^{234}U/^{238}U) - 1]$; $\delta^{234}U_o = \delta^{234}U_{meas} * e^{\lambda 234t}$ BP: Before Present refers to before AD 1950. Uncertainties (±) expressed in terms of 2σ.

Supplementary Table 31. U-series dating. Results of $^{234}U/^{230}Th$ speleothem datings (ICTJA).

Sample	Lab. Ref.	^{238}U (ppm)	^{232}Th (ppm)	$^{234}U/^{238}U$	$^{230}Th/^{232}Th$	$^{230}Th/^{234}U$	Nominal date (anni BP)
CDC12_I'35_1_6	4512	0.26	0.02	1.05 ± 0.04	39.821 ± 4.689	0.82 ± 0.04	184519 + 22329/ -18601

Supplementary References

143. Baquedano, E. et al. Creación y musealización del Parque Arqueológico del Calvero de la Higuera (Pinilla del Valle, Comunidad de Madrid), en el valle alto del Lozoya: El Valle de los Neandertales. *Espacio, Tiempo y Forma* (Serie I) **8**, 155-180 (2015).

144. Fernández-González, F. La vegetación del valle del Paular (Sierra de Guadarrama, Madrid), I. *Lazaroa* **12**, 153-272 (1991).

145. Prado, C. *Descripción física y geológica de la provincia de Madrid.* (Junta General de Estadística, 1864).

146. Alférez, F. et al. Descubrimiento del primer yacimiento cuaternario (Riss-Würm) de vertebrados con restos humanos en la provincia de Madrid (Pinilla del Valle). *Col. Paleontol.* **37**, 15-32 (1982).

147. Alférez, F. et al. Paleontología del Cuaternario. Yacimiento cuaternario de Pinilla del Valle. in *Madrid en sus orígenes*, 1-11 (Comunidad de Madrid. Consejería de Cultura, Deportes y Turismo, 1983).

148. Alférez, F., Molero, G. & Maldonado, E. Estudio preliminar del úrsido del yacimiento del cuaternario medio de Pinilla del Valle (Madrid). *Col. Paleontol.* **40**, 59-67 (1985).

149. Alférez, F. Dos molares humanos procedentes del yacimiento del Pleistoceno Medio de Pinilla del Valle (Madrid). *Trab. Antropol.* **19**, 303 (1985).

150. Alférez, F. & Iñigo, F. Los restos de *Dicerorhinus hemitoechus* (Perissodactyla, Mammalia) del Pleistoceno Medio de Pinilla del Valle (Madrid). *Actas Paleontol.*, 25-45 (1990).

151. Alférez, F. & Roldán, B. Un molar humano anteneandertal con patología traumática procedente del yacimiento cuaternario de Pinilla del Valle (Madrid). *Munibe (Antropol.-Arkeol.)* **supl. 8**, 183-188 (1992).

152. Alférez, F. & Buitrago, A. M. Los restos de *Sus scrofa* (Artiodactyla, Mammalia) del yacimiento Pleistoceno de Pinilla del Valle (Madrid, España). *Estudios geol.* **79**, e092 (2019).

153. Buitrago, A. M. Estudio de los artiodáctilos del yacimiento del Pleistoceno Medio de Pinilla del Valle (Madrid). (Universidad Complutense, 1992).

154. Maldonado, E. El yacimiento cuaternario de Pinilla del Valle (Madrid). *Cuad. INICE* **38**, 9-22 (1991).

155. Toni, I. & Molero, G. Los roedores (Rodentia, Mammalia) del yacimiento cuaternario de Pinilla del Valle (Madrid). *Actas Paleontol*. 359-373 (1990).

156. Baquedano, E. et al. Selection of cave shelter by neanderthals (H*omo neanderthalensis*) and spotted hyaenas (*Crocuta crocuta*) at the Calvero de la Higuera (Pinilla del Valle, Madrid Region, Spain). *ARPI* **4** extra, 5-19 (2016).

157. Laplana, C. et al. Un assemblage de petits vertébrés hautement diversifié de la fin du MIS5 dans un environnement montagnard au centre de l'Espagne (Cueva del Camino, Pinilla del Valle, Communauté Autonome de Madrid). *Quaternaire* **24**, 207-216 (2013).

158. Blain, H.-A. et al. MIS5/4 transition in a mountain environment: Herpetofaunal assemblages from Cueva del Camino, central Spain. *Boreas* **43**, 107–120 (2014).

159. Díez, J. C. Estudio tafonómico de los macrovertebrados de yacimientos del Pleistoceno Medio. *Complutum* **4**, 21-40 (1993).

160. Moclán, A. et al. Cut marks made with quartz tools: An experimental framework for understanding cut mark morphology, and its use at the Middle Palaeolithic site of the Navalmaíllo Rock Shelter (Pinilla del Valle, Madrid, Spain). *Quat. Int.* **493**, 1-18 (2018).

161. Moclán, A. et al. Identifying the bone-breaker at the Navalmaíllo Rock Shelter (Pinilla del Valle, Madrid) using machine learning algorithms. *Archaeol. Anthropol. Sci.* **12**, 46 (2020).

162. Márquez, B. et al. Evidence of a neanderthal-made quartz-based technology at Navalmaíllo rockshelter (Pinilla del Valle, Madrid Region, Spain). *J. Anthropol. Res.* **69**, 373-395 (2013).

163. Márquez, B., Baquedano, E., Pérez-González, A. & Arsuaga, J. L. Microwear analysis of Mousterian quartz tools from the Navalmaíllo Rock Shelter (Pinilla del Valle, Madrid, Spain). *Quat. Int.* **424**, 84-97 (2016).

164. Márquez, B., Baquedano, E., Pérez-González, A. & Arsuaga, J. L. Denticulados y muescas: ¿para qué sirven? Estudio funcional de una muestra musteriense en cuarzo del Abrigo de Navalmaíllo (Pinilla del Valle, Madrid, España). *Trab. Prehist.* **74**, 26-46 (2017).

165. Pérez-González, A. et al. Aproximación geomorfológica a los yacimientos del Pleistoceno Superior del Calvero de la Higuera en el Valle Alto del Lozoya (Sistema Central Español, Madrid). *Zona Arqueol.* **13**, 403-420 (2010).

166. Laplana, C. et al. Cold-climate rodent indicators for the Late Pleistocene of Central Iberia: New data from the Buena Pinta Cave (Pinilla del Valle, Madrid Region, Spain). *C. R. Palevol* **15**, 696-706 (2016).

167. Laplana, C. et al. How far into Europe Did Pikas (Lagomorpha: Ochotonidae) Go during the Pleistocene? New Evidence from Central Iberia. *PLoS ONE* **10**, e0140513 (2015).

168. Ruiz Zapata, B. et al. Evolución de la vegetación durante el Pleistoceno Superior y el Holoceno en el valle alto del río Lozoya. Yacimiento arqueopaleontológico de la cueva de la Buena Pinta (Pinilla del Valle, Sistema Central Español). *Geogaceta* **44**, 83-86 (2008).

169. Laplana, C. et al. Un caso de asociaciones de microvertebrados pleistocenas mezcladas por reelaboración en ambientes cársticos: La Cueva de la Buena Pinta (Pinilla del Valle, Comunidad de Madrid). In Reolid, M. (ed.), Libro de Resúmenes, XXXI Jornadas de Paleontología. Sociedad Española de Paleontología, 152-153 (2015).

170. Brunet-Lecomte, P. & Paunesco, A.-C. Morphométrie comparée de la première molaire inférieure du campagnol *Microtus (Terricola) vaufreyi tautavelensis* (Rodentia, Arvicolidae) du gisement Pléistocène moyen de l'Arago (Pyrénées, France) et inférences paléoclimatiques. *Quaternaire* **15**, 263-268 (2004).

171. Laplana, C. & Sevilla, P. Documenting the biogeographic history of *Microtus cabrerae* through its fossil record. *Mamm. Rev.* **43**, 309-332 (2013).

172. Mihevc, A., Slabe, T. & Sebela, S. Denuded caves - an inherited element in the karst morphology: the case from Kras. *Acta Carsologica* **27**, 165-174 (1998).

173. De Vicente, G., Cloetingh, S., Van Wees, J. D. & Cunha, P. P. Tectonic classification of Cenozoic Iberian foreland basins. *Tectonophysics* **502**, 38-61 (2011).

174. Salazar, A. El modelado: sin valles no hay montañas. *Enseñ. Cienc. Tierra* **26**, 68-76 (2018).

175. Bellido, F. et al. *Mapa Geológico de España a E. 1:50.000, Buitrago de Lozoya (484)* (IGME, 1991).

176. Barea, J. Geomorfología y evolución paleoclimática durante el Cuaternario a partir del estudio de los macizos kársticos de los bordes del Sistema Central y de Valporquero, León. (Universidad Complutense, 2001).

177. Fernández Navarro, L. Monografía geológica del valle del Lozoya. *Trab. Mus. Nac. Cien. Nat. Serie Geológica* **12**, 1-100 (1915).

178. Carbó, A., Alférez, F., Hoster, M., Carrasco, R. & Domínguez, A. Prospección geofísica de cavidades naturales en el yacimiento del Pleistoceno Medio de Pinilla del Valle (Madrid). *Geociencias* **5**, 1-8 (1990).

179. Camacho, A. G., Vieira, R., Montesinos, F. G. & Cuéllar, V. A gravimetric 3D Global inversion for cavity detection. *Geophys. Prospect.* **42**, 113-130 (1994).

180. Karampaglidis, T. La evolución geomorfológica de la cuenca de drenaje del río Lozoya (Comunidad de Madrid, España) (Universidad Complutense, 2015).

181. Tesakov, A. S. et al. Early-Middle Pleistocene environmental and biotic transition in northwestern Armenia, southern Caucasus. *Palaeont. Electron.* **22.2.25A**, 1-39 (2019).

182. Maul, L. C. & Parfitt, S. A. Micromammals from the 1995 Mammouth Excavation at West Runton, Norfolk, UK: Morphometric data, biostratigraphy and taxonomic reappraisal. *Quat. Int.* **228**, 91-115 (2010).

183. Schwartz, J. H. & Tattersall, I. The human chin revisited: what is it and who has it? *J. Hum. Evol.* **38**, 367-409 (2000).

184. Coqueugniot, H. & Minugh-Purvis, N. in *Patterns of Growth and Development in the Genus* Homo (ed. Thompson, J. T., Krovitz, G. E. & Nelson, A. H.) (Cambridge University Press, 2003).

185. Quam, R. et al. The Neandertals of northeastern Iberia: New remains from the Cova del Gegant (Sitges, Barcelona). *J. Hum. Evol.* **81**, 13-28 (2015).

186. Daura, J. et al. A neandertal mandible from the Cova del Gegant (Sitges, Barcelona, Spain). *J. Hum. Evol.* **49**, 56-70 (2005).

187. Walker, M. J., Lombardi, A. V., Zapata, J. & Trinkaus, E. Neandertal mandibles from the Sima de las Palomas del Cabezo Gordo, Murcia, southeastern Spain. *Am. J. Phys. Anthropol.* **142**, 261-272 (2010).

188. Mallegni, F. & Trinkaus, E. A reconsideration of the Archi 1 Neandertal mandible. *J. Hum. Evol.* **33**, 651-668 (1997).

189. Madre-Dupouy, M. *L´Enfant du Roc de Marsal: Étude Analytique et Comparative.* (Editions du CNRS, Paris, 1992).

190. Dean, M., Stringer, C. B. & Bromage, T. G. Age at death of the Neanderthal child from Devil's Tower, Gibraltar and the implications for studies of general growth and development in Neanderthals. *Am. J. Phys. Anthropol.* **70**, 301-309 (1986).

191. Mallegni, F. & Ronchitelli, A. T. Deciduous teeth of the Neandertal mandible from Molare Shelter, near Scario (Salerno, Italy). *Am. J. Phys. Anthropol.* **79**, 475-482 (1989).

192. Arsuaga, J. L. et al. The human remains from Cova Negra (Valencia, Spain) and their place in European Pleistocene human evolution. *J. Hum. Evol.* **18**, 55-92 (1989).

193. Garralda, M.-D. & Vandermeersch, B. Les Néandertaliens de la grotte de Combe-Grenal (Domme, Dordogne, France)/The Neanderthals from Combe-Grenal cave (Domme, Dordogne, France). *Paléo* **12**, 213-259 (2000).

194. Billy, G. L'enfant magdalénien de la grotte du Figuier (Ardèche). *L'Anthropologie* **83**, 223-252 (1979).

195. Heim, J.-L. L'enfant magdalénien de La Madeleine. *L'Anthropologie* **95**, 611-638 (1991).

196. Zilhão, J. & Trinkaus, E. *Portrait of the Artist as a Child. The Gravettian Human Skeleton from the Abrigo do Lagar Velho and its Archeological Context* (Instituto Português de Arqueologia, 2002).

197. Bayle, P., Braga, J., Mazurier, A. & Macchiarelli, R. Brief communication: High-resolution assessment of the dental developmental pattern and characterization of tooth tissue proportions in the late Upper Paleolithic child from La Madeleine, France. *Am. J. Phys. Anthropol.* **138**, 493-498 (2009).

198. Turner, C. G. I., Nichol, C. R. & Scott, G. R. in *Advances in Dental Anthropology* (ed. Kelley, M. A. & Larsen, C. S.) 13-31 (Wiley-Liss, 1991).

199. Legoux, P. in *L`enfant du Pech de l`Azé* (ed. Ferembach, D., Legoux, P., Fenart, R., Empereur-Buisson, R. & Vlcek, E.) 53-87 (Archives de l’Institut de Paléontologie Humaine, 1970).

200. Tillier, A. M. *Les Enfants Mousteriens de Qafzeh* (CNRS editions, 1999).

201. Bailey, S. & Hublin, J. J. in *Neanderthals Revisted: New Approaches and Perspective* (ed. Harvati, K. & Harrington, M. A.) 191-210 (Springer, Dordrecht, The Netherlands, 2006).

202. Gómez-Robles, A. et al. Geometric morphometric analysis of the crown morphology of the lower first premolar of hominins, with special attention to Pleistocene *Homo*. *J. Hum. Evol.* **55**, 627-638 (2008).

203. Bermúdez de Castro, J. M. et al. Early Pleistocene hominin deciduous teeth from the *Homo antecessor* Gran Dolina-TD6 bearing level (Sierra de Atapuerca, Spain). *Am. J. Phys. Anthropol.* **163**, 602-615 (2017).

204. Paunesco, A. C. Les rongeurs de la Caune de l'Arago (Tautavel, Pyrénées occidentales): paléontologie, biostratigraphie, paléoécologie. *Trav. Inst. Spéol. Emile Racovitza* **36**, 135-206 (1997).

205. Baquedano, E., Arsuaga, J. L. & Pérez-González, A. in *Actas de las quintas jornadas de Patrimonio Arqueológico en la Comunidad de Madrid* (ed. Santonja, M.). (Dirección General de Patrimonio Histórico, 2011).

206. Pandolfi, L. et al. Late Pleistocene last occurrences of the narrow-nosed rhinoceros *Stephanorhinus hemitoechus* (Mammalia, Perissodactyla) in Italy. *Riv. Ital. Paleontol. S.* **123**, 177-192 (2017).

207. Higham, T. et al. The timing and spatiotemporal patterning of Neanderthal disappearance. *Nature* **7514**, 306-309 (2014).

208. Dietrich, W. O. Fossile Löwen im Europäischen und afrikanischen Pleistozän. *Paläontol. Abh,. Abt. A* **3**, 333-366 (1968).

209. Argant, A. & Brugal, J.-Ph. The cave lion *Panthera* (*Leo*) *spelaea* and its evolution: *Panthera spelaea intermedia* nov. subspecies. *Acta zool. cracov.* **60**, 59-104 (2017).

210. Schütt, G. Untersuchungen am Gebiß von *Panthera leo fossilis* (V. Reichenau, 1906) und *Panthera leo spelaea* (Goldfuss, 1810). Ein Beitrag zum Systematik der pleistozänen Großkatzen Europas. *Neues Jahrb. Geol. Palaontol. Abh.* **134**, 192-220 (1969).

211. Hemmer, H. Untersuchungen zur Stammesge-schichte der Pantherkatzen (Pantherinae). Teil III. Zur Artgeschichte des Löwen, *Panthera (Panthera) leo* (Linnaeus 1758). *Veröff. Zool. Staatssamml.* **17**, 167-280 (1974).

212. Schütt, G. & Hemmer, H. Zur Evolution des Löwen (*Panthera leo* L.) im europäischen Pleistozän. *Neues Jahrb. Geol. Palaontol. Mon.* **4**, 228-255 (1978).

213. Marciszak, A., Schouwenburg, C. & Darga, R. Decreasing size process in the cave (Pleistocene) lion *Panthera spelaea* (Goldfuss, 1810) evolution and review. *Quat. Int.* **339-340**, 245-257 (2014).

214. Bahain, J. J., Qingfeng, S., Falguères, C. & Garcia, T. Datation ESR/U-TH du site paléontologique de Romain-La-Roche (Doubs, France). *Quaternaire* **22**, 201-209 (2011).

215. Kahlke R.-D., Maul, L. C., Meyrick, R. A., Stebich, M., & Grasselt T. in *The Quaternary of Central Germany. Field Guide* (ed. Meyrick, R. A. & D. C. Schreve, D. C.) (Quaternary Research Association London, 2002).

216. Sotnikova, M. & Nikolskiy, P. Systematic position of the cave lion *Panthera spelaea* (Goldfuss) based on cranial and dental characters. *Quat. Int.* **142–143**, 218–228 (2006).

217. Dawkins, W. B. & Sandford, W. A. *The British Pleistocene Mammalia. Parts I-II. British Pleistocene Felidae.* Felis spelaea, *Goldfuss* (Palaeontographical Society Monographs, 1866).

218. Argant, A. Carnivores (Canidae, Felidae et Ursidae) of Romain-la-Roche (Doubs, France). *Rev. Paleobiol.* **29**, 495-601 (2010).

219. Argant, A. Etude de l'exemplaire de *Panthera spelaea* (Goldfuss, 1810) (Mammalia, Carnivora, Felidae) du gisement Pléistocène moyen recent de la grotte d'Azé (Soane et Loire). *Rev. Paleobiol*. **7**, 449-466 (1988).

220. Álvarez-Lao, D. & Méndez, M. Latitudinal gradients and indicator species in ungulate paleoassemblages during the MIS3 in W Europe. *Palaeogeogr., Palaeoclimatol., Palaeoecol.* **449**, 455-462 (2016).

221. Vereshchagin, N. K. & Baryshnikov, G. F. in *Paleoecology of the mammoth fauna in the Eurasian Arctic* (ed. Hopkins, D. M. et al.)(Academic Press, 1982).

222. Guthrie, R. D. *Frozen fauna of the Mammoth Steppe: The story of Blue Babe.* (University of Chicago, 1990).

223. Mazza, P. Ethological inferences on Pleistocene rhinoceroses of Europe. *Atti Acc. Naz. Lin.* **9**, 127-137 (1993).

224. Perea, R., Girardello, M. & San Miguel, A. Big game or big loss? High deer densities are threatening woody plant diversity and vegetation dynamics. *Biodivers. Conserv.* **23**, 1303-1318 (2014).

225. Blain, H.-A. et al. The Middle to Late Pleistocene herpetofaunal assemblages from the Jarama and Manzanares valleys (Madrid, central Spain): An ecological synthesis. *Quat. Int.* **520**, 49-63 (2019).

226. Abrunhosa, A. et al. Neanderthal raw material procurement strategies spatial analysis of lithic resources available in the Lozoya river valley (Madrid-Spain). in PESHE 6: 3. 7th Annual Meeting of the European Society for the Study of Human Evolution. Leiden (2017).

227. Domínguez-Rodrigo, M., Bunn, H. T. & Yravedra, J. A critical re-evaluation of bone surface modification models for inferring fossil hominin and carnivore interactions through a multivariate approach: Application to the FLK Zinj archaeofaunal assemblage (Olduvai Gorge, Tanzania). *Quat. Int.* **322–323**, 32–43 (2014).

228. Arriaza, M. C., Domínguez-Rodrigo, M., Yravedra, J. & Baquedano, E. Lions as Bone Accumulators? Paleontological and Ecological Implications of a Modern Bone Assemblage from Olduvai Gorge. *PLoS ONE* **11**, (2016).

229. De Ruiter, D. J. & Berger, L. R. Leopards as Taphonomic Agents in Dolomitic Caves—Implications for Bone Accumulations in the Hominid-bearing Deposits of South Africa. *J. Archaeol. Sci.* **27**, 665–684 (2000).

230. Domínguez-Rodrigo, M. & Pickering, T. R. A multivariate approach for discriminating bone accumulations created by spotted Hyenas and Leopards: harnessing actualistic data from east and Southern Africa. *J. Taphon.* **8**, 155–179 (2010).

231. Egeland, A., Egeland, C. P. & Bunn, H. T. Taphonomic Analysis of a Modern Spotted Hyena (Crocuta crocuta) Den from Nairobi, Kenya. *J. Taphon.* **6**, 275–299 (2008).

232. Kuhn, B. F., Berger, L. R. & Skinner, J. D. Examining criteria for identifying and differentiating fossil faunal assemblages accumulated by hyenas and hominins using extant hyenid accumulations. *Int. J. Osteoarchaeol.* **20**, 15–35 (2010).

233. Lacruz, R. & Maude, G. Bone Accumulations at Brown Hyena (*Parahyaena brunnea*) Den Sites in the Makgadikgadi Pans, Northern Botswana: Taphonomic, Behavioral and Palaeoecological Implications. *J. Taphon.* **3**, 43–53 (2005).

234. Pokines, J. T. & Kerbis Peterhans, J. C. Spotted hyena (*Crocuta crocuta*) den use and taphonomy in the Masai Mara National Reserve, Kenya. *J. Archaeol. Sci.* **34**, 1914–1931 (2007).

235. Prendergast, M. E. & Domínguez-Rodrigo, M. Taphonomic Analyses of a Hyena Den and a Natural-Death Assemblage Near Lake Eyasi (Tanzania). *J. Taphon,* **6**, 301–335 (2008).

236. Agenbroad, L. Excavation at the Hot Springs Mammoth Site: A Late Pleistocene Animal Trap. *Trans. Nebr. Acad. Sci. Affil. Soc.* **6**, 127–130 (1978).

237. Huguet, R. et al. Le gisement de Galería (Sierra de Atapuerca, Burgos, Espagne): un modèle archéozoologique de gestion du territoire durant le Pleistocene/ The site of Galeria (Sierra de Atapuerca, Burgos, Spain): a archaeozoological pattern for territory gestion during Pleistocene. *L'Anthropologie*, **105**, 237-257 (2001).

238. Martín-Perea, D. M. et al. Taphonomic analysis of Batallones-10, a Late Miocene drought-induced mammalian assemblage (Madrid basin, Spain) within the Cerro de los Batallones complex. *Palaeogeogr. Palaeoclimatol. Palaeoecol.* **578**, 110576 (2021).

239. Domínguez-Rodrigo, M. et al. Fluvial spatial taphonomy: a new method for the study of post-depositional processes. *Archaeol. Anthropol. Sci.* **10**, 1769–1789 (2018).

240. Organista, E. et al. Biotic and abiotic processes affecting the formation of BK Level 4c (Bed II, Olduvai Gorge) and their bearing on hominin behavior at the site. *Palaeogeogr. Palaeoclimatol. Palaeoecol.* **488**, 59–75 (2017).

241. Voorhies, M. R. Taphonomy and population dynamics of an early Pliocene vertebrate fauna. *University of Wyoming Contributions to Geology Special Paper* **1**, 1–69 (1969).

242. Backwell, L. R., Parkinson, A. H., Roberts, E. M., d'Errico, F. & Huchet, J.-B. Criteria for identifying bone modification by termites in the fossil record. *Palaeogeogr. Palaeoclimatol. Palaeoecol.* **337–338**, 72–87 (2012).

243. Behrensmeyer, A. K. Taphonomic and ecologic information from bone weathering. *Paleobiology* **4**, 150–162 (1978).

244. Cáceres, I., Esteban-Nadal, M., Bennàsar, M. & Fernández-Jalvo, Y. Was it the deer or the fox? *J. Archaeol. Sci.* **38**, 2767–2774 (2011).

245. Cáceres, I. et al. Osteophagia and dental wear in herbivores: actualistic data and archaeological evidence. *J. Archaeol. Sci.* **40**, 3105–3116 (2013).

246. Fernández-Jalvo, Y. & Andrews, P. *Atlas of Taphonomic Identifications: 1001+ Images of Fossil and Recent Mammal Bone Modification*. (Springer, 2016).

247. Hill, A. & Behrensmeyer, A. K. Natural Disarticulation and Bison Butchery. *Am. Antiq.* **50**, 141 (1985).

248. Jans, M. M. E., Nielsen-Marsh, C. M., Smith, C. I., Collins, M. J. & Kars, H. Characterisation of microbial attack on archaeological bone. *Journal of Archaeological Science* **31**, 87–95 (2004).

249. Mello Araujo, A. G. & Marcelino, J. C. The role of armadillos in the movement of archaeological material: an experimental approach. *Geoarchaeology – Int. J.* **18,** 433-460 (2003).

250. Lyman, R. L. Bone density and differential survivorship of fossil classes. *J. Anthropol. Archaeol.* **3**, 259–299 (1984).

251. Kreutzer, L. A. Bison and deer bone mineral densities: comparisons and implications for the interpretation of archaeological faunas. *J. Archaeol. Sci.* **19**, 271–294 (1992).

252. Faith, J. T. & Gordon, A. D. Skeletal element abundances in archaeofaunal assemblages: economic utility, sample size, and assessment of carcass transport strategies. *J. Archaeol. Sci.* **34**, 872–882 (2007).

253. Faith, J. T., Domínguez-Rodrigo, M. & Gordon, A. D. Long-distance carcass transport at Olduvai Gorge? A quantitative examination of Bed I skeletal element abundances. *J. Hum. Evol.* **56**, 247–256 (2009).

254. Lam, Y. M., Chen, X. & Pearson, O. M. Intertaxonomic variability in patterns of bone density and the differential representation of bovid, cervid, and equid elements in the archaeological record. *American Antiquity* **64**, 343-362 (1999).

255. Gamba, C. et al. Genome flux and stasis in a five millennium transect of European prehistory. *Nat. Commun.* **5**, 1-9 (2014).

256. Head, M. J., Pillans, B., Zalasiewicz, J. A., & ICS Subcommission of Quaternary Stratigraphy. Formal ratification of subseries for the Pleistocene Series of the Quaternary System. *Episodes* **44**, 241-247 (2021).

257. Lisiecki, L. E. & Raymo, M. E. A Pliocene-Pleistocene stack of 57 globally distributed benthic δ18O records. *Paleoceanography* **20**, PA1003 (2005).
