# Supplementary material for: A symbolic Neanderthal accumulation of large herbivore crania
Source: Nat Hum Behav. 2023 Jan 26;7(3):342–52. doi: 10.1038/s41562-022-01503-7 (PMC10038806; doi:10.1038/s41562-022-01503-7)
Supplement: Supplementary file 2 — Reporting Summary [file 41562_2022_1503_MOESM2_ESM.pdf]

nature portfolio

Corresponding author(s): Enrique Baquedano

Last updated by author(s): Oct 30, 2022

Reporting Summary

Nature Portfolio wishes to improve the reproducibility of the work that we publish. This form provides structure for consistency and transparency in reporting. For further information on Nature Portfolio policies, see our Editorial Policies and the Editorial Policy Checklist.

Statistics

For all statistical analyses, confirm that the following items are present in the figure legend, table legend, main text, or Methods section.

n/a	Confirmed	
☐	☒	The exact sample size (*n*) for each experimental group/condition, given as a discrete number and unit of measurement
☒	☐	A statement on whether measurements were taken from distinct samples or whether the same sample was measured repeatedly
☒	☐	The statistical test(s) used AND whether they are one- or two-sided<br>*Only common tests should be described solely by name; describe more complex techniques in the Methods section.*
☒	☐	A description of all covariates tested
☒	☐	A description of any assumptions or corrections, such as tests of normality and adjustment for multiple comparisons
☐	☒	A full description of the statistical parameters including central tendency (e.g. means) or other basic estimates (e.g. regression coefficient) AND variation (e.g. standard deviation) or associated estimates of uncertainty (e.g. confidence intervals)
☒	☐	For null hypothesis testing, the test statistic (e.g. *F*, *t*, *r*) with confidence intervals, effect sizes, degrees of freedom and *P* value noted<br>*Give P values as exact values whenever suitable.*
☒	☐	For Bayesian analysis, information on the choice of priors and Markov chain Monte Carlo settings
☒	☐	For hierarchical and complex designs, identification of the appropriate level for tests and full reporting of outcomes
☒	☐	Estimates of effect sizes (e.g. Cohen's *d*, Pearson's *r*), indicating how they were calculated

Our web collection on statistics for biologists contains articles on many of the points above.

Software and code

Policy information about availability of computer code

Data collection	The commercial software packages Excel 2013 and FileMaker Pro 7 were used to create the database with the items recovered from the site.
Data analysis	The spatial analysis was carried out using AutoCad 2013 and QGIS v. 3.14 software. The virtual reconstruction of the human remains was carried out using Mimics v. 18 software. The calculation of the abundance indices of the zooarchaeological collections was done using the Excel 2013 software. The statistical processing and graphical representation of the palynological data were carried out with the help of the Tilia v. 2.6.1 software. Calibration of the radiocarbon dates was performed using OxCal v. 4.4 software.

For manuscripts utilizing custom algorithms or software that are central to the research but not yet described in published literature, software must be made available to editors and reviewers. We strongly encourage code deposition in a community repository (e.g. GitHub). See the Nature Portfolio guidelines for submitting code & software for further information.

Data

Policy information about availability of data

All manuscripts must include a data availability statement. This statement should provide the following information, where applicable:

- Accession codes, unique identifiers, or web links for publicly available datasets
- A description of any restrictions on data availability
- For clinical datasets or third party data, please ensure that the statement adheres to our policy

The artefacts reported here are curated at the Museo Arqueológico y Paleontológico de la Comunidad de Madrid, Alcalá de Henares, Spain. The data that support the findings of this study are available from the corresponding author upon reasonable request.

Human research participants

Policy information about studies involving human research participants and Sex and Gender in Research.

Reporting on sex and gender	N/A
Population characteristics	N/A
Recruitment	N/A
Ethics oversight	N/A

Note that full information on the approval of the study protocol must also be provided in the manuscript.

Field-specific reporting

Please select the one below that is the best fit for your research. If you are not sure, read the appropriate sections before making your selection.

☐ Life sciences ☐ Behavioural & social sciences ☒ Ecological, evolutionary & environmental sciences

For a reference copy of the document with all sections, see nature.com/documents/nr-reporting-summary-flat.pdf

Ecological, evolutionary & environmental sciences study design

All studies must disclose on these points even when the disclosure is negative.

Study description	In this study, the faunal association of Level 3 of Cueva Des-Cubierta is analysed from a zooarchaeological and taphonomic point of view to infer the behaviour of the Neanderthals who occupied this cave during the formation of this level. Other complementary aspects are also analysed, such as the site's stratigraphy, its dating, pollen associations, the lithic industry, and the evidence of fire and human fossils.
Research sample	The research sample includes the entire set of faunal remains recovered from Level 3 of Cueva Des-Cubierta and, particularly, the cranial remains of large herbivores in this assemblage. For comparative purposes, the faunal association of Level 2 (overlying) has also been analysed to make the peculiar anatomical representation of level 3 more evident. Complementarily, other records from the site have been analysed, such as the lithic industry assemblages from Levels 2 and 3, the evidence of thermal alteration from Level 3, the pollen associations from Levels 5, 3 and 2, and the human remains from Level 2.
Sampling strategy	Archaeological records were recovered as the excavation of Cueva Des-Cubierta progressed (from top to bottom).
Data collection	All records from the excavation campaigns carried out between 2009 and 2018 were analysed as they were retrieved and incorporated into databases that have served as the basis for subsequent analyses. Some records from more recent campaigns have also been included in the study because of their particular interest.
Timing and spatial scale	Excavations at Cueva Des-Cubierta began in 2009, and have continued until the present day with an annual campaign lasting approximately one month. The levels analysed (2 and 3) are chronologically located between 40-80 ka ago.
Data exclusions	No data were excluded from the analyses.
Reproducibility	All relevant data used were fully documented and described in the supplementary material. To ensure the reproducibility of the experimental butchering of the cow heads, all the process has been described in detail.
Randomization	Randomisation was not relevant for this study because the entire sample of zooarchaeological remains from Level 3 was analysed.

March 2021

Blinding	During the experimental butchering of the cow heads, the different butchers were isolated from each other in order to avoid the influence of one on the work of the others.

Did the study involve field work? ☒ Yes ☐ No

Field work, collection and transport

Field conditions	The excavation of Cueva Des-Cubierta took place during the summers of 2009 to 2022. During the excavation campaigns, the site was covered with a shade canopy to prevent sunlight from falling on the archaeological artefacts and to make the outdoor work more bearable in this region's high temperatures typical at this time of year.
Location	The Cueva Des-Cubierta cave is located 70 km north of the city of Madrid in central Spain (40º55'23''N 3º48'29''W, WGS84 datum; altitude 1112 m).
Access & import/export	The excavated sites are legally protected. For their excavation and sampling, the necessary permits were always obtained from the competent authorities. No import/export actions were needed for this study.
Disturbance	Landscape disturbance caused by site excavation has been minimised wherever possible, e.g. by avoiding the accumulation of spoil in the vicinity of the sites by removing it in containers, and by using natural materials (wood, stone) wherever possible in the construction of enclosures. Visual impact has also been limited by the use of canopies in colours similar to those of the surrounding rocks. Special care has been taken to avoid any damage to protected animal or plant species living around the sites, always following the advice of the nature rangers in charge of their conservation.

Reporting for specific materials, systems and methods

We require information from authors about some types of materials, experimental systems and methods used in many studies. Here, indicate whether each material, system or method listed is relevant to your study. If you are not sure if a list item applies to your research, read the appropriate section before selecting a response.

Materials & experimental systems

n/a	Involved in the study	
☒	☐	Antibodies
☒	☐	Eukaryotic cell lines
☐	☒	Palaeontology and archaeology
☒	☐	Animals and other organisms
☒	☐	Clinical data
☒	☐	Dual use research of concern

Methods

n/a	Involved in the study	
☒	☐	ChIP-seq
☒	☐	Flow cytometry
☒	☐	MRI-based neuroimaging

Palaeontology and Archaeology

Specimen provenance	The archaeological and palaeontological materials studied in this work come from the excavation campaigns carried out at the Pinilla del Valle sites (Community of Madrid, Spain) from 2009 to 2022. These excavations have been authorised annually by the Dirección General de Patrimonio de la Comunidad de Madrid.
Specimen deposition	The materials studied are deposited in the Museo Arqueológico y Paleontológico de la Comunidad de Madrid (Alcalá de Henares, Madrid, Spain).
Dating methods	This work includes previously unpublished dating. The dated samples come from the Cueva Des-Cubierta (Pinilla del Valle, Madrid, Spain). They have been obtained directly from the site (speleothems) or selected from the excavation materials (charcoal and bones). The speleothem samples have been analysed at the Uranium Series Laboratory of CENIEH (Burgos, Spain) and also at the Geochronology Laboratory of the Instituto de Ciencias de la Tierra Jaume Almera (CSIC) (Barcelona, Spain), being processed by the respective technicians of each laboratory and following their protocols and quality controls. The radiocarbon samples have been analysed by the Oxford Radiocarbon Accelerator Unit (ORAU) (United Kingdom) and processed by the laboratory technicians following their protocols and quality controls. The dates have been calibrated using the IntCal20 calibration curve with the OxCal v.4.4 software.

☒ Tick this box to confirm that the raw and calibrated dates are available in the paper or in Supplementary Information.

Ethics oversight	No ethical aproval or guidance was required

Note that full information on the approval of the study protocol must also be provided in the manuscript.
